# Correction: BRET-based RAS biosensors that show a novel small molecule is an inhibitor of RAS-effector protein-protein interactions

**DOI:** 10.7554/eLife.40515

**Published:** 2018-08-02

**Authors:** Nicolas Bery, Abimael Cruz-Migoni, Carole JR Bataille, Camilo E Quevedo, Hanna Tulmin, Ami Miller, Angela Russell, Simon EV Phillips, Stephen B Carr, Terence H Rabbitts

Bery N, Cruz-Migoni A, Bataille CJR, Quevedo CE, Tulmin H, Miller A, Russell A, Phillips SEV, Carr SB, Rabbitts TH. 2018. BRET-based RAS biosensors that show a novel small molecule is an inhibitor of RAS-effector protein-protein interactions. *eLife* **7**:e37122. doi: 10.7554/eLife.37122.

Published 10, July 2018

An error was identified in Figure 1—figure supplement 3, panel E. The image showing the WaterLOGSY competition experiment against KRAS with and without the presence of the scFv mistakenly shows a regioisomer of compound 3344 and not 3344 itself. The error occurred during figure assembly in PowerPoint. The accidental change in the original published figure does not affect the results and conclusions of the original paper. Both compound 3344 and its regioisomer show the same competition behavior in these experiments.

The corrected Figure 1—figure supplement 3 is shown here.

**Figure 1:**
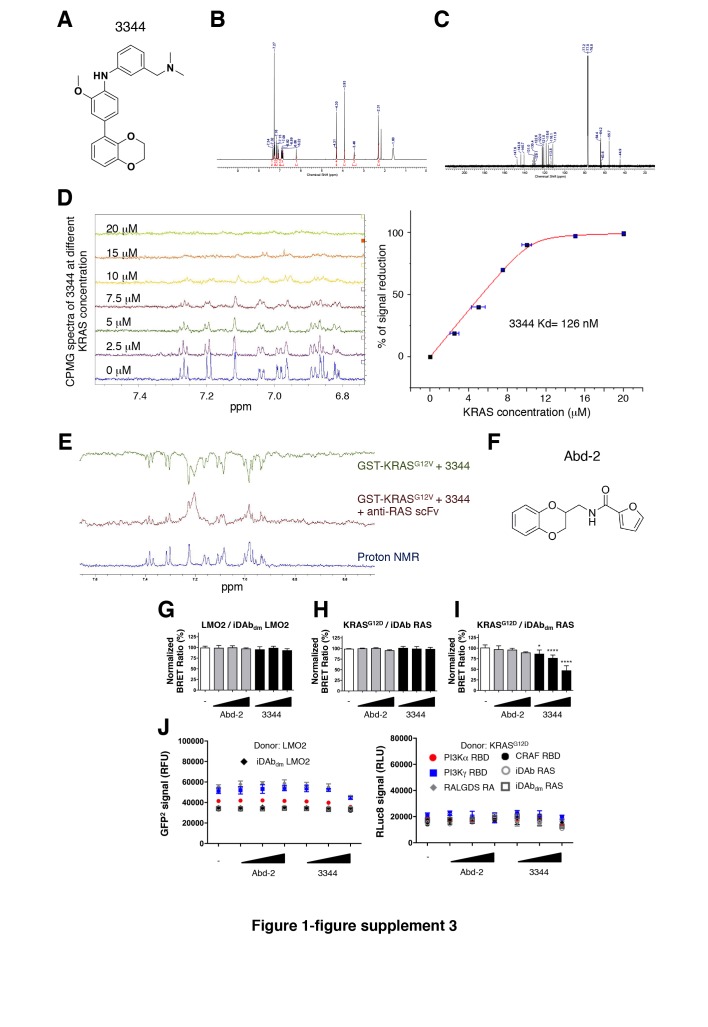


The originally published Figure 1—figure supplement 3 is also shown for reference:

**Figure 2:**
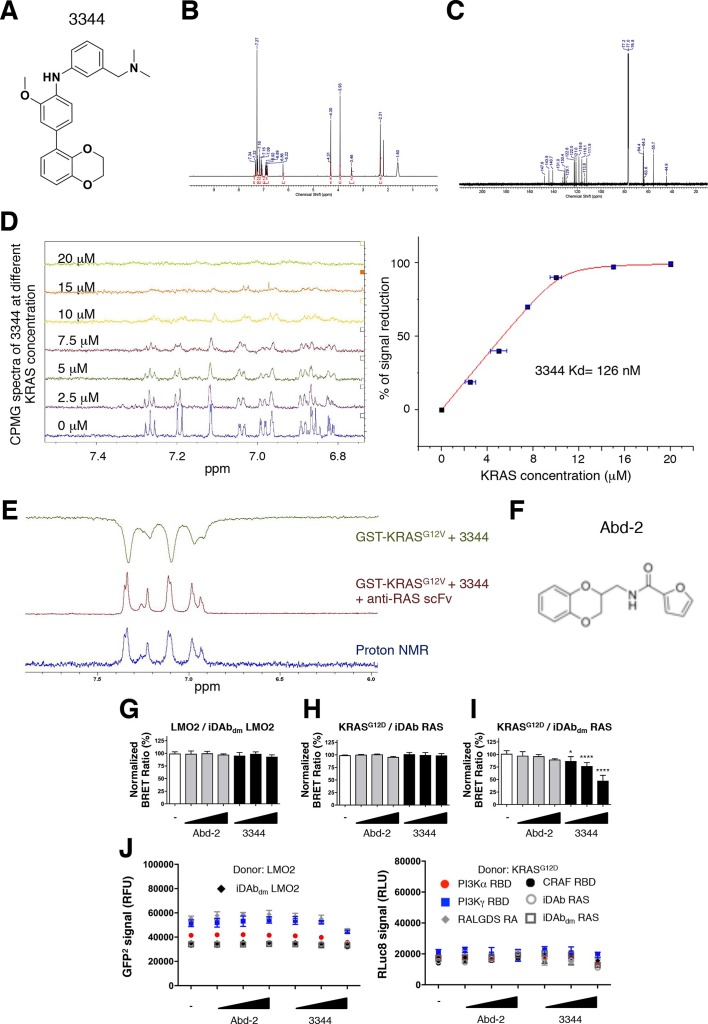


The article has been corrected accordingly.

