# Supplementary material for: BRET-based RAS biosensors that show a novel small molecule is an inhibitor of RAS-effector protein-protein interactions
Source: eLife. 2018 Jul 10;7:e37122. doi: 10.7554/eLife.37122 (PMC6039175; doi:10.7554/eLife.37122)
Supplement: Supplementary file 1. — The list of the DNA and protein sequences from the different RAS BRET biosensor constructs used in this study. [file elife-37122-supp1.docx]

Supplementary file 1: DNA and protein sequences of BRET biosensors constructs

Sequence: GFP2-iDAbdm control Range: 1 to 1209

10 20 30 40 50 60 70 80 90 100

ATGGTGAGCAAGGGCGAGGAGCTGTTCACCGGGGTGGTGCCCATCCTGGTCGAGCTGGACGGCGACGTAAACGGCCACAAGTTCAGCGTGTCCGGCGAGG

TACCACTCGTTCCCGCTCCTCGACAAGTGGCCCCACCACGGGTAGGACCAGCTCGACCTGCCGCTGCATTTGCCGGTGTTCAAGTCGCACAGGCCGCTCC

M V S K G E E L F T G V V P I L V E L D G D V N G H K F S V S G E>

______________________________TRANSLATION OF GFP2-IDABDM CONTROL [A]________________________________>

110 120 130 140 150 160 170 180 190 200

GCGAGGGCGATGCCACCTACGGCAAGCTGACCCTGAAGTTCATCTGCACCACCGGCAAGCTGCCCGTGCCCTGGCCCACCCTCGTGACCACCCTGAGCTA

CGCTCCCGCTACGGTGGATGCCGTTCGACTGGGACTTCAAGTAGACGTGGTGGCCGTTCGACGGGCACGGGACCGGGTGGGAGCACTGGTGGGACTCGAT

G E G D A T Y G K L T L K F I C T T G K L P V P W P T L V T T L S Y>

______________________________TRANSLATION OF GFP2-IDABDM CONTROL [A]________________________________>

210 220 230 240 250 260 270 280 290 300

CGGCGTGCAGTGCTTCAGCCGCTACCCCGACCACATGAAGCAGCACGACTTCTTCAAGTCCGCCATGCCCGAAGGCTACGTCCAGGAGCGCACCATCTTC

GCCGCACGTCACGAAGTCGGCGATGGGGCTGGTGTACTTCGTCGTGCTGAAGAAGTTCAGGCGGTACGGGCTTCCGATGCAGGTCCTCGCGTGGTAGAAG

G V Q C F S R Y P D H M K Q H D F F K S A M P E G Y V Q E R T I F>

______________________________TRANSLATION OF GFP2-IDABDM CONTROL [A]________________________________>

310 320 330 340 350 360 370 380 390 400

TTCAAGGACGACGGCAACTACAAGACCCGCGCCGAGGTGAAGTTCGAGGGCGACACCCTGGTGAACCGCATCGAGCTGAAGGGCATCGACTTCAAGGAGG

AAGTTCCTGCTGCCGTTGATGTTCTGGGCGCGGCTCCACTTCAAGCTCCCGCTGTGGGACCACTTGGCGTAGCTCGACTTCCCGTAGCTGAAGTTCCTCC

F K D D G N Y K T R A E V K F E G D T L V N R I E L K G I D F K E>

______________________________TRANSLATION OF GFP2-IDABDM CONTROL [A]________________________________>

410 420 430 440 450 460 470 480 490 500

ACGGCAACATCCTGGGGCACAAGCTGGAGTACAACTACAACAGCCACAACGTCTATATCATGGCCGACAAGCAGAAGAACGGCATCAAGGTGAACTTCAA

TGCCGTTGTAGGACCCCGTGTTCGACCTCATGTTGATGTTGTCGGTGTTGCAGATATAGTACCGGCTGTTCGTCTTCTTGCCGTAGTTCCACTTGAAGTT

D G N I L G H K L E Y N Y N S H N V Y I M A D K Q K N G I K V N F K>

______________________________TRANSLATION OF GFP2-IDABDM CONTROL [A]________________________________>

510 520 530 540 550 560 570 580 590 600

GATCCGCCACAACATCGAGGACGGCAGCGTGCAGCTCGCCGACCACTACCAGCAGAACACCCCCATCGGCGACGGCCCCGTGCTGCTGCCCGACAACCAC

CTAGGCGGTGTTGTAGCTCCTGCCGTCGCACGTCGAGCGGCTGGTGATGGTCGTCTTGTGGGGGTAGCCGCTGCCGGGGCACGACGACGGGCTGTTGGTG

I R H N I E D G S V Q L A D H Y Q Q N T P I G D G P V L L P D N H>

______________________________TRANSLATION OF GFP2-IDABDM CONTROL [A]________________________________>

610 620 630 640 650 660 670 680 690 700

TACCTGAGCACCCAGTCCGCCCTGAGCAAAGACCCCAACGAGAAGCGCGATCACATGGTCCTGCTGGAGTTCGTGACCGCCGCCGGGATCACTCTCAGCA

ATGGACTCGTGGGTCAGGCGGGACTCGTTTCTGGGGTTGCTCTTCGCGCTAGTGTACCAGGACGACCTCAAGCACTGGCGGCGGCCCTAGTGAGAGTCGT

Y L S T Q S A L S K D P N E K R D H M V L L E F V T A A G I T L S>

______________________________TRANSLATION OF GFP2-IDABDM CONTROL [A]________________________________>

710 720 730 740 750 760 770 780 790 800

TGGACGAGCTGTACAAGCTCGAGGGCGGCGGAGGATCTGGGGGCGGAGGAAGTGGGGGAGGGGGCTCTGCGGCCCGTATGGCCGAGGTGCAGCTGTTGGA

ACCTGCTCGACATGTTCGAGCTCCCGCCGCCTCCTAGACCCCCGCCTCCTTCACCCCCTCCCCCGAGACGCCGGGCATACCGGCTCCACGTCGACAACCT

M D E L Y K L E G G G G S G G G G S G G G G S A A R M A E V Q L L E>

______________________________TRANSLATION OF GFP2-IDABDM CONTROL [A]________________________________>

810 820 830 840 850 860 870 880 890 900

GTCTGGGGGAGGCTTGGTACAGCCTGGGGGGTCCCTGAGACTCTCCTGTGCAGCCTCTGGATTCAGCTTCAGTCATAGTCCTATGAATTGGGTCCGCCAG

CAGACCCCCTCCGAACCATGTCGGACCCCCCAGGGACTCTGAGAGGACACGTCGGAGACCTAAGTCGAAGTCAGTATCAGGATACTTAACCCAGGCGGTC

S G G G L V Q P G G S L R L S C A A S G F S F S H S P M N W V R Q>

______________________________TRANSLATION OF GFP2-IDABDM CONTROL [A]________________________________>

910 920 930 940 950 960 970 980 990 1000

GCTCCAGGGAAGGGGCTGGAGTGGGTTTCATACATTAGTTATAATGCTTCGAGTATATACTATGCAGACTCTGTGAAGGGCCGATTCACCATCTCCAGAG

CGAGGTCCCTTCCCCGACCTCACCCAAAGTATGTAATCAATATTACGAAGCTCATATATGATACGTCTGAGACACTTCCCGGCTAAGTGGTAGAGGTCTC

A P G K G L E W V S Y I S Y N A S S I Y Y A D S V K G R F T I S R>

______________________________TRANSLATION OF GFP2-IDABDM CONTROL [A]________________________________>

1010 1020 1030 1040 1050 1060 1070 1080 1090 1100

ACAATTCCAAGAACACACTGTATCTGCAAATGAACAGCCTGAGAGCCGAGGACACGGCTGTCTATTACTGTGCGAGAGGGTTGACGGAGTCTCTTGAGTT

TGTTAAGGTTCTTGTGTGACATAGACGTTTACTTGTCGGACTCTCGGCTCCTGTGCCGACAGATAATGACACGCTCTCCCAACTGCCTCAGAGAACTCAA

D N S K N T L Y L Q M N S L R A E D T A V Y Y C A R G L T E S L E L>

______________________________TRANSLATION OF GFP2-IDABDM CONTROL [A]________________________________>

1110 1120 1130 1140 1150 1160 1170 1180 1190 1200

GGCGGCGGATTGGTTTGATTACTGGGGCCAGGGAACCCTGGTCACCGTCTCGAGCGCGGCCGCAGAACAAAAACTCATCTCAGAAGAGGATCTGAATGGG

CCGCCGCCTAACCAAACTAATGACCCCGGTCCCTTGGGACCAGTGGCAGAGCTCGCGCCGGCGTCTTGTTTTTGAGTAGAGTCTTCTCCTAGACTTACCC

A A D W F D Y W G Q G T L V T V S S A A A E Q K L I S E E D L N G>

______________________________TRANSLATION OF GFP2-IDABDM CONTROL [A]________________________________>

GCCGCATAG

CGGCGTATC

A A *>

_________>

Sequence: iDAb control-GFP2 Range: 1 to 1152

10 20 30 40 50 60 70 80 90 100

ATGGCCGAGGTGCAGCTGTTGGAGTCTGGGGGAGGCTTGGTACAGCCTGGGGGGTCCCTGAGACTCTCCTGTGCAGCCTCTGGATTCAGCTTCAGTCATA

TACCGGCTCCACGTCGACAACCTCAGACCCCCTCCGAACCATGTCGGACCCCCCAGGGACTCTGAGAGGACACGTCGGAGACCTAAGTCGAAGTCAGTAT

M A E V Q L L E S G G G L V Q P G G S L R L S C A A S G F S F S H>

_______________________________TRANSLATION OF IDAB CONTROL-GFP2 [A]_________________________________>

110 120 130 140 150 160 170 180 190 200

GTCCTATGAATTGGGTCCGCCAGGCTCCAGGGAAGGGGCTGGAGTGGGTTTCATACATTAGTTATAATTCTTCGAGTATATACTATGCAGACTCTGTGAA

CAGGATACTTAACCCAGGCGGTCCGAGGTCCCTTCCCCGACCTCACCCAAAGTATGTAATCAATATTAAGAAGCTCATATATGATACGTCTGAGACACTT

S P M N W V R Q A P G K G L E W V S Y I S Y N S S S I Y Y A D S V K>

_______________________________TRANSLATION OF IDAB CONTROL-GFP2 [A]_________________________________>

210 220 230 240 250 260 270 280 290 300

GGGCCGATTCACCATCTCCAGAGACAATTCCAAGAACACACTGTATCTGCAAATGAACAGCCTGAGAGCCGAGGACACGGCTGTCTATTACTGTGCGAGA

CCCGGCTAAGTGGTAGAGGTCTCTGTTAAGGTTCTTGTGTGACATAGACGTTTACTTGTCGGACTCTCGGCTCCTGTGCCGACAGATAATGACACGCTCT

G R F T I S R D N S K N T L Y L Q M N S L R A E D T A V Y Y C A R>

_______________________________TRANSLATION OF IDAB CONTROL-GFP2 [A]_________________________________>

310 320 330 340 350 360 370 380 390 400

GGGTTGACGGAGTCTCTTGAGTTGACGGCGGATTGGTTTGATTACTGGGGCCAGGGAACCCTGGTCACCGTTAGTTCTCTCGAGGGCGGAGGCGGATCTG

CCCAACTGCCTCAGAGAACTCAACTGCCGCCTAACCAAACTAATGACCCCGGTCCCTTGGGACCAGTGGCAATCAAGAGAGCTCCCGCCTCCGCCTAGAC

G L T E S L E L T A D W F D Y W G Q G T L V T V S S L E G G G G S>

_______________________________TRANSLATION OF IDAB CONTROL-GFP2 [A]_________________________________>

410 420 430 440 450 460 470 480 490 500

GCGGCGGAGGATCTGCGGCCGCAGGGAGTGGTATGGTGAGCAAGGGCGAGGAGCTGTTCACCGGGGTGGTGCCCATCCTGGTCGAGCTGGACGGCGACGT

CGCCGCCTCCTAGACGCCGGCGTCCCTCACCATACCACTCGTTCCCGCTCCTCGACAAGTGGCCCCACCACGGGTAGGACCAGCTCGACCTGCCGCTGCA

G G G G S A A A G S G M V S K G E E L F T G V V P I L V E L D G D V>

_______________________________TRANSLATION OF IDAB CONTROL-GFP2 [A]_________________________________>

510 520 530 540 550 560 570 580 590 600

AAACGGCCACAAGTTCAGCGTGTCCGGCGAGGGCGAGGGCGATGCCACCTACGGCAAGCTGACCCTGAAGTTCATCTGCACCACCGGCAAGCTGCCCGTG

TTTGCCGGTGTTCAAGTCGCACAGGCCGCTCCCGCTCCCGCTACGGTGGATGCCGTTCGACTGGGACTTCAAGTAGACGTGGTGGCCGTTCGACGGGCAC

N G H K F S V S G E G E G D A T Y G K L T L K F I C T T G K L P V>

_______________________________TRANSLATION OF IDAB CONTROL-GFP2 [A]_________________________________>

610 620 630 640 650 660 670 680 690 700

CCCTGGCCCACCCTCGTGACCACCCTGAGCTACGGCGTGCAGTGCTTCAGCCGCTACCCCGACCACATGAAGCAGCACGACTTCTTCAAGTCCGCCATGC

GGGACCGGGTGGGAGCACTGGTGGGACTCGATGCCGCACGTCACGAAGTCGGCGATGGGGCTGGTGTACTTCGTCGTGCTGAAGAAGTTCAGGCGGTACG

P W P T L V T T L S Y G V Q C F S R Y P D H M K Q H D F F K S A M>

_______________________________TRANSLATION OF IDAB CONTROL-GFP2 [A]_________________________________>

710 720 730 740 750 760 770 780 790 800

CCGAAGGCTACGTCCAGGAGCGCACCATCTTCTTCAAGGACGACGGCAACTACAAGACCCGCGCCGAGGTGAAGTTCGAGGGCGACACCCTGGTGAACCG

GGCTTCCGATGCAGGTCCTCGCGTGGTAGAAGAAGTTCCTGCTGCCGTTGATGTTCTGGGCGCGGCTCCACTTCAAGCTCCCGCTGTGGGACCACTTGGC

P E G Y V Q E R T I F F K D D G N Y K T R A E V K F E G D T L V N R>

_______________________________TRANSLATION OF IDAB CONTROL-GFP2 [A]_________________________________>

810 820 830 840 850 860 870 880 890 900

CATCGAGCTGAAGGGCATCGACTTCAAGGAGGACGGCAACATCCTGGGGCACAAGCTGGAGTACAACTACAACAGCCACAACGTCTATATCATGGCCGAC

GTAGCTCGACTTCCCGTAGCTGAAGTTCCTCCTGCCGTTGTAGGACCCCGTGTTCGACCTCATGTTGATGTTGTCGGTGTTGCAGATATAGTACCGGCTG

I E L K G I D F K E D G N I L G H K L E Y N Y N S H N V Y I M A D>

_______________________________TRANSLATION OF IDAB CONTROL-GFP2 [A]_________________________________>

910 920 930 940 950 960 970 980 990 1000

AAGCAGAAGAACGGCATCAAGGTGAACTTCAAGATCCGCCACAACATCGAGGACGGCAGCGTGCAGCTCGCCGACCACTACCAGCAGAACACCCCCATCG

TTCGTCTTCTTGCCGTAGTTCCACTTGAAGTTCTAGGCGGTGTTGTAGCTCCTGCCGTCGCACGTCGAGCGGCTGGTGATGGTCGTCTTGTGGGGGTAGC

K Q K N G I K V N F K I R H N I E D G S V Q L A D H Y Q Q N T P I>

_______________________________TRANSLATION OF IDAB CONTROL-GFP2 [A]_________________________________>

1010 1020 1030 1040 1050 1060 1070 1080 1090 1100

GCGACGGCCCCGTGCTGCTGCCCGACAACCACTACCTGAGCACCCAGTCCGCCCTGAGCAAAGACCCCAACGAGAAGCGCGATCACATGGTCCTGCTGGA

CGCTGCCGGGGCACGACGACGGGCTGTTGGTGATGGACTCGTGGGTCAGGCGGGACTCGTTTCTGGGGTTGCTCTTCGCGCTAGTGTACCAGGACGACCT

G D G P V L L P D N H Y L S T Q S A L S K D P N E K R D H M V L L E>

_______________________________TRANSLATION OF IDAB CONTROL-GFP2 [A]_________________________________>

1110 1120 1130 1140 1150

GTTCGTGACCGCCGCCGGGATCACTCTCAGCATGGACGAGCTGTACAAGTAA

CAAGCACTGGCGGCGGCCCTAGTGAGAGTCGTACCTGCTCGACATGTTCATT

F V T A A G I T L S M D E L Y K *>

_______TRANSLATION OF IDAB CONTROL-GFP2 [A]_________>

Sequence: iDAb RAS-GFP2 Range: 1 to 1125

10 20 30 40 50 60 70 80 90 100

ATGGCCGAGGTGCAGCTGTTGGAGTCTGGGGGAGGCTTGGTACAGCCTGGGGGGTCCCTGAGACTCTCCTGTGCAGCCTCTGGATTCACCTTTAGTACCT

TACCGGCTCCACGTCGACAACCTCAGACCCCCTCCGAACCATGTCGGACCCCCCAGGGACTCTGAGAGGACACGTCGGAGACCTAAGTGGAAATCATGGA

M A E V Q L L E S G G G L V Q P G G S L R L S C A A S G F T F S T>

_________________________________TRANSLATION OF IDAB RAS-GFP2 [A]___________________________________>

110 120 130 140 150 160 170 180 190 200

TTAGCATGAACTGGGTCCGCCAGGCTCCAGGGAAGGGGCTGGAGTGGGTTTCATACATTAGTAGGACGTCGAAGACGATATACTATGCAGACTCTGTGAA

AATCGTACTTGACCCAGGCGGTCCGAGGTCCCTTCCCCGACCTCACCCAAAGTATGTAATCATCCTGCAGCTTCTGCTATATGATACGTCTGAGACACTT

F S M N W V R Q A P G K G L E W V S Y I S R T S K T I Y Y A D S V K>

_________________________________TRANSLATION OF IDAB RAS-GFP2 [A]___________________________________>

210 220 230 240 250 260 270 280 290 300

GGGCCGATTCACCATCTCCAGAGACAATTCCAAGAACACACTGTATCTGCAAATGAACAGCCTGAGAGCCGAGGACACGGCTGTCTATTACTGTGCGAGA

CCCGGCTAAGTGGTAGAGGTCTCTGTTAAGGTTCTTGTGTGACATAGACGTTTACTTGTCGGACTCTCGGCTCCTGTGCCGACAGATAATGACACGCTCT

G R F T I S R D N S K N T L Y L Q M N S L R A E D T A V Y Y C A R>

_________________________________TRANSLATION OF IDAB RAS-GFP2 [A]___________________________________>

310 320 330 340 350 360 370 380 390 400

GGGAGATTCTTTGACTACTGGGGCCAGGGAACCCTGGTCACCGTTAGTTCTCTCGAGGGCGGAGGCGGATCTGGCGGCGGAGGATCTGCGGCCGCAGGGA

CCCTCTAAGAAACTGATGACCCCGGTCCCTTGGGACCAGTGGCAATCAAGAGAGCTCCCGCCTCCGCCTAGACCGCCGCCTCCTAGACGCCGGCGTCCCT

G R F F D Y W G Q G T L V T V S S L E G G G G S G G G G S A A A G>

_________________________________TRANSLATION OF IDAB RAS-GFP2 [A]___________________________________>

410 420 430 440 450 460 470 480 490 500

GTGGTATGGTGAGCAAGGGCGAGGAGCTGTTCACCGGGGTGGTGCCCATCCTGGTCGAGCTGGACGGCGACGTAAACGGCCACAAGTTCAGCGTGTCCGG

CACCATACCACTCGTTCCCGCTCCTCGACAAGTGGCCCCACCACGGGTAGGACCAGCTCGACCTGCCGCTGCATTTGCCGGTGTTCAAGTCGCACAGGCC

S G M V S K G E E L F T G V V P I L V E L D G D V N G H K F S V S G>

_________________________________TRANSLATION OF IDAB RAS-GFP2 [A]___________________________________>

510 520 530 540 550 560 570 580 590 600

CGAGGGCGAGGGCGATGCCACCTACGGCAAGCTGACCCTGAAGTTCATCTGCACCACCGGCAAGCTGCCCGTGCCCTGGCCCACCCTCGTGACCACCCTG

GCTCCCGCTCCCGCTACGGTGGATGCCGTTCGACTGGGACTTCAAGTAGACGTGGTGGCCGTTCGACGGGCACGGGACCGGGTGGGAGCACTGGTGGGAC

E G E G D A T Y G K L T L K F I C T T G K L P V P W P T L V T T L>

_________________________________TRANSLATION OF IDAB RAS-GFP2 [A]___________________________________>

610 620 630 640 650 660 670 680 690 700

AGCTACGGCGTGCAGTGCTTCAGCCGCTACCCCGACCACATGAAGCAGCACGACTTCTTCAAGTCCGCCATGCCCGAAGGCTACGTCCAGGAGCGCACCA

TCGATGCCGCACGTCACGAAGTCGGCGATGGGGCTGGTGTACTTCGTCGTGCTGAAGAAGTTCAGGCGGTACGGGCTTCCGATGCAGGTCCTCGCGTGGT

S Y G V Q C F S R Y P D H M K Q H D F F K S A M P E G Y V Q E R T>

_________________________________TRANSLATION OF IDAB RAS-GFP2 [A]___________________________________>

710 720 730 740 750 760 770 780 790 800

TCTTCTTCAAGGACGACGGCAACTACAAGACCCGCGCCGAGGTGAAGTTCGAGGGCGACACCCTGGTGAACCGCATCGAGCTGAAGGGCATCGACTTCAA

AGAAGAAGTTCCTGCTGCCGTTGATGTTCTGGGCGCGGCTCCACTTCAAGCTCCCGCTGTGGGACCACTTGGCGTAGCTCGACTTCCCGTAGCTGAAGTT

I F F K D D G N Y K T R A E V K F E G D T L V N R I E L K G I D F K>

_________________________________TRANSLATION OF IDAB RAS-GFP2 [A]___________________________________>

810 820 830 840 850 860 870 880 890 900

GGAGGACGGCAACATCCTGGGGCACAAGCTGGAGTACAACTACAACAGCCACAACGTCTATATCATGGCCGACAAGCAGAAGAACGGCATCAAGGTGAAC

CCTCCTGCCGTTGTAGGACCCCGTGTTCGACCTCATGTTGATGTTGTCGGTGTTGCAGATATAGTACCGGCTGTTCGTCTTCTTGCCGTAGTTCCACTTG

E D G N I L G H K L E Y N Y N S H N V Y I M A D K Q K N G I K V N>

_________________________________TRANSLATION OF IDAB RAS-GFP2 [A]___________________________________>

910 920 930 940 950 960 970 980 990 1000

TTCAAGATCCGCCACAACATCGAGGACGGCAGCGTGCAGCTCGCCGACCACTACCAGCAGAACACCCCCATCGGCGACGGCCCCGTGCTGCTGCCCGACA

AAGTTCTAGGCGGTGTTGTAGCTCCTGCCGTCGCACGTCGAGCGGCTGGTGATGGTCGTCTTGTGGGGGTAGCCGCTGCCGGGGCACGACGACGGGCTGT

F K I R H N I E D G S V Q L A D H Y Q Q N T P I G D G P V L L P D>

_________________________________TRANSLATION OF IDAB RAS-GFP2 [A]___________________________________>

1010 1020 1030 1040 1050 1060 1070 1080 1090 1100

ACCACTACCTGAGCACCCAGTCCGCCCTGAGCAAAGACCCCAACGAGAAGCGCGATCACATGGTCCTGCTGGAGTTCGTGACCGCCGCCGGGATCACTCT

TGGTGATGGACTCGTGGGTCAGGCGGGACTCGTTTCTGGGGTTGCTCTTCGCGCTAGTGTACCAGGACGACCTCAAGCACTGGCGGCGGCCCTAGTGAGA

N H Y L S T Q S A L S K D P N E K R D H M V L L E F V T A A G I T L>

_________________________________TRANSLATION OF IDAB RAS-GFP2 [A]___________________________________>

1110 1120

CAGCATGGACGAGCTGTACAAGTAA

GTCGTACCTGCTCGACATGTTCATT

S M D E L Y K *>

___TRANSLATION OF IDA____>

Sequence: iDAbdm RAS-GFP2 Range: 1 to 1125

10 20 30 40 50 60 70 80 90 100

ATGGCCGAGGTGCAGCTGTTGGAGTCTGGGGGAGGCTTGGTACAGCCTGGGGGGTCCCTGAGACTCTCCTGTGCAGCCTCTGGATTCGCCTTTGCTGCCT

TACCGGCTCCACGTCGACAACCTCAGACCCCCTCCGAACCATGTCGGACCCCCCAGGGACTCTGAGAGGACACGTCGGAGACCTAAGCGGAAACGACGGA

M A E V Q L L E S G G G L V Q P G G S L R L S C A A S G F A F A A>

________________________________TRANSLATION OF IDABDM RAS-GFP2 [A]__________________________________>

110 120 130 140 150 160 170 180 190 200

TTAGCATGAACTGGGTCCGCCAGGCTCCAGGGAAGGGGCTGGAGTGGGTTTCATACATTAGTAGGACGTCGAAGACGATATACTATGCAGACTCTGTGAA

AATCGTACTTGACCCAGGCGGTCCGAGGTCCCTTCCCCGACCTCACCCAAAGTATGTAATCATCCTGCAGCTTCTGCTATATGATACGTCTGAGACACTT

F S M N W V R Q A P G K G L E W V S Y I S R T S K T I Y Y A D S V K>

________________________________TRANSLATION OF IDABDM RAS-GFP2 [A]__________________________________>

210 220 230 240 250 260 270 280 290 300

GGGCCGATTCACCATCTCCAGAGACAATTCCAAGAACACACTGTATCTGCAAATGAACAGCCTGAGAGCCGAGGACACGGCTGTCTATTACTGTGCGAGA

CCCGGCTAAGTGGTAGAGGTCTCTGTTAAGGTTCTTGTGTGACATAGACGTTTACTTGTCGGACTCTCGGCTCCTGTGCCGACAGATAATGACACGCTCT

G R F T I S R D N S K N T L Y L Q M N S L R A E D T A V Y Y C A R>

________________________________TRANSLATION OF IDABDM RAS-GFP2 [A]__________________________________>

310 320 330 340 350 360 370 380 390 400

GGGGGAGGCTTTGACTACTGGGGCCAGGGAACCCTGGTCACCGTTAGTTCTCTCGAGGGCGGAGGCGGATCTGGCGGCGGAGGATCTGCGGCCGCAGGGA

CCCCCTCCGAAACTGATGACCCCGGTCCCTTGGGACCAGTGGCAATCAAGAGAGCTCCCGCCTCCGCCTAGACCGCCGCCTCCTAGACGCCGGCGTCCCT

G G G F D Y W G Q G T L V T V S S L E G G G G S G G G G S A A A G>

________________________________TRANSLATION OF IDABDM RAS-GFP2 [A]__________________________________>

410 420 430 440 450 460 470 480 490 500

GTGGTATGGTGAGCAAGGGCGAGGAGCTGTTCACCGGGGTGGTGCCCATCCTGGTCGAGCTGGACGGCGACGTAAACGGCCACAAGTTCAGCGTGTCCGG

CACCATACCACTCGTTCCCGCTCCTCGACAAGTGGCCCCACCACGGGTAGGACCAGCTCGACCTGCCGCTGCATTTGCCGGTGTTCAAGTCGCACAGGCC

S G M V S K G E E L F T G V V P I L V E L D G D V N G H K F S V S G>

________________________________TRANSLATION OF IDABDM RAS-GFP2 [A]__________________________________>

510 520 530 540 550 560 570 580 590 600

CGAGGGCGAGGGCGATGCCACCTACGGCAAGCTGACCCTGAAGTTCATCTGCACCACCGGCAAGCTGCCCGTGCCCTGGCCCACCCTCGTGACCACCCTG

GCTCCCGCTCCCGCTACGGTGGATGCCGTTCGACTGGGACTTCAAGTAGACGTGGTGGCCGTTCGACGGGCACGGGACCGGGTGGGAGCACTGGTGGGAC

E G E G D A T Y G K L T L K F I C T T G K L P V P W P T L V T T L>

________________________________TRANSLATION OF IDABDM RAS-GFP2 [A]__________________________________>

610 620 630 640 650 660 670 680 690 700

AGCTACGGCGTGCAGTGCTTCAGCCGCTACCCCGACCACATGAAGCAGCACGACTTCTTCAAGTCCGCCATGCCCGAAGGCTACGTCCAGGAGCGCACCA

TCGATGCCGCACGTCACGAAGTCGGCGATGGGGCTGGTGTACTTCGTCGTGCTGAAGAAGTTCAGGCGGTACGGGCTTCCGATGCAGGTCCTCGCGTGGT

S Y G V Q C F S R Y P D H M K Q H D F F K S A M P E G Y V Q E R T>

________________________________TRANSLATION OF IDABDM RAS-GFP2 [A]__________________________________>

710 720 730 740 750 760 770 780 790 800

TCTTCTTCAAGGACGACGGCAACTACAAGACCCGCGCCGAGGTGAAGTTCGAGGGCGACACCCTGGTGAACCGCATCGAGCTGAAGGGCATCGACTTCAA

AGAAGAAGTTCCTGCTGCCGTTGATGTTCTGGGCGCGGCTCCACTTCAAGCTCCCGCTGTGGGACCACTTGGCGTAGCTCGACTTCCCGTAGCTGAAGTT

I F F K D D G N Y K T R A E V K F E G D T L V N R I E L K G I D F K>

________________________________TRANSLATION OF IDABDM RAS-GFP2 [A]__________________________________>

810 820 830 840 850 860 870 880 890 900

GGAGGACGGCAACATCCTGGGGCACAAGCTGGAGTACAACTACAACAGCCACAACGTCTATATCATGGCCGACAAGCAGAAGAACGGCATCAAGGTGAAC

CCTCCTGCCGTTGTAGGACCCCGTGTTCGACCTCATGTTGATGTTGTCGGTGTTGCAGATATAGTACCGGCTGTTCGTCTTCTTGCCGTAGTTCCACTTG

E D G N I L G H K L E Y N Y N S H N V Y I M A D K Q K N G I K V N>

________________________________TRANSLATION OF IDABDM RAS-GFP2 [A]__________________________________>

910 920 930 940 950 960 970 980 990 1000

TTCAAGATCCGCCACAACATCGAGGACGGCAGCGTGCAGCTCGCCGACCACTACCAGCAGAACACCCCCATCGGCGACGGCCCCGTGCTGCTGCCCGACA

AAGTTCTAGGCGGTGTTGTAGCTCCTGCCGTCGCACGTCGAGCGGCTGGTGATGGTCGTCTTGTGGGGGTAGCCGCTGCCGGGGCACGACGACGGGCTGT

F K I R H N I E D G S V Q L A D H Y Q Q N T P I G D G P V L L P D>

________________________________TRANSLATION OF IDABDM RAS-GFP2 [A]__________________________________>

1010 1020 1030 1040 1050 1060 1070 1080 1090 1100

ACCACTACCTGAGCACCCAGTCCGCCCTGAGCAAAGACCCCAACGAGAAGCGCGATCACATGGTCCTGCTGGAGTTCGTGACCGCCGCCGGGATCACTCT

TGGTGATGGACTCGTGGGTCAGGCGGGACTCGTTTCTGGGGTTGCTCTTCGCGCTAGTGTACCAGGACGACCTCAAGCACTGGCGGCGGCCCTAGTGAGA

N H Y L S T Q S A L S K D P N E K R D H M V L L E F V T A A G I T L>

________________________________TRANSLATION OF IDABDM RAS-GFP2 [A]__________________________________>

1110 1120

CAGCATGGACGAGCTGTACAAGTAA

GTCGTACCTGCTCGACATGTTCATT

S M D E L Y K *>

___TRANSLATION OF IDA____>

Sequence: membrane bound FLAG-iDAb control-myc competitor Range: 1 to 528

10 20 30 40 50 60 70 80 90 100

ATGCTGTGCTGTATGAGAAGAACCAAACAGGTTGAAAAGAATGATGAGGACCAAAAGATCGTCGACATGGACTACAAGGACGACGATGACAGGCCCATGG

TACGACACGACATACTCTTCTTGGTTTGTCCAACTTTTCTTACTACTCCTGGTTTTCTAGCAGCTGTACCTGATGTTCCTGCTGCTACTGTCCGGGTACC

M L C C M R R T K Q V E K N D E D Q K I V D M D Y K D D D D R P M>

________________TRANSLATION OF MEMBRANE BOUND FLAG-IDAB CONTROL-MYC COMPETITOR [A]__________________>

110 120 130 140 150 160 170 180 190 200

CCGAGGTGCAGCTGTTGGAGTCTGGGGGAGGCTTGGTACAGCCTGGGGGGTCCCTGAGACTCTCCTGTGCAGCCTCTGGATTCAGCTTCAGTCATAGTCC

GGCTCCACGTCGACAACCTCAGACCCCCTCCGAACCATGTCGGACCCCCCAGGGACTCTGAGAGGACACGTCGGAGACCTAAGTCGAAGTCAGTATCAGG

A E V Q L L E S G G G L V Q P G G S L R L S C A A S G F S F S H S P>

________________TRANSLATION OF MEMBRANE BOUND FLAG-IDAB CONTROL-MYC COMPETITOR [A]__________________>

210 220 230 240 250 260 270 280 290 300

TATGAATTGGGTCCGCCAGGCTCCAGGGAAGGGGCTGGAGTGGGTTTCATACATTAGTTATAATTCTTCGAGTATATACTATGCAGACTCTGTGAAGGGC

ATACTTAACCCAGGCGGTCCGAGGTCCCTTCCCCGACCTCACCCAAAGTATGTAATCAATATTAAGAAGCTCATATATGATACGTCTGAGACACTTCCCG

M N W V R Q A P G K G L E W V S Y I S Y N S S S I Y Y A D S V K G>

________________TRANSLATION OF MEMBRANE BOUND FLAG-IDAB CONTROL-MYC COMPETITOR [A]__________________>

310 320 330 340 350 360 370 380 390 400

CGATTCACCATCTCCAGAGACAATTCCAAGAACACACTGTATCTGCAAATGAACAGCCTGAGAGCCGAGGACACGGCTGTCTATTACTGTGCGAGAGGGT

GCTAAGTGGTAGAGGTCTCTGTTAAGGTTCTTGTGTGACATAGACGTTTACTTGTCGGACTCTCGGCTCCTGTGCCGACAGATAATGACACGCTCTCCCA

R F T I S R D N S K N T L Y L Q M N S L R A E D T A V Y Y C A R G>

________________TRANSLATION OF MEMBRANE BOUND FLAG-IDAB CONTROL-MYC COMPETITOR [A]__________________>

410 420 430 440 450 460 470 480 490 500

TGACGGAGTCTCTTGAGTTGACGGCGGATTGGTTTGATTACTGGGGCCAGGGAACCCTGGTCACCGTCTCGAGCGCGGCCGCAGAACAAAAACTCATCTC

ACTGCCTCAGAGAACTCAACTGCCGCCTAACCAAACTAATGACCCCGGTCCCTTGGGACCAGTGGCAGAGCTCGCGCCGGCGTCTTGTTTTTGAGTAGAG

L T E S L E L T A D W F D Y W G Q G T L V T V S S A A A E Q K L I S>

________________TRANSLATION OF MEMBRANE BOUND FLAG-IDAB CONTROL-MYC COMPETITOR [A]__________________>

510 520

AGAAGAGGATCTGAATGGGGCCGCATAG

TCTTCTCCTAGACTTACCCCGGCGTATC

E E D L N G A A *>

___TRANSLATION OF MEMBRA____>

Sequence: membrane bound FLAG-iDAb RAS-myc competitor Range: 1 to 501

10 20 30 40 50 60 70 80 90 100

ATGCTGTGCTGTATGAGAAGAACCAAACAGGTTGAAAAGAATGATGAGGACCAAAAGATCGTCGACATGGACTACAAAGACGACGATGACAGGCCCATGG

TACGACACGACATACTCTTCTTGGTTTGTCCAACTTTTCTTACTACTCCTGGTTTTCTAGCAGCTGTACCTGATGTTTCTGCTGCTACTGTCCGGGTACC

M L C C M R R T K Q V E K N D E D Q K I V D M D Y K D D D D R P M>

__________________TRANSLATION OF MEMBRANE BOUND FLAG-IDAB RAS-MYC COMPETITOR [A]____________________>

110 120 130 140 150 160 170 180 190 200

CCGAGGTGCAGCTGTTGGAGTCTGGGGGAGGCTTGGTACAGCCTGGGGGGTCCCTGAGACTCTCCTGTGCAGCCTCTGGATTCACCTTTAGTACCTTTAG

GGCTCCACGTCGACAACCTCAGACCCCCTCCGAACCATGTCGGACCCCCCAGGGACTCTGAGAGGACACGTCGGAGACCTAAGTGGAAATCATGGAAATC

A E V Q L L E S G G G L V Q P G G S L R L S C A A S G F T F S T F S>

__________________TRANSLATION OF MEMBRANE BOUND FLAG-IDAB RAS-MYC COMPETITOR [A]____________________>

210 220 230 240 250 260 270 280 290 300

CATGAACTGGGTCCGCCAGGCTCCAGGGAAGGGGCTGGAGTGGGTTTCATACATTAGTAGGACGTCGAAGACGATATACTATGCAGACTCTGTGAAGGGC

GTACTTGACCCAGGCGGTCCGAGGTCCCTTCCCCGACCTCACCCAAAGTATGTAATCATCCTGCAGCTTCTGCTATATGATACGTCTGAGACACTTCCCG

M N W V R Q A P G K G L E W V S Y I S R T S K T I Y Y A D S V K G>

__________________TRANSLATION OF MEMBRANE BOUND FLAG-IDAB RAS-MYC COMPETITOR [A]____________________>

310 320 330 340 350 360 370 380 390 400

CGATTCACCATCTCCAGAGACAATTCCAAGAACACACTGTATCTGCAAATGAACAGCCTGAGAGCCGAGGACACGGCTGTCTATTACTGTGCGAGAGGGA

GCTAAGTGGTAGAGGTCTCTGTTAAGGTTCTTGTGTGACATAGACGTTTACTTGTCGGACTCTCGGCTCCTGTGCCGACAGATAATGACACGCTCTCCCT

R F T I S R D N S K N T L Y L Q M N S L R A E D T A V Y Y C A R G>

__________________TRANSLATION OF MEMBRANE BOUND FLAG-IDAB RAS-MYC COMPETITOR [A]____________________>

410 420 430 440 450 460 470 480 490 500

GATTCTTTGACTACTGGGGCCAGGGAACCCTGGTCACCGTCTCGAGCGCGGCCGCAGAACAAAAACTCATCTCAGAAGAGGATCTGAATGGGGCCGCATA

CTAAGAAACTGATGACCCCGGTCCCTTGGGACCAGTGGCAGAGCTCGCGCCGGCGTCTTGTTTTTGAGTAGAGTCTTCTCCTAGACTTACCCCGGCGTAT

R F F D Y W G Q G T L V T V S S A A A E Q K L I S E E D L N G A A *>

__________________TRANSLATION OF MEMBRANE BOUND FLAG-IDAB RAS-MYC COMPETITOR [A]____________________>

G

C

_>

Sequence: iDAb control-myc competitor Range: 1 to 432

10 20 30 40 50 60 70 80 90 100

ATGGCCGAGGTGCAGCTGTTGGAGTCTGGGGGAGGCTTGGTACAGCCTGGGGGGTCCCTGAGACTCTCCTGTGCAGCCTCTGGATTCAGCTTCAGTCATA

TACCGGCTCCACGTCGACAACCTCAGACCCCCTCCGAACCATGTCGGACCCCCCAGGGACTCTGAGAGGACACGTCGGAGACCTAAGTCGAAGTCAGTAT

M A E V Q L L E S G G G L V Q P G G S L R L S C A A S G F S F S H>

__________________________TRANSLATION OF IDAB CONTROL-MYC COMPETITOR [A]____________________________>

110 120 130 140 150 160 170 180 190 200

GTCCTATGAATTGGGTCCGCCAGGCTCCAGGGAAGGGGCTGGAGTGGGTTTCATACATTAGTTATAATTCTTCGAGTATATACTATGCAGACTCTGTGAA

CAGGATACTTAACCCAGGCGGTCCGAGGTCCCTTCCCCGACCTCACCCAAAGTATGTAATCAATATTAAGAAGCTCATATATGATACGTCTGAGACACTT

S P M N W V R Q A P G K G L E W V S Y I S Y N S S S I Y Y A D S V K>

__________________________TRANSLATION OF IDAB CONTROL-MYC COMPETITOR [A]____________________________>

210 220 230 240 250 260 270 280 290 300

GGGCCGATTCACCATCTCCAGAGACAATTCCAAGAACACACTGTATCTGCAAATGAACAGCCTGAGAGCCGAGGACACGGCTGTCTATTACTGTGCGAGA

CCCGGCTAAGTGGTAGAGGTCTCTGTTAAGGTTCTTGTGTGACATAGACGTTTACTTGTCGGACTCTCGGCTCCTGTGCCGACAGATAATGACACGCTCT

G R F T I S R D N S K N T L Y L Q M N S L R A E D T A V Y Y C A R>

__________________________TRANSLATION OF IDAB CONTROL-MYC COMPETITOR [A]____________________________>

310 320 330 340 350 360 370 380 390 400

GGGTTGACGGAGTCTCTTGAGTTGACGGCGGATTGGTTTGATTACTGGGGCCAGGGAACCCTGGTCACCGTCTCGAGCGCGGCCGCAGAACAAAAACTCA

CCCAACTGCCTCAGAGAACTCAACTGCCGCCTAACCAAACTAATGACCCCGGTCCCTTGGGACCAGTGGCAGAGCTCGCGCCGGCGTCTTGTTTTTGAGT

G L T E S L E L T A D W F D Y W G Q G T L V T V S S A A A E Q K L>

__________________________TRANSLATION OF IDAB CONTROL-MYC COMPETITOR [A]____________________________>

410 420 430

TCTCAGAAGAGGATCTGAATGGGGCCGCATAG

AGAGTCTTCTCCTAGACTTACCCCGGCGTATC

I S E E D L N G A A *>

___TRANSLATION OF IDAB CONTR____>

Sequence: iDAb RAS-myc competitor Range: 1 to 405

10 20 30 40 50 60 70 80 90 100

ATGGCCGAGGTGCAGCTGTTGGAGTCTGGGGGAGGCTTGGTACAGCCTGGGGGGTCCCTGAGACTCTCCTGTGCAGCCTCTGGATTCACCTTTAGTACCT

TACCGGCTCCACGTCGACAACCTCAGACCCCCTCCGAACCATGTCGGACCCCCCAGGGACTCTGAGAGGACACGTCGGAGACCTAAGTGGAAATCATGGA

M A E V Q L L E S G G G L V Q P G G S L R L S C A A S G F T F S T>

____________________________TRANSLATION OF IDAB RAS-MYC COMPETITOR [A]______________________________>

110 120 130 140 150 160 170 180 190 200

TTAGCATGAACTGGGTCCGCCAGGCTCCAGGGAAGGGGCTGGAGTGGGTTTCATACATTAGTAGGACGTCGAAGACGATATACTATGCAGACTCTGTGAA

AATCGTACTTGACCCAGGCGGTCCGAGGTCCCTTCCCCGACCTCACCCAAAGTATGTAATCATCCTGCAGCTTCTGCTATATGATACGTCTGAGACACTT

F S M N W V R Q A P G K G L E W V S Y I S R T S K T I Y Y A D S V K>

____________________________TRANSLATION OF IDAB RAS-MYC COMPETITOR [A]______________________________>

210 220 230 240 250 260 270 280 290 300

GGGCCGATTCACCATCTCCAGAGACAATTCCAAGAACACACTGTATCTGCAAATGAACAGCCTGAGAGCCGAGGACACGGCTGTCTATTACTGTGCGAGA

CCCGGCTAAGTGGTAGAGGTCTCTGTTAAGGTTCTTGTGTGACATAGACGTTTACTTGTCGGACTCTCGGCTCCTGTGCCGACAGATAATGACACGCTCT

G R F T I S R D N S K N T L Y L Q M N S L R A E D T A V Y Y C A R>

____________________________TRANSLATION OF IDAB RAS-MYC COMPETITOR [A]______________________________>

310 320 330 340 350 360 370 380 390 400

GGGAGATTCTTTGACTACTGGGGCCAGGGAACCCTGGTCACCGTCTCGAGCGCGGCCGCAGAACAAAAACTCATCTCAGAAGAGGATCTGAATGGGGCCG

CCCTCTAAGAAACTGATGACCCCGGTCCCTTGGGACCAGTGGCAGAGCTCGCGCCGGCGTCTTGTTTTTGAGTAGAGTCTTCTCCTAGACTTACCCCGGC

G R F F D Y W G Q G T L V T V S S A A A E Q K L I S E E D L N G A>

____________________________TRANSLATION OF IDAB RAS-MYC COMPETITOR [A]______________________________>

CATAG

GTATC

A *>

_____>

Sequence: myc-p85alpha Range: 1 to 2217

10 20 30 40 50 60 70 80 90 100

ATGGAGCAGAAACTCATCTCTGAAGAGGATCTGGGCGGATCCATGAGTGCTGAGGGGTACCAGTACAGAGCGCTGTATGATTATAAAAAGGAAAGAGAAG

TACCTCGTCTTTGAGTAGAGACTTCTCCTAGACCCGCCTAGGTACTCACGACTCCCCATGGTCATGTCTCGCGACATACTAATATTTTTCCTTTCTCTTC

M E Q K L I S E E D L G G S M S A E G Y Q Y R A L Y D Y K K E R E>

__________________________________TRANSLATION OF MYC-P85ALPHA [A]___________________________________>

110 120 130 140 150 160 170 180 190 200

AAGATATTGACTTGCACTTGGGTGACATATTGACTGTGAATAAAGGGTCCTTAGTAGCTCTTGGATTCAGTGATGGACAGGAAGCCAGGCCTGAAGAAAT

TTCTATAACTGAACGTGAACCCACTGTATAACTGACACTTATTTCCCAGGAATCATCGAGAACCTAAGTCACTACCTGTCCTTCGGTCCGGACTTCTTTA

E D I D L H L G D I L T V N K G S L V A L G F S D G Q E A R P E E I>

__________________________________TRANSLATION OF MYC-P85ALPHA [A]___________________________________>

210 220 230 240 250 260 270 280 290 300

TGGCTGGTTAAATGGCTATAATGAAACCACAGGGGAAAGGGGGGACTTTCCGGGAACTTACGTAGAATATATTGGAAGGAAAAAAATCTCGCCTCCCACA

ACCGACCAATTTACCGATATTACTTTGGTGTCCCCTTTCCCCCCTGAAAGGCCCTTGAATGCATCTTATATAACCTTCCTTTTTTTAGAGCGGAGGGTGT

G W L N G Y N E T T G E R G D F P G T Y V E Y I G R K K I S P P T>

__________________________________TRANSLATION OF MYC-P85ALPHA [A]___________________________________>

310 320 330 340 350 360 370 380 390 400

CCAAAGCCCCGGCCACCTCGGCCTCTTCCTGTTGCACCAGGTTCTTCGAAAACTGAAGCAGATGTTGAACAACAAGCTTTGACTCTCCCGGATCTTGCAG

GGTTTCGGGGCCGGTGGAGCCGGAGAAGGACAACGTGGTCCAAGAAGCTTTTGACTTCGTCTACAACTTGTTGTTCGAAACTGAGAGGGCCTAGAACGTC

P K P R P P R P L P V A P G S S K T E A D V E Q Q A L T L P D L A>

__________________________________TRANSLATION OF MYC-P85ALPHA [A]___________________________________>

410 420 430 440 450 460 470 480 490 500

AGCAGTTTGCCCCTCCTGACATTGCCCCGCCTCTTCTTATCAAGCTCGTGGAAGCCATTGAAAAGAAAGGTCTGGAATGTTCAACTCTATACAGAACACA

TCGTCAAACGGGGAGGACTGTAACGGGGCGGAGAAGAATAGTTCGAGCACCTTCGGTAACTTTTCTTTCCAGACCTTACAAGTTGAGATATGTCTTGTGT

E Q F A P P D I A P P L L I K L V E A I E K K G L E C S T L Y R T Q>

__________________________________TRANSLATION OF MYC-P85ALPHA [A]___________________________________>

510 520 530 540 550 560 570 580 590 600

GAGCTCCAGCAACCTGGCAGAATTACGACAGCTTCTTGATTGTGATACACCCTCCGTGGACTTGGAAATGATCGATGTGCACGTTTTGGCTGACGCTTTC

CTCGAGGTCGTTGGACCGTCTTAATGCTGTCGAAGAACTAACACTATGTGGGAGGCACCTGAACCTTTACTAGCTACACGTGCAAAACCGACTGCGAAAG

S S S N L A E L R Q L L D C D T P S V D L E M I D V H V L A D A F>

__________________________________TRANSLATION OF MYC-P85ALPHA [A]___________________________________>

610 620 630 640 650 660 670 680 690 700

AAACGCTATCTCCTGGACTTACCAAATCCTGTCATTCCAGCAGCCGTTTACAGTGAAATGATTTCTTTAGCTCCAGAAGTACAAAGCTCCGAAGAATATA

TTTGCGATAGAGGACCTGAATGGTTTAGGACAGTAAGGTCGTCGGCAAATGTCACTTTACTAAAGAAATCGAGGTCTTCATGTTTCGAGGCTTCTTATAT

K R Y L L D L P N P V I P A A V Y S E M I S L A P E V Q S S E E Y>

__________________________________TRANSLATION OF MYC-P85ALPHA [A]___________________________________>

710 720 730 740 750 760 770 780 790 800

TTCAGCTATTGAAGAAGCTTATTAGGTCGCCTAGCATACCTCATCAGTATTGGCTTACGCTTCAGTATTTGTTAAAACATTTCTTCAAGCTCTCTCAAAC

AAGTCGATAACTTCTTCGAATAATCCAGCGGATCGTATGGAGTAGTCATAACCGAATGCGAAGTCATAAACAATTTTGTAAAGAAGTTCGAGAGAGTTTG

I Q L L K K L I R S P S I P H Q Y W L T L Q Y L L K H F F K L S Q T>

__________________________________TRANSLATION OF MYC-P85ALPHA [A]___________________________________>

810 820 830 840 850 860 870 880 890 900

CTCCAGCAAAAATCTGTTGAATGCAAGAGTACTCTCTGAAATTTTCAGCCCTATGCTTTTCAGATTCTCAGCAGCCAGCTCTGATAATACTGAAAACCTC

GAGGTCGTTTTTAGACAACTTACGTTCTCATGAGAGACTTTAAAAGTCGGGATACGAAAAGTCTAAGAGTCGTCGGTCGAGACTATTATGACTTTTGGAG

S S K N L L N A R V L S E I F S P M L F R F S A A S S D N T E N L>

__________________________________TRANSLATION OF MYC-P85ALPHA [A]___________________________________>

910 920 930 940 950 960 970 980 990 1000

ATAAAAGTTATAGAAATTTTAATCTCAACTGAATGGAATGAACGACAGCCTGCACCAGCACTGCCTCCTAAACCACCAAAACCTACTACTGTAGCCAACA

TATTTTCAATATCTTTAAAATTAGAGTTGACTTACCTTACTTGCTGTCGGACGTGGTCGTGACGGAGGATTTGGTGGTTTTGGATGATGACATCGGTTGT

I K V I E I L I S T E W N E R Q P A P A L P P K P P K P T T V A N>

__________________________________TRANSLATION OF MYC-P85ALPHA [A]___________________________________>

1010 1020 1030 1040 1050 1060 1070 1080 1090 1100

ACGGTATGAATAACAATATGTCCTTACAAGATGCTGAATGGTACTGGGGAGATATCTCGAGGGAAGAAGTGAATGAAAAACTTCGAGATACAGCAGACGG

TGCCATACTTATTGTTATACAGGAATGTTCTACGACTTACCATGACCCCTCTATAGAGCTCCCTTCTTCACTTACTTTTTGAAGCTCTATGTCGTCTGCC

N G M N N N M S L Q D A E W Y W G D I S R E E V N E K L R D T A D G>

__________________________________TRANSLATION OF MYC-P85ALPHA [A]___________________________________>

1110 1120 1130 1140 1150 1160 1170 1180 1190 1200

GACCTTTTTGGTACGAGATGCGTCTACTAAAATGCATGGTGATTATACTCTTACACTAAGGAAAGGGGGAAATAACAAATTAATCAAAATATTTCATCGA

CTGGAAAAACCATGCTCTACGCAGATGATTTTACGTACCACTAATATGAGAATGTGATTCCTTTCCCCCTTTATTGTTTAATTAGTTTTATAAAGTAGCT

T F L V R D A S T K M H G D Y T L T L R K G G N N K L I K I F H R>

__________________________________TRANSLATION OF MYC-P85ALPHA [A]___________________________________>

1210 1220 1230 1240 1250 1260 1270 1280 1290 1300

GATGGGAAATATGGCTTCTCTGACCCATTAACCTTCAGTTCTGTGGTTGAATTAATAAACCACTACCGGAATGAATCTCTAGCTCAGTATAATCCCAAAT

CTACCCTTTATACCGAAGAGACTGGGTAATTGGAAGTCAAGACACCAACTTAATTATTTGGTGATGGCCTTACTTAGAGATCGAGTCATATTAGGGTTTA

D G K Y G F S D P L T F S S V V E L I N H Y R N E S L A Q Y N P K>

__________________________________TRANSLATION OF MYC-P85ALPHA [A]___________________________________>

1310 1320 1330 1340 1350 1360 1370 1380 1390 1400

TGGATGTGAAATTACTTTATCCAGTATCCAAATACCAACAGGATCAAGTTGTCAAAGAAGATAATATTGAAGCTGTAGGGAAAAAATTACATGAATATAA

ACCTACACTTTAATGAAATAGGTCATAGGTTTATGGTTGTCCTAGTTCAACAGTTTCTTCTATTATAACTTCGACATCCCTTTTTTAATGTACTTATATT

L D V K L L Y P V S K Y Q Q D Q V V K E D N I E A V G K K L H E Y N>

__________________________________TRANSLATION OF MYC-P85ALPHA [A]___________________________________>

1410 1420 1430 1440 1450 1460 1470 1480 1490 1500

CACTCAGTTTCAAGAAAAAAGTCGAGAATATGATAGATTATATGAAGAATATACCCGCACATCCCAGGAAATCCAAATGAAAAGGACAGCTATTGAAGCA

GTGAGTCAAAGTTCTTTTTTCAGCTCTTATACTATCTAATATACTTCTTATATGGGCGTGTAGGGTCCTTTAGGTTTACTTTTCCTGTCGATAACTTCGT

T Q F Q E K S R E Y D R L Y E E Y T R T S Q E I Q M K R T A I E A>

__________________________________TRANSLATION OF MYC-P85ALPHA [A]___________________________________>

1510 1520 1530 1540 1550 1560 1570 1580 1590 1600

TTTAATGAAACCATAAAAATATTTGAAGAACAGTGCCAGACCCAAGAGCGGTACAGCAAAGAATACATAGAAAAGTTTAAACGTGAAGGCAATGAGAAAG

AAATTACTTTGGTATTTTTATAAACTTCTTGTCACGGTCTGGGTTCTCGCCATGTCGTTTCTTATGTATCTTTTCAAATTTGCACTTCCGTTACTCTTTC

F N E T I K I F E E Q C Q T Q E R Y S K E Y I E K F K R E G N E K>

__________________________________TRANSLATION OF MYC-P85ALPHA [A]___________________________________>

1610 1620 1630 1640 1650 1660 1670 1680 1690 1700

AAATACAAAGGATTATGCATAATTATGATAAGTTGAAGTCTCGAATCAGTGAAATTATTGACAGTAGAAGAAGATTGGAAGAAGACTTGAAGAAGCAGGC

TTTATGTTTCCTAATACGTATTAATACTATTCAACTTCAGAGCTTAGTCACTTTAATAACTGTCATCTTCTTCTAACCTTCTTCTGAACTTCTTCGTCCG

E I Q R I M H N Y D K L K S R I S E I I D S R R R L E E D L K K Q A>

__________________________________TRANSLATION OF MYC-P85ALPHA [A]___________________________________>

1710 1720 1730 1740 1750 1760 1770 1780 1790 1800

AGCTGAGTATCGAGAAATTGACAAACGTATGAACAGCATTAAACCAGACCTTATCCAGCTGAGAAAGACGAGAGACCAATACTTGATGTGGTTGACTCAA

TCGACTCATAGCTCTTTAACTGTTTGCATACTTGTCGTAATTTGGTCTGGAATAGGTCGACTCTTTCTGCTCTCTGGTTATGAACTACACCAACTGAGTT

A E Y R E I D K R M N S I K P D L I Q L R K T R D Q Y L M W L T Q>

__________________________________TRANSLATION OF MYC-P85ALPHA [A]___________________________________>

1810 1820 1830 1840 1850 1860 1870 1880 1890 1900

AAAGGTGTTCGGCAAAAGAAGTTGAACGAGTGGTTGGGCAATGAAAACACTGAAGACCAATATTCACTGGTGGAAGATGATGAAGATTTGCCCCATCATG

TTTCCACAAGCCGTTTTCTTCAACTTGCTCACCAACCCGTTACTTTTGTGACTTCTGGTTATAAGTGACCACCTTCTACTACTTCTAAACGGGGTAGTAC

K G V R Q K K L N E W L G N E N T E D Q Y S L V E D D E D L P H H>

__________________________________TRANSLATION OF MYC-P85ALPHA [A]___________________________________>

1910 1920 1930 1940 1950 1960 1970 1980 1990 2000

ATGAGAAGACATGGAATGTTGGAAGCAGCAACCGAAACAAAGCTGAAAACCTGTTGCGAGGGAAGCGAGATGGCACTTTTCTTGTCCGGGAGAGCAGTAA

TACTCTTCTGTACCTTACAACCTTCGTCGTTGGCTTTGTTTCGACTTTTGGACAACGCTCCCTTCGCTCTACCGTGAAAAGAACAGGCCCTCTCGTCATT

D E K T W N V G S S N R N K A E N L L R G K R D G T F L V R E S S K>

__________________________________TRANSLATION OF MYC-P85ALPHA [A]___________________________________>

2010 2020 2030 2040 2050 2060 2070 2080 2090 2100

ACAGGGCTGCTATGCCTGCTCTGTAGTGGTGGACGGCGAAGTAAAGCATTGTGTCATAAACAAAACAGCAACTGGCTATGGCTTTGCCGAGCCCTATAAC

TGTCCCGACGATACGGACGAGACATCACCACCTGCCGCTTCATTTCGTAACACAGTATTTGTTTTGTCGTTGACCGATACCGAAACGGCTCGGGATATTG

Q G C Y A C S V V V D G E V K H C V I N K T A T G Y G F A E P Y N>

__________________________________TRANSLATION OF MYC-P85ALPHA [A]___________________________________>

2110 2120 2130 2140 2150 2160 2170 2180 2190 2200

TTGTACAGCTCTCTGAAAGAACTGGTGCTACATTACCAACACACCTCCCTTGTGCAGCACAACGACTCCCTCAATGTCACACTAGCCTACCCAGTATATG

AACATGTCGAGAGACTTTCTTGACCACGATGTAATGGTTGTGTGGAGGGAACACGTCGTGTTGCTGAGGGAGTTACAGTGTGATCGGATGGGTCATATAC

L Y S S L K E L V L H Y Q H T S L V Q H N D S L N V T L A Y P V Y>

__________________________________TRANSLATION OF MYC-P85ALPHA [A]___________________________________>

2210

CACAGCAGAGGCGATGA

GTGTCGTCTCCGCTACT

A Q Q R R *>

___TRANSLATIO____>

Sequence: PI3Kalpha RBD-GFP2 Range: 1 to 1257

10 20 30 40 50 60 70 80 90 100

ATGAGTAGAGCAATGTATGTTTATCCTCCAAATGTAGAATCTTCACCAGAACTGCCAAAGCACATATATAATAAATTGGATAAAGGGCAAATAATAGTGG

TACTCATCTCGTTACATACAAATAGGAGGTTTACATCTTAGAAGTGGTCTTGACGGTTTCGTGTATATATTATTTAACCTATTTCCCGTTTATTATCACC

M S R A M Y V Y P P N V E S S P E L P K H I Y N K L D K G Q I I V>

_______________________________TRANSLATION OF PI3KALPHA RBD-GFP2 [A]________________________________>

110 120 130 140 150 160 170 180 190 200

TGATTTGGGTAATAGTTTCTCCAAATAATGACAAACAGAAGTATACTCTGAAAATCAACCATGACTGTGTGCCAGAACAAGTAATTGCTGAAGCAATCAG

ACTAAACCCATTATCAAAGAGGTTTATTACTGTTTGTCTTCATATGAGACTTTTAGTTGGTACTGACACACGGTCTTGTTCATTAACGACTTCGTTAGTC

V I W V I V S P N N D K Q K Y T L K I N H D C V P E Q V I A E A I R>

_______________________________TRANSLATION OF PI3KALPHA RBD-GFP2 [A]________________________________>

210 220 230 240 250 260 270 280 290 300

GAAAAAAACTCGAAGTATGTTGCTATCATCTGAACAACTAAAACTCTGTGTTTTAGAATATCAGGGCAAGTATATTTTAAAAGTGTGTGGATGTGATGAA

CTTTTTTTGAGCTTCATACAACGATAGTAGACTTGTTGATTTTGAGACACAAAATCTTATAGTCCCGTTCATATAAAATTTTCACACACCTACACTACTT

K K T R S M L L S S E Q L K L C V L E Y Q G K Y I L K V C G C D E>

_______________________________TRANSLATION OF PI3KALPHA RBD-GFP2 [A]________________________________>

310 320 330 340 350 360 370 380 390 400

TACTTCCTAGAAAAATATCCTCTGAGTCAGTATAAGTATATAAGAAGCTGTATAATGCTTGGGAGGATGCCCAATTTGATGCTGATGGCTAAAGAAAGCC

ATGAAGGATCTTTTTATAGGAGACTCAGTCATATTCATATATTCTTCGACATATTACGAACCCTCCTACGGGTTAAACTACGACTACCGATTTCTTTCGG

Y F L E K Y P L S Q Y K Y I R S C I M L G R M P N L M L M A K E S>

_______________________________TRANSLATION OF PI3KALPHA RBD-GFP2 [A]________________________________>

410 420 430 440 450 460 470 480 490 500

TCTATTCTCAACTGCCAATGGACTGTTTTACAATGCCATCATATTCCAGACGCATTTCCACAGCTACACTCGAGGGCGGCGGAGGATCTGGGGGCGGAGG

AGATAAGAGTTGACGGTTACCTGACAAAATGTTACGGTAGTATAAGGTCTGCGTAAAGGTGTCGATGTGAGCTCCCGCCGCCTCCTAGACCCCCGCCTCC

L Y S Q L P M D C F T M P S Y S R R I S T A T L E G G G G S G G G G>

_______________________________TRANSLATION OF PI3KALPHA RBD-GFP2 [A]________________________________>

510 520 530 540 550 560 570 580 590 600

AAGTGGGGGAGGGGGCTCTGCGGCCGCAGGGAGTGGTATGGTGAGCAAGGGCGAGGAGCTGTTCACCGGGGTGGTGCCCATCCTGGTCGAGCTGGACGGC

TTCACCCCCTCCCCCGAGACGCCGGCGTCCCTCACCATACCACTCGTTCCCGCTCCTCGACAAGTGGCCCCACCACGGGTAGGACCAGCTCGACCTGCCG

S G G G G S A A A G S G M V S K G E E L F T G V V P I L V E L D G>

_______________________________TRANSLATION OF PI3KALPHA RBD-GFP2 [A]________________________________>

610 620 630 640 650 660 670 680 690 700

GACGTAAACGGCCACAAGTTCAGCGTGTCCGGCGAGGGCGAGGGCGATGCCACCTACGGCAAGCTGACCCTGAAGTTCATCTGCACCACCGGCAAGCTGC

CTGCATTTGCCGGTGTTCAAGTCGCACAGGCCGCTCCCGCTCCCGCTACGGTGGATGCCGTTCGACTGGGACTTCAAGTAGACGTGGTGGCCGTTCGACG

D V N G H K F S V S G E G E G D A T Y G K L T L K F I C T T G K L>

_______________________________TRANSLATION OF PI3KALPHA RBD-GFP2 [A]________________________________>

710 720 730 740 750 760 770 780 790 800

CCGTGCCCTGGCCCACCCTCGTGACCACCCTGAGCTACGGCGTGCAGTGCTTCAGCCGCTACCCCGACCACATGAAGCAGCACGACTTCTTCAAGTCCGC

GGCACGGGACCGGGTGGGAGCACTGGTGGGACTCGATGCCGCACGTCACGAAGTCGGCGATGGGGCTGGTGTACTTCGTCGTGCTGAAGAAGTTCAGGCG

P V P W P T L V T T L S Y G V Q C F S R Y P D H M K Q H D F F K S A>

_______________________________TRANSLATION OF PI3KALPHA RBD-GFP2 [A]________________________________>

810 820 830 840 850 860 870 880 890 900

CATGCCCGAAGGCTACGTCCAGGAGCGCACCATCTTCTTCAAGGACGACGGCAACTACAAGACCCGCGCCGAGGTGAAGTTCGAGGGCGACACCCTGGTG

GTACGGGCTTCCGATGCAGGTCCTCGCGTGGTAGAAGAAGTTCCTGCTGCCGTTGATGTTCTGGGCGCGGCTCCACTTCAAGCTCCCGCTGTGGGACCAC

M P E G Y V Q E R T I F F K D D G N Y K T R A E V K F E G D T L V>

_______________________________TRANSLATION OF PI3KALPHA RBD-GFP2 [A]________________________________>

910 920 930 940 950 960 970 980 990 1000

AACCGCATCGAGCTGAAGGGCATCGACTTCAAGGAGGACGGCAACATCCTGGGGCACAAGCTGGAGTACAACTACAACAGCCACAACGTCTATATCATGG

TTGGCGTAGCTCGACTTCCCGTAGCTGAAGTTCCTCCTGCCGTTGTAGGACCCCGTGTTCGACCTCATGTTGATGTTGTCGGTGTTGCAGATATAGTACC

N R I E L K G I D F K E D G N I L G H K L E Y N Y N S H N V Y I M>

_______________________________TRANSLATION OF PI3KALPHA RBD-GFP2 [A]________________________________>

1010 1020 1030 1040 1050 1060 1070 1080 1090 1100

CCGACAAGCAGAAGAACGGCATCAAGGTGAACTTCAAGATCCGCCACAACATCGAGGACGGCAGCGTGCAGCTCGCCGACCACTACCAGCAGAACACCCC

GGCTGTTCGTCTTCTTGCCGTAGTTCCACTTGAAGTTCTAGGCGGTGTTGTAGCTCCTGCCGTCGCACGTCGAGCGGCTGGTGATGGTCGTCTTGTGGGG

A D K Q K N G I K V N F K I R H N I E D G S V Q L A D H Y Q Q N T P>

_______________________________TRANSLATION OF PI3KALPHA RBD-GFP2 [A]________________________________>

1110 1120 1130 1140 1150 1160 1170 1180 1190 1200

CATCGGCGACGGCCCCGTGCTGCTGCCCGACAACCACTACCTGAGCACCCAGTCCGCCCTGAGCAAAGACCCCAACGAGAAGCGCGATCACATGGTCCTG

GTAGCCGCTGCCGGGGCACGACGACGGGCTGTTGGTGATGGACTCGTGGGTCAGGCGGGACTCGTTTCTGGGGTTGCTCTTCGCGCTAGTGTACCAGGAC

I G D G P V L L P D N H Y L S T Q S A L S K D P N E K R D H M V L>

_______________________________TRANSLATION OF PI3KALPHA RBD-GFP2 [A]________________________________>

1210 1220 1230 1240 1250

CTGGAGTTCGTGACCGCCGCCGGGATCACTCTCGGCATGGACGAGCTGTACAAGTAA

GACCTCAAGCACTGGCGGCGGCCCTAGTGAGAGCCGTACCTGCTCGACATGTTCATT

L E F V T A A G I T L G M D E L Y K *>

_________TRANSLATION OF PI3KALPHA RBD-GFP2 [A]___________>

Sequence: PI3Kalpha full-length-GFP2 Range: 1 to 3948

10 20 30 40 50 60 70 80 90 100

ATGCCCCCAAGAATCCTAGTAGAATGTTTACTACCAAATGGAATGATAGTGACTTTAGAATGCCTCCGTGAGGCTACATTAATAACCATAAAGCATGAAC

TACGGGGGTTCTTAGGATCATCTTACAAATGATGGTTTACCTTACTATCACTGAAATCTTACGGAGGCACTCCGATGTAATTATTGGTATTTCGTACTTG

M P P R I L V E C L L P N G M I V T L E C L R E A T L I T I K H E>

___________________________TRANSLATION OF PI3KALPHA FULL-LENGTH-GFP2 [A]____________________________>

110 120 130 140 150 160 170 180 190 200

TATTTAAAGAAGCAAGAAAATACCCCCTCCATCAACTTCTTCAAGATGAATCTTCTTACATTTTCGTAAGTGTTACTCAAGAAGCAGAAAGGGAAGAATT

ATAAATTTCTTCGTTCTTTTATGGGGGAGGTAGTTGAAGAAGTTCTACTTAGAAGAATGTAAAAGCATTCACAATGAGTTCTTCGTCTTTCCCTTCTTAA

L F K E A R K Y P L H Q L L Q D E S S Y I F V S V T Q E A E R E E F>

___________________________TRANSLATION OF PI3KALPHA FULL-LENGTH-GFP2 [A]____________________________>

210 220 230 240 250 260 270 280 290 300

TTTTGATGAAACAAGACGACTTTGTGACCTTCGGCTTTTTCAACCCTTTTTAAAAGTAATTGAACCAGTAGGCAACCGTGAAGAAAAGATCCTCAATCGA

AAAACTACTTTGTTCTGCTGAAACACTGGAAGCCGAAAAAGTTGGGAAAAATTTTCATTAACTTGGTCATCCGTTGGCACTTCTTTTCTAGGAGTTAGCT

F D E T R R L C D L R L F Q P F L K V I E P V G N R E E K I L N R>

___________________________TRANSLATION OF PI3KALPHA FULL-LENGTH-GFP2 [A]____________________________>

310 320 330 340 350 360 370 380 390 400

GAAATTGGTTTTGCTATCGGCATGCCAGTGTGTGAATTTGATATGGTTAAAGATCCAGAAGTACAGGACTTCCGAAGAAATATTCTGAACGTTTGTAAAG

CTTTAACCAAAACGATAGCCGTACGGTCACACACTTAAACTATACCAATTTCTAGGTCTTCATGTCCTGAAGGCTTCTTTATAAGACTTGCAAACATTTC

E I G F A I G M P V C E F D M V K D P E V Q D F R R N I L N V C K>

___________________________TRANSLATION OF PI3KALPHA FULL-LENGTH-GFP2 [A]____________________________>

410 420 430 440 450 460 470 480 490 500

AAGCTGTGGATCTTAGGGACCTCAATTCACCTCATAGTAGAGCAATGTATGTCTATCCTCCAAATGTAGAATCTTCACCAGAATTGCCAAAGCACATATA

TTCGACACCTAGAATCCCTGGAGTTAAGTGGAGTATCATCTCGTTACATACAGATAGGAGGTTTACATCTTAGAAGTGGTCTTAACGGTTTCGTGTATAT

E A V D L R D L N S P H S R A M Y V Y P P N V E S S P E L P K H I Y>

___________________________TRANSLATION OF PI3KALPHA FULL-LENGTH-GFP2 [A]____________________________>

510 520 530 540 550 560 570 580 590 600

TAATAAATTAGATAAAGGGCAAATAATAGTGGTGATCTGGGTAATAGTTTCTCCAAATAATGACAAGCAGAAGTATACTCTGAAAATCAACCATGACTGT

ATTATTTAATCTATTTCCCGTTTATTATCACCACTAGACCCATTATCAAAGAGGTTTATTACTGTTCGTCTTCATATGAGACTTTTAGTTGGTACTGACA

N K L D K G Q I I V V I W V I V S P N N D K Q K Y T L K I N H D C>

___________________________TRANSLATION OF PI3KALPHA FULL-LENGTH-GFP2 [A]____________________________>

610 620 630 640 650 660 670 680 690 700

GTACCAGAACAAGTAATTGCTGAAGCAATCAGGAAAAAAACTCGAAGTATGTTGCTATCCTCTGAACAACTAAAACTCTGTGTTTTAGAATATCAGGGCA

CATGGTCTTGTTCATTAACGACTTCGTTAGTCCTTTTTTTGAGCTTCATACAACGATAGGAGACTTGTTGATTTTGAGACACAAAATCTTATAGTCCCGT

V P E Q V I A E A I R K K T R S M L L S S E Q L K L C V L E Y Q G>

___________________________TRANSLATION OF PI3KALPHA FULL-LENGTH-GFP2 [A]____________________________>

710 720 730 740 750 760 770 780 790 800

AGTATATTTTAAAAGTGTGTGGATGTGATGAATACTTCCTAGAAAAATATCCTCTGAGTCAGTATAAGTATATAAGAAGCTGTATAATGCTTGGGAGGAT

TCATATAAAATTTTCACACACCTACACTACTTATGAAGGATCTTTTTATAGGAGACTCAGTCATATTCATATATTCTTCGACATATTACGAACCCTCCTA

K Y I L K V C G C D E Y F L E K Y P L S Q Y K Y I R S C I M L G R M>

___________________________TRANSLATION OF PI3KALPHA FULL-LENGTH-GFP2 [A]____________________________>

810 820 830 840 850 860 870 880 890 900

GCCCAATTTGATGTTGATGGCTAAAGAAAGCCTTTATTCTCAACTGCCAATGGACTGTTTTACAATGCCATCTTATTCCAGACGCATTTCCACAGCTACA

CGGGTTAAACTACAACTACCGATTTCTTTCGGAAATAAGAGTTGACGGTTACCTGACAAAATGTTACGGTAGAATAAGGTCTGCGTAAAGGTGTCGATGT

P N L M L M A K E S L Y S Q L P M D C F T M P S Y S R R I S T A T>

___________________________TRANSLATION OF PI3KALPHA FULL-LENGTH-GFP2 [A]____________________________>

910 920 930 940 950 960 970 980 990 1000

CCATATATGAATGGAGAAACATCTACAAAATCCCTTTGGGTTATAAATAGTGCACTCAGAATAAAAATTCTTTGTGCAACCTACGTGAATGTAAATATTC

GGTATATACTTACCTCTTTGTAGATGTTTTAGGGAAACCCAATATTTATCACGTGAGTCTTATTTTTAAGAAACACGTTGGATGCACTTACATTTATAAG

P Y M N G E T S T K S L W V I N S A L R I K I L C A T Y V N V N I>

___________________________TRANSLATION OF PI3KALPHA FULL-LENGTH-GFP2 [A]____________________________>

1010 1020 1030 1040 1050 1060 1070 1080 1090 1100

GAGACATTGATAAGATCTATGTTCGAACAGGTATCTACCATGGAGGAGAACCCTTATGTGACAATGTGAACACTCAAAGAGTACCTTGTTCCAATCCCAG

CTCTGTAACTATTCTAGATACAAGCTTGTCCATAGATGGTACCTCCTCTTGGGAATACACTGTTACACTTGTGAGTTTCTCATGGAACAAGGTTAGGGTC

R D I D K I Y V R T G I Y H G G E P L C D N V N T Q R V P C S N P R>

___________________________TRANSLATION OF PI3KALPHA FULL-LENGTH-GFP2 [A]____________________________>

1110 1120 1130 1140 1150 1160 1170 1180 1190 1200

GTGGAATGAATGGCTGAATTATGATATATACATTCCTGATCTTCCTCGTGCTGCTCGACTTTGCCTTTCCATTTGCTCTGTTAAAGGCCGAAAGGGTGCT

CACCTTACTTACCGACTTAATACTATATATGTAAGGACTAGAAGGAGCACGACGAGCTGAAACGGAAAGGTAAACGAGACAATTTCCGGCTTTCCCACGA

W N E W L N Y D I Y I P D L P R A A R L C L S I C S V K G R K G A>

___________________________TRANSLATION OF PI3KALPHA FULL-LENGTH-GFP2 [A]____________________________>

1210 1220 1230 1240 1250 1260 1270 1280 1290 1300

AAAGAGGAACACTGTCCATTGGCATGGGGAAATATAAACTTGTTTGATTACACAGACACTCTAGTATCTGGAAAAATGGCTTTGAATCTTTGGCCAGTAC

TTTCTCCTTGTGACAGGTAACCGTACCCCTTTATATTTGAACAAACTAATGTGTCTGTGAGATCATAGACCTTTTTACCGAAACTTAGAAACCGGTCATG

K E E H C P L A W G N I N L F D Y T D T L V S G K M A L N L W P V>

___________________________TRANSLATION OF PI3KALPHA FULL-LENGTH-GFP2 [A]____________________________>

1310 1320 1330 1340 1350 1360 1370 1380 1390 1400

CTCATGGATTAGAAGATTTGCTGAACCCTATTGGTGTTACTGGATCAAATCCAAATAAAGAAACTCCATGCTTAGAGTTGGAGTTTGACTGGTTCAGCAG

GAGTACCTAATCTTCTAAACGACTTGGGATAACCACAATGACCTAGTTTAGGTTTATTTCTTTGAGGTACGAATCTCAACCTCAAACTGACCAAGTCGTC

P H G L E D L L N P I G V T G S N P N K E T P C L E L E F D W F S S>

___________________________TRANSLATION OF PI3KALPHA FULL-LENGTH-GFP2 [A]____________________________>

1410 1420 1430 1440 1450 1460 1470 1480 1490 1500

TGTGGTAAAGTTCCCAGATATGTCAGTGATTGAAGAGCATGCCAATTGGTCTGTATCCCGAGAAGCAGGATTTAGCTATTCCCACGCAGGACTGAGTAAC

ACACCATTTCAAGGGTCTATACAGTCACTAACTTCTCGTACGGTTAACCAGACATAGGGCTCTTCGTCCTAAATCGATAAGGGTGCGTCCTGACTCATTG

V V K F P D M S V I E E H A N W S V S R E A G F S Y S H A G L S N>

___________________________TRANSLATION OF PI3KALPHA FULL-LENGTH-GFP2 [A]____________________________>

1510 1520 1530 1540 1550 1560 1570 1580 1590 1600

AGACTAGCTAGAGACAATGAATTAAGGGAAAATGACAAAGAACAGCTCAAAGCAATTTCTACACGAGATCCTCTCTCTGAAATCACTGAGCAGGAGAAAG

TCTGATCGATCTCTGTTACTTAATTCCCTTTTACTGTTTCTTGTCGAGTTTCGTTAAAGATGTGCTCTAGGAGAGAGACTTTAGTGACTCGTCCTCTTTC

R L A R D N E L R E N D K E Q L K A I S T R D P L S E I T E Q E K>

___________________________TRANSLATION OF PI3KALPHA FULL-LENGTH-GFP2 [A]____________________________>

1610 1620 1630 1640 1650 1660 1670 1680 1690 1700

ATTTTCTATGGAGTCACAGACACTATTGTGTAACTATCCCCGAAATTCTACCCAAATTGCTTCTGTCTGTTAAATGGAATTCTAGAGATGAAGTAGCCCA

TAAAAGATACCTCAGTGTCTGTGATAACACATTGATAGGGGCTTTAAGATGGGTTTAACGAAGACAGACAATTTACCTTAAGATCTCTACTTCATCGGGT

D F L W S H R H Y C V T I P E I L P K L L L S V K W N S R D E V A Q>

___________________________TRANSLATION OF PI3KALPHA FULL-LENGTH-GFP2 [A]____________________________>

1710 1720 1730 1740 1750 1760 1770 1780 1790 1800

GATGTATTGCTTGGTAAAAGATTGGCCTCCAATCAAACCTGAACAGGCTATGGAACTTCTGGACTGTAATTACCCAGATCCTATGGTTCGAGGTTTTGCT

CTACATAACGAACCATTTTCTAACCGGAGGTTAGTTTGGACTTGTCCGATACCTTGAAGACCTGACATTAATGGGTCTAGGATACCAAGCTCCAAAACGA

M Y C L V K D W P P I K P E Q A M E L L D C N Y P D P M V R G F A>

___________________________TRANSLATION OF PI3KALPHA FULL-LENGTH-GFP2 [A]____________________________>

1810 1820 1830 1840 1850 1860 1870 1880 1890 1900

GTTCGGTGCTTGGAAAAATATTTAACAGATGACAAACTTTCTCAGTATTTAATTCAGCTAGTACAGGTCCTAAAATATGAACAATATTTGGATAACTTGC

CAAGCCACGAACCTTTTTATAAATTGTCTACTGTTTGAAAGAGTCATAAATTAAGTCGATCATGTCCAGGATTTTATACTTGTTATAAACCTATTGAACG

V R C L E K Y L T D D K L S Q Y L I Q L V Q V L K Y E Q Y L D N L>

___________________________TRANSLATION OF PI3KALPHA FULL-LENGTH-GFP2 [A]____________________________>

1910 1920 1930 1940 1950 1960 1970 1980 1990 2000

TTGTGAGATTTTTACTGAAGAAAGCATTGACTAATCAAAGGATTGGGCACTTTTTCTTTTGGCATTTAAAATCTGAGATGCACAATAAAACAGTTAGCCA

AACACTCTAAAAATGACTTCTTTCGTAACTGATTAGTTTCCTAACCCGTGAAAAAGAAAACCGTAAATTTTAGACTCTACGTGTTATTTTGTCAATCGGT

L V R F L L K K A L T N Q R I G H F F F W H L K S E M H N K T V S Q>

___________________________TRANSLATION OF PI3KALPHA FULL-LENGTH-GFP2 [A]____________________________>

2010 2020 2030 2040 2050 2060 2070 2080 2090 2100

GAGGTTTGGCCTGCTTTTGGAGTCCTATTGTCGTGCATGTGGGATGTATTTGAAGCACCTGAATAGGCAAGTCGAGGCAATGGAAAAGCTCATTAACTTA

CTCCAAACCGGACGAAAACCTCAGGATAACAGCACGTACACCCTACATAAACTTCGTGGACTTATCCGTTCAGCTCCGTTACCTTTTCGAGTAATTGAAT

R F G L L L E S Y C R A C G M Y L K H L N R Q V E A M E K L I N L>

___________________________TRANSLATION OF PI3KALPHA FULL-LENGTH-GFP2 [A]____________________________>

2110 2120 2130 2140 2150 2160 2170 2180 2190 2200

ACTGACATTCTCAAACAGGAGAAGAAGGATGAAACACAAAAGGTACAGATGAAGTTTTTAGTTGAGCAAATGAGGCGACCAGATTTCATGGATGCTCTAC

TGACTGTAAGAGTTTGTCCTCTTCTTCCTACTTTGTGTTTTCCATGTCTACTTCAAAAATCAACTCGTTTACTCCGCTGGTCTAAAGTACCTACGAGATG

T D I L K Q E K K D E T Q K V Q M K F L V E Q M R R P D F M D A L>

___________________________TRANSLATION OF PI3KALPHA FULL-LENGTH-GFP2 [A]____________________________>

2210 2220 2230 2240 2250 2260 2270 2280 2290 2300

AGGGCTTTCTGTCTCCTCTAAACCCTGCTCATCAACTAGGAAACCTCAGGCTTGAAGAGTGTCGAATTATGTCCTCTGCAAAAAGGCCACTGTGGTTGAA

TCCCGAAAGACAGAGGAGATTTGGGACGAGTAGTTGATCCTTTGGAGTCCGAACTTCTCACAGCTTAATACAGGAGACGTTTTTCCGGTGACACCAACTT

Q G F L S P L N P A H Q L G N L R L E E C R I M S S A K R P L W L N>

___________________________TRANSLATION OF PI3KALPHA FULL-LENGTH-GFP2 [A]____________________________>

2310 2320 2330 2340 2350 2360 2370 2380 2390 2400

TTGGGAGAACCCAGACATCATGTCAGAGTTACTGTTTCAGAACAATGAGATCATCTTTAAAAATGGGGATGATTTACGGCAAGATATGCTAACACTTCAA

AACCCTCTTGGGTCTGTAGTACAGTCTCAATGACAAAGTCTTGTTACTCTAGTAGAAATTTTTACCCCTACTAAATGCCGTTCTATACGATTGTGAAGTT

W E N P D I M S E L L F Q N N E I I F K N G D D L R Q D M L T L Q>

___________________________TRANSLATION OF PI3KALPHA FULL-LENGTH-GFP2 [A]____________________________>

2410 2420 2430 2440 2450 2460 2470 2480 2490 2500

ATTATTCGTATTATGGAAAATATCTGGCAAAATCAAGGTCTTGATCTTCGAATGTTACCTTATGGTTGTCTGTCAATCGGTGACTGTGTGGGACTTATTG

TAATAAGCATAATACCTTTTATAGACCGTTTTAGTTCCAGAACTAGAAGCTTACAATGGAATACCAACAGACAGTTAGCCACTGACACACCCTGAATAAC

I I R I M E N I W Q N Q G L D L R M L P Y G C L S I G D C V G L I>

___________________________TRANSLATION OF PI3KALPHA FULL-LENGTH-GFP2 [A]____________________________>

2510 2520 2530 2540 2550 2560 2570 2580 2590 2600

AGGTGGTGCGAAATTCTCACACTATTATGCAAATTCAGTGCAAAGGCGGCTTGAAAGGTGCACTGCAGTTCAACAGCCACACACTACATCAGTGGCTCAA

TCCACCACGCTTTAAGAGTGTGATAATACGTTTAAGTCACGTTTCCGCCGAACTTTCCACGTGACGTCAAGTTGTCGGTGTGTGATGTAGTCACCGAGTT

E V V R N S H T I M Q I Q C K G G L K G A L Q F N S H T L H Q W L K>

___________________________TRANSLATION OF PI3KALPHA FULL-LENGTH-GFP2 [A]____________________________>

2610 2620 2630 2640 2650 2660 2670 2680 2690 2700

AGACAAGAACAAAGGAGAAATATATGATGCAGCCATTGACCTGTTTACACGTTCATGTGCTGGATACTGTGTAGCTACCTTCATTTTGGGAATTGGAGAT

TCTGTTCTTGTTTCCTCTTTATATACTACGTCGGTAACTGGACAAATGTGCAAGTACACGACCTATGACACATCGATGGAAGTAAAACCCTTAACCTCTA

D K N K G E I Y D A A I D L F T R S C A G Y C V A T F I L G I G D>

___________________________TRANSLATION OF PI3KALPHA FULL-LENGTH-GFP2 [A]____________________________>

2710 2720 2730 2740 2750 2760 2770 2780 2790 2800

CGTCACAATAGTAACATCATGGTGAAAGACGATGGACAACTGTTTCATATAGATTTTGGACACTTTTTGGATCACAAGAAGAAAAAATTTGGTTATAAAC

GCAGTGTTATCATTGTAGTACCACTTTCTGCTACCTGTTGACAAAGTATATCTAAAACCTGTGAAAAACCTAGTGTTCTTCTTTTTTAAACCAATATTTG

R H N S N I M V K D D G Q L F H I D F G H F L D H K K K K F G Y K>

___________________________TRANSLATION OF PI3KALPHA FULL-LENGTH-GFP2 [A]____________________________>

2810 2820 2830 2840 2850 2860 2870 2880 2890 2900

GAGAACGTGTGCCATTTGTTTTGACACAGGATTTCTTAATAGTGATTAGTAAAGGAGCCCAAGAATGCACAAAGACAAGAGAATTTGAGAGGTTTCAGGA

CTCTTGCACACGGTAAACAAAACTGTGTCCTAAAGAATTATCACTAATCATTTCCTCGGGTTCTTACGTGTTTCTGTTCTCTTAAACTCTCCAAAGTCCT

R E R V P F V L T Q D F L I V I S K G A Q E C T K T R E F E R F Q E>

___________________________TRANSLATION OF PI3KALPHA FULL-LENGTH-GFP2 [A]____________________________>

2910 2920 2930 2940 2950 2960 2970 2980 2990 3000

GATGTGTTACAAGGCTTATCTAGCTATTCGACAGCATGCCAATCTCTTCATAAATCTTTTCTCAATGATGCTTGGCTCTGGAATGCCAGAACTACAATCT

CTACACAATGTTCCGAATAGATCGATAAGCTGTCGTACGGTTAGAGAAGTATTTAGAAAAGAGTTACTACGAACCGAGACCTTACGGTCTTGATGTTAGA

M C Y K A Y L A I R Q H A N L F I N L F S M M L G S G M P E L Q S>

___________________________TRANSLATION OF PI3KALPHA FULL-LENGTH-GFP2 [A]____________________________>

3010 3020 3030 3040 3050 3060 3070 3080 3090 3100

TTTGATGACATTGCATACATTCGAAAGACCCTAGCCTTAGATAAAACTGAGCAAGAGGCTTTGGAGTATTTCATGAAACAAATGAATGATGCACATCATG

AAACTACTGTAACGTATGTAAGCTTTCTGGGATCGGAATCTATTTTGACTCGTTCTCCGAAACCTCATAAAGTACTTTGTTTACTTACTACGTGTAGTAC

F D D I A Y I R K T L A L D K T E Q E A L E Y F M K Q M N D A H H>

___________________________TRANSLATION OF PI3KALPHA FULL-LENGTH-GFP2 [A]____________________________>

3110 3120 3130 3140 3150 3160 3170 3180 3190 3200

GTGGCTGGACAACAAAAATGGATTGGATCTTCCACACAATTAAACAGCATGCATTGAACCTCGAGGGCGGCGGAGGATCTGGGGGCGGAGGAAGTGGGGG

CACCGACCTGTTGTTTTTACCTAACCTAGAAGGTGTGTTAATTTGTCGTACGTAACTTGGAGCTCCCGCCGCCTCCTAGACCCCCGCCTCCTTCACCCCC

G G W T T K M D W I F H T I K Q H A L N L E G G G G S G G G G S G G>

___________________________TRANSLATION OF PI3KALPHA FULL-LENGTH-GFP2 [A]____________________________>

3210 3220 3230 3240 3250 3260 3270 3280 3290 3300

AGGGGGCTCTGCGGCCGCAGGGAGTGGTATGGTGAGCAAGGGCGAGGAGCTGTTCACCGGGGTGGTGCCCATCCTGGTCGAGCTGGACGGCGACGTAAAC

TCCCCCGAGACGCCGGCGTCCCTCACCATACCACTCGTTCCCGCTCCTCGACAAGTGGCCCCACCACGGGTAGGACCAGCTCGACCTGCCGCTGCATTTG

G G S A A A G S G M V S K G E E L F T G V V P I L V E L D G D V N>

___________________________TRANSLATION OF PI3KALPHA FULL-LENGTH-GFP2 [A]____________________________>

3310 3320 3330 3340 3350 3360 3370 3380 3390 3400

GGCCACAAGTTCAGCGTGTCCGGCGAGGGCGAGGGCGATGCCACCTACGGCAAGCTGACCCTGAAGTTCATCTGCACCACCGGCAAGCTGCCCGTGCCCT

CCGGTGTTCAAGTCGCACAGGCCGCTCCCGCTCCCGCTACGGTGGATGCCGTTCGACTGGGACTTCAAGTAGACGTGGTGGCCGTTCGACGGGCACGGGA

G H K F S V S G E G E G D A T Y G K L T L K F I C T T G K L P V P>

___________________________TRANSLATION OF PI3KALPHA FULL-LENGTH-GFP2 [A]____________________________>

3410 3420 3430 3440 3450 3460 3470 3480 3490 3500

GGCCCACCCTCGTGACCACCCTGAGCTACGGCGTGCAGTGCTTCAGCCGCTACCCCGACCACATGAAGCAGCACGACTTCTTCAAGTCCGCCATGCCCGA

CCGGGTGGGAGCACTGGTGGGACTCGATGCCGCACGTCACGAAGTCGGCGATGGGGCTGGTGTACTTCGTCGTGCTGAAGAAGTTCAGGCGGTACGGGCT

W P T L V T T L S Y G V Q C F S R Y P D H M K Q H D F F K S A M P E>

___________________________TRANSLATION OF PI3KALPHA FULL-LENGTH-GFP2 [A]____________________________>

3510 3520 3530 3540 3550 3560 3570 3580 3590 3600

AGGCTACGTCCAGGAGCGCACCATCTTCTTCAAGGACGACGGCAACTACAAGACCCGCGCCGAGGTGAAGTTCGAGGGCGACACCCTGGTGAACCGCATC

TCCGATGCAGGTCCTCGCGTGGTAGAAGAAGTTCCTGCTGCCGTTGATGTTCTGGGCGCGGCTCCACTTCAAGCTCCCGCTGTGGGACCACTTGGCGTAG

G Y V Q E R T I F F K D D G N Y K T R A E V K F E G D T L V N R I>

___________________________TRANSLATION OF PI3KALPHA FULL-LENGTH-GFP2 [A]____________________________>

3610 3620 3630 3640 3650 3660 3670 3680 3690 3700

GAGCTGAAGGGCATCGACTTCAAGGAGGACGGCAACATCCTGGGGCACAAGCTGGAGTACAACTACAACAGCCACAACGTCTATATCATGGCCGACAAGC

CTCGACTTCCCGTAGCTGAAGTTCCTCCTGCCGTTGTAGGACCCCGTGTTCGACCTCATGTTGATGTTGTCGGTGTTGCAGATATAGTACCGGCTGTTCG

E L K G I D F K E D G N I L G H K L E Y N Y N S H N V Y I M A D K>

___________________________TRANSLATION OF PI3KALPHA FULL-LENGTH-GFP2 [A]____________________________>

3710 3720 3730 3740 3750 3760 3770 3780 3790 3800

AGAAGAACGGCATCAAGGTGAACTTCAAGATCCGCCACAACATCGAGGACGGCAGCGTGCAGCTCGCCGACCACTACCAGCAGAACACCCCCATCGGCGA

TCTTCTTGCCGTAGTTCCACTTGAAGTTCTAGGCGGTGTTGTAGCTCCTGCCGTCGCACGTCGAGCGGCTGGTGATGGTCGTCTTGTGGGGGTAGCCGCT

Q K N G I K V N F K I R H N I E D G S V Q L A D H Y Q Q N T P I G D>

___________________________TRANSLATION OF PI3KALPHA FULL-LENGTH-GFP2 [A]____________________________>

3810 3820 3830 3840 3850 3860 3870 3880 3890 3900

CGGCCCCGTGCTGCTGCCCGACAACCACTACCTGAGCACCCAGTCCGCCCTGAGCAAAGACCCCAACGAGAAGCGCGATCACATGGTCCTGCTGGAGTTC

GCCGGGGCACGACGACGGGCTGTTGGTGATGGACTCGTGGGTCAGGCGGGACTCGTTTCTGGGGTTGCTCTTCGCGCTAGTGTACCAGGACGACCTCAAG

G P V L L P D N H Y L S T Q S A L S K D P N E K R D H M V L L E F>

___________________________TRANSLATION OF PI3KALPHA FULL-LENGTH-GFP2 [A]____________________________>

3910 3920 3930 3940

GTGACCGCCGCCGGGATCACTCTCGGCATGGACGAGCTGTACAAGTAA

CACTGGCGGCGGCCCTAGTGAGAGCCGTACCTGCTCGACATGTTCATT

V T A A G I T L G M D E L Y K *>

___TRANSLATION OF PI3KALPHA FULL-LENGTH-GFP2____>

Sequence: PI3Kgamma RBD-GFP2 Range: 1 to 1167

10 20 30 40 50 60 70 80 90 100

ATGAGCCGCGACCCCAAGCTCTACGCCATGCACCCGTGGGTGACGTCCAAGCCCCTCCCGGAGTACCTGTGGAAGAAGATTGCCAACAACTGCATCTTCA

TACTCGGCGCTGGGGTTCGAGATGCGGTACGTGGGCACCCACTGCAGGTTCGGGGAGGGCCTCATGGACACCTTCTTCTAACGGTTGTTGACGTAGAAGT

M S R D P K L Y A M H P W V T S K P L P E Y L W K K I A N N C I F>

_______________________________TRANSLATION OF PI3KGAMMA RBD-GFP2 [A]________________________________>

110 120 130 140 150 160 170 180 190 200

TCGTCATTCACCGCAGCACCACCAGCCAGACCATTAAGGTCTCACCCGACGACACCCCCGGCGCCATCCTGCAGAGCTTCTTCACCAAGATGGCCAAGAA

AGCAGTAAGTGGCGTCGTGGTGGTCGGTCTGGTAATTCCAGAGTGGGCTGCTGTGGGGGCCGCGGTAGGACGTCTCGAAGAAGTGGTTCTACCGGTTCTT

I V I H R S T T S Q T I K V S P D D T P G A I L Q S F F T K M A K K>

_______________________________TRANSLATION OF PI3KGAMMA RBD-GFP2 [A]________________________________>

210 220 230 240 250 260 270 280 290 300

GAAATCTCTGATGGATATTCCCGAAAGCCAAAGCGAACAGGATTTTGTGCTGCGCGTCTGTGGCCGGGATGAGTACCTGGTGGGCGAAACGCCCATCAAA

CTTTAGAGACTACCTATAAGGGCTTTCGGTTTCGCTTGTCCTAAAACACGACGCGCAGACACCGGCCCTACTCATGGACCACCCGCTTTGCGGGTAGTTT

K S L M D I P E S Q S E Q D F V L R V C G R D E Y L V G E T P I K>

_______________________________TRANSLATION OF PI3KGAMMA RBD-GFP2 [A]________________________________>

310 320 330 340 350 360 370 380 390 400

AACTTCCAGTGGGTGAGGCACTGCCTCAAGAACGGAGAAGAGATTCACGTGGTACTGGACACGCCTCCAGACCCGGCCCTCGAGGGCGGCGGAGGATCTG

TTGAAGGTCACCCACTCCGTGACGGAGTTCTTGCCTCTTCTCTAAGTGCACCATGACCTGTGCGGAGGTCTGGGCCGGGAGCTCCCGCCGCCTCCTAGAC

N F Q W V R H C L K N G E E I H V V L D T P P D P A L E G G G G S>

_______________________________TRANSLATION OF PI3KGAMMA RBD-GFP2 [A]________________________________>

410 420 430 440 450 460 470 480 490 500

GGGGCGGAGGAAGTGGGGGAGGGGGCTCTGCGGCCGCAGGGAGTGGTATGGTGAGCAAGGGCGAGGAGCTGTTCACCGGGGTGGTGCCCATCCTGGTCGA

CCCCGCCTCCTTCACCCCCTCCCCCGAGACGCCGGCGTCCCTCACCATACCACTCGTTCCCGCTCCTCGACAAGTGGCCCCACCACGGGTAGGACCAGCT

G G G G S G G G G S A A A G S G M V S K G E E L F T G V V P I L V E>

_______________________________TRANSLATION OF PI3KGAMMA RBD-GFP2 [A]________________________________>

510 520 530 540 550 560 570 580 590 600

GCTGGACGGCGACGTAAACGGCCACAAGTTCAGCGTGTCCGGCGAGGGCGAGGGCGATGCCACCTACGGCAAGCTGACCCTGAAGTTCATCTGCACCACC

CGACCTGCCGCTGCATTTGCCGGTGTTCAAGTCGCACAGGCCGCTCCCGCTCCCGCTACGGTGGATGCCGTTCGACTGGGACTTCAAGTAGACGTGGTGG

L D G D V N G H K F S V S G E G E G D A T Y G K L T L K F I C T T>

_______________________________TRANSLATION OF PI3KGAMMA RBD-GFP2 [A]________________________________>

610 620 630 640 650 660 670 680 690 700

GGCAAGCTGCCCGTGCCCTGGCCCACCCTCGTGACCACCCTGAGCTACGGCGTGCAGTGCTTCAGCCGCTACCCCGACCACATGAAGCAGCACGACTTCT

CCGTTCGACGGGCACGGGACCGGGTGGGAGCACTGGTGGGACTCGATGCCGCACGTCACGAAGTCGGCGATGGGGCTGGTGTACTTCGTCGTGCTGAAGA

G K L P V P W P T L V T T L S Y G V Q C F S R Y P D H M K Q H D F>

_______________________________TRANSLATION OF PI3KGAMMA RBD-GFP2 [A]________________________________>

710 720 730 740 750 760 770 780 790 800

TCAAGTCCGCCATGCCCGAAGGCTACGTCCAGGAGCGCACCATCTTCTTCAAGGACGACGGCAACTACAAGACCCGCGCCGAGGTGAAGTTCGAGGGCGA

AGTTCAGGCGGTACGGGCTTCCGATGCAGGTCCTCGCGTGGTAGAAGAAGTTCCTGCTGCCGTTGATGTTCTGGGCGCGGCTCCACTTCAAGCTCCCGCT

F K S A M P E G Y V Q E R T I F F K D D G N Y K T R A E V K F E G D>

_______________________________TRANSLATION OF PI3KGAMMA RBD-GFP2 [A]________________________________>

810 820 830 840 850 860 870 880 890 900

CACCCTGGTGAACCGCATCGAGCTGAAGGGCATCGACTTCAAGGAGGACGGCAACATCCTGGGGCACAAGCTGGAGTACAACTACAACAGCCACAACGTC

GTGGGACCACTTGGCGTAGCTCGACTTCCCGTAGCTGAAGTTCCTCCTGCCGTTGTAGGACCCCGTGTTCGACCTCATGTTGATGTTGTCGGTGTTGCAG

T L V N R I E L K G I D F K E D G N I L G H K L E Y N Y N S H N V>

_______________________________TRANSLATION OF PI3KGAMMA RBD-GFP2 [A]________________________________>

910 920 930 940 950 960 970 980 990 1000

TATATCATGGCCGACAAGCAGAAGAACGGCATCAAGGTGAACTTCAAGATCCGCCACAACATCGAGGACGGCAGCGTGCAGCTCGCCGACCACTACCAGC

ATATAGTACCGGCTGTTCGTCTTCTTGCCGTAGTTCCACTTGAAGTTCTAGGCGGTGTTGTAGCTCCTGCCGTCGCACGTCGAGCGGCTGGTGATGGTCG

Y I M A D K Q K N G I K V N F K I R H N I E D G S V Q L A D H Y Q>

_______________________________TRANSLATION OF PI3KGAMMA RBD-GFP2 [A]________________________________>

1010 1020 1030 1040 1050 1060 1070 1080 1090 1100

AGAACACCCCCATCGGCGACGGCCCCGTGCTGCTGCCCGACAACCACTACCTGAGCACCCAGTCCGCCCTGAGCAAAGACCCCAACGAGAAGCGCGATCA

TCTTGTGGGGGTAGCCGCTGCCGGGGCACGACGACGGGCTGTTGGTGATGGACTCGTGGGTCAGGCGGGACTCGTTTCTGGGGTTGCTCTTCGCGCTAGT

Q N T P I G D G P V L L P D N H Y L S T Q S A L S K D P N E K R D H>

_______________________________TRANSLATION OF PI3KGAMMA RBD-GFP2 [A]________________________________>

1110 1120 1130 1140 1150 1160

CATGGTCCTGCTGGAGTTCGTGACCGCCGCCGGGATCACTCTCGGCATGGACGAGCTGTACAAGTAA

GTACCAGGACGACCTCAAGCACTGGCGGCGGCCCTAGTGAGAGCCGTACCTGCTCGACATGTTCATT

M V L L E F V T A A G I T L G M D E L Y K *>

______________TRANSLATION OF PI3KGAMMA RBD-GFP2 [A]________________>

Sequence: CRAF RBD-GFP2 Range: 1 to 1245

10 20 30 40 50 60 70 80 90 100

ATGGAGCACATACAGGGAGCTTGGAAGACGATCAGCAATGGTTTTGGATTCAAAGATGCCGTGTTTGATGGCTCCAGCTGCATCTCTCCTACAATAGTTC

TACCTCGTGTATGTCCCTCGAACCTTCTGCTAGTCGTTACCAAAACCTAAGTTTCTACGGCACAAACTACCGAGGTCGACGTAGAGAGGATGTTATCAAG

M E H I Q G A W K T I S N G F G F K D A V F D G S S C I S P T I V>

_________________________________TRANSLATION OF CRAF RBD-GFP2 [A]___________________________________>

110 120 130 140 150 160 170 180 190 200

AGCAGTTTGGCTATCAGCGCCGGGCATCAGATGATGGCAAACTCACAGATCCTTCTAAGACAAGCAACACTATCCGTGTTTTCTTGCCGAACAAGCAAAG

TCGTCAAACCGATAGTCGCGGCCCGTAGTCTACTACCGTTTGAGTGTCTAGGAAGATTCTGTTCGTTGTGATAGGCACAAAAGAACGGCTTGTTCGTTTC

Q Q F G Y Q R R A S D D G K L T D P S K T S N T I R V F L P N K Q R>

_________________________________TRANSLATION OF CRAF RBD-GFP2 [A]___________________________________>

210 220 230 240 250 260 270 280 290 300

AACAGTGGTCAATGTGCGAAATGGAATGAGCTTGCATGACTGCCTTATGAAAGCACTCAAGGTGAGGGGCCTGCAACCAGAGTGCTGTGCAGTGTTCAGA

TTGTCACCAGTTACACGCTTTACCTTACTCGAACGTACTGACGGAATACTTTCGTGAGTTCCACTCCCCGGACGTTGGTCTCACGACACGTCACAAGTCT

T V V N V R N G M S L H D C L M K A L K V R G L Q P E C C A V F R>

_________________________________TRANSLATION OF CRAF RBD-GFP2 [A]___________________________________>

310 320 330 340 350 360 370 380 390 400

CTTCTCCACGAACACAAAGGTAAAAAAGCACGCTTAGATTGGAATACTGATGCTGCGTCTTTGATTGGAGAAGAACTTCAAGTAGATTTCCTGGATCATG

GAAGAGGTGCTTGTGTTTCCATTTTTTCGTGCGAATCTAACCTTATGACTACGACGCAGAAACTAACCTCTTCTTGAAGTTCATCTAAAGGACCTAGTAC

L L H E H K G K K A R L D W N T D A A S L I G E E L Q V D F L D H>

_________________________________TRANSLATION OF CRAF RBD-GFP2 [A]___________________________________>

410 420 430 440 450 460 470 480 490 500

TTCCCCTCACAACACACAACTTTGCTCGGAAGACGTTCCTGAAGCTTAATTCATCGCTCGAGGGCGGCGGAGGATCTGGGGGCGGAGGAAGTGGGGGAGG

AAGGGGAGTGTTGTGTGTTGAAACGAGCCTTCTGCAAGGACTTCGAATTAAGTAGCGAGCTCCCGCCGCCTCCTAGACCCCCGCCTCCTTCACCCCCTCC

V P L T T H N F A R K T F L K L N S S L E G G G G S G G G G S G G G>

_________________________________TRANSLATION OF CRAF RBD-GFP2 [A]___________________________________>

510 520 530 540 550 560 570 580 590 600

GGGCTCTGCGGCCGCAGGGAGTGGTATGGTGAGCAAGGGCGAGGAGCTGTTCACCGGGGTGGTGCCCATCCTGGTCGAGCTGGACGGCGACGTAAACGGC

CCCGAGACGCCGGCGTCCCTCACCATACCACTCGTTCCCGCTCCTCGACAAGTGGCCCCACCACGGGTAGGACCAGCTCGACCTGCCGCTGCATTTGCCG

G S A A A G S G M V S K G E E L F T G V V P I L V E L D G D V N G>

_________________________________TRANSLATION OF CRAF RBD-GFP2 [A]___________________________________>

610 620 630 640 650 660 670 680 690 700

CACAAGTTCAGCGTGTCCGGCGAGGGCGAGGGCGATGCCACCTACGGCAAGCTGACCCTGAAGTTCATCTGCACCACCGGCAAGCTGCCCGTGCCCTGGC

GTGTTCAAGTCGCACAGGCCGCTCCCGCTCCCGCTACGGTGGATGCCGTTCGACTGGGACTTCAAGTAGACGTGGTGGCCGTTCGACGGGCACGGGACCG

H K F S V S G E G E G D A T Y G K L T L K F I C T T G K L P V P W>

_________________________________TRANSLATION OF CRAF RBD-GFP2 [A]___________________________________>

710 720 730 740 750 760 770 780 790 800

CCACCCTCGTGACCACCCTGAGCTACGGCGTGCAGTGCTTCAGCCGCTACCCCGACCACATGAAGCAGCACGACTTCTTCAAGTCCGCCATGCCCGAAGG

GGTGGGAGCACTGGTGGGACTCGATGCCGCACGTCACGAAGTCGGCGATGGGGCTGGTGTACTTCGTCGTGCTGAAGAAGTTCAGGCGGTACGGGCTTCC

P T L V T T L S Y G V Q C F S R Y P D H M K Q H D F F K S A M P E G>

_________________________________TRANSLATION OF CRAF RBD-GFP2 [A]___________________________________>

810 820 830 840 850 860 870 880 890 900

CTACGTCCAGGAGCGCACCATCTTCTTCAAGGACGACGGCAACTACAAGACCCGCGCCGAGGTGAAGTTCGAGGGCGACACCCTGGTGAACCGCATCGAG

GATGCAGGTCCTCGCGTGGTAGAAGAAGTTCCTGCTGCCGTTGATGTTCTGGGCGCGGCTCCACTTCAAGCTCCCGCTGTGGGACCACTTGGCGTAGCTC

Y V Q E R T I F F K D D G N Y K T R A E V K F E G D T L V N R I E>

_________________________________TRANSLATION OF CRAF RBD-GFP2 [A]___________________________________>

910 920 930 940 950 960 970 980 990 1000

CTGAAGGGCATCGACTTCAAGGAGGACGGCAACATCCTGGGGCACAAGCTGGAGTACAACTACAACAGCCACAACGTCTATATCATGGCCGACAAGCAGA

GACTTCCCGTAGCTGAAGTTCCTCCTGCCGTTGTAGGACCCCGTGTTCGACCTCATGTTGATGTTGTCGGTGTTGCAGATATAGTACCGGCTGTTCGTCT

L K G I D F K E D G N I L G H K L E Y N Y N S H N V Y I M A D K Q>

_________________________________TRANSLATION OF CRAF RBD-GFP2 [A]___________________________________>

1010 1020 1030 1040 1050 1060 1070 1080 1090 1100

AGAACGGCATCAAGGTGAACTTCAAGATCCGCCACAACATCGAGGACGGCAGCGTGCAGCTCGCCGACCACTACCAGCAGAACACCCCCATCGGCGACGG

TCTTGCCGTAGTTCCACTTGAAGTTCTAGGCGGTGTTGTAGCTCCTGCCGTCGCACGTCGAGCGGCTGGTGATGGTCGTCTTGTGGGGGTAGCCGCTGCC

K N G I K V N F K I R H N I E D G S V Q L A D H Y Q Q N T P I G D G>

_________________________________TRANSLATION OF CRAF RBD-GFP2 [A]___________________________________>

1110 1120 1130 1140 1150 1160 1170 1180 1190 1200

CCCCGTGCTGCTGCCCGACAACCACTACCTGAGCACCCAGTCCGCCCTGAGCAAAGACCCCAACGAGAAGCGCGATCACATGGTCCTGCTGGAGTTCGTG

GGGGCACGACGACGGGCTGTTGGTGATGGACTCGTGGGTCAGGCGGGACTCGTTTCTGGGGTTGCTCTTCGCGCTAGTGTACCAGGACGACCTCAAGCAC

P V L L P D N H Y L S T Q S A L S K D P N E K R D H M V L L E F V>

_________________________________TRANSLATION OF CRAF RBD-GFP2 [A]___________________________________>

1210 1220 1230 1240

ACCGCCGCCGGGATCACTCTCGGCATGGACGAGCTGTACAAGTAA

TGGCGGCGGCCCTAGTGAGAGCCGTACCTGCTCGACATGTTCATT

T A A G I T L G M D E L Y K *>

______TRANSLATION OF CRAF RBD-GFP2 [A]_______>

Sequence: GFP2- CRAF full-length S257L Range: 1 to 2724

10 20 30 40 50 60 70 80 90 100

ATGGTGAGCAAGGGCGAGGAGCTGTTCACCGGGGTGGTGCCCATCCTGGTCGAGCTGGACGGCGACGTAAACGGCCACAAGTTCAGCGTGTCCGGCGAGG

TACCACTCGTTCCCGCTCCTCGACAAGTGGCCCCACCACGGGTAGGACCAGCTCGACCTGCCGCTGCATTTGCCGGTGTTCAAGTCGCACAGGCCGCTCC

M V S K G E E L F T G V V P I L V E L D G D V N G H K F S V S G E>

__________________________TRANSLATION OF GFP2- CRAF FULL-LENGTH S257L [A]___________________________>

110 120 130 140 150 160 170 180 190 200

GCGAGGGCGATGCCACCTACGGCAAGCTGACCCTGAAGTTCATCTGCACCACCGGCAAGCTGCCCGTGCCCTGGCCCACCCTCGTGACCACCCTGAGCTA

CGCTCCCGCTACGGTGGATGCCGTTCGACTGGGACTTCAAGTAGACGTGGTGGCCGTTCGACGGGCACGGGACCGGGTGGGAGCACTGGTGGGACTCGAT

G E G D A T Y G K L T L K F I C T T G K L P V P W P T L V T T L S Y>

__________________________TRANSLATION OF GFP2- CRAF FULL-LENGTH S257L [A]___________________________>

210 220 230 240 250 260 270 280 290 300

CGGCGTGCAGTGCTTCAGCCGCTACCCCGACCACATGAAGCAGCACGACTTCTTCAAGTCCGCCATGCCCGAAGGCTACGTCCAGGAGCGCACCATCTTC

GCCGCACGTCACGAAGTCGGCGATGGGGCTGGTGTACTTCGTCGTGCTGAAGAAGTTCAGGCGGTACGGGCTTCCGATGCAGGTCCTCGCGTGGTAGAAG

G V Q C F S R Y P D H M K Q H D F F K S A M P E G Y V Q E R T I F>

__________________________TRANSLATION OF GFP2- CRAF FULL-LENGTH S257L [A]___________________________>

310 320 330 340 350 360 370 380 390 400

TTCAAGGACGACGGCAACTACAAGACCCGCGCCGAGGTGAAGTTCGAGGGCGACACCCTGGTGAACCGCATCGAGCTGAAGGGCATCGACTTCAAGGAGG

AAGTTCCTGCTGCCGTTGATGTTCTGGGCGCGGCTCCACTTCAAGCTCCCGCTGTGGGACCACTTGGCGTAGCTCGACTTCCCGTAGCTGAAGTTCCTCC

F K D D G N Y K T R A E V K F E G D T L V N R I E L K G I D F K E>

__________________________TRANSLATION OF GFP2- CRAF FULL-LENGTH S257L [A]___________________________>

410 420 430 440 450 460 470 480 490 500

ACGGCAACATCCTGGGGCACAAGCTGGAGTACAACTACAACAGCCACAACGTCTATATCATGGCCGACAAGCAGAAGAACGGCATCAAGGTGAACTTCAA

TGCCGTTGTAGGACCCCGTGTTCGACCTCATGTTGATGTTGTCGGTGTTGCAGATATAGTACCGGCTGTTCGTCTTCTTGCCGTAGTTCCACTTGAAGTT

D G N I L G H K L E Y N Y N S H N V Y I M A D K Q K N G I K V N F K>

__________________________TRANSLATION OF GFP2- CRAF FULL-LENGTH S257L [A]___________________________>

510 520 530 540 550 560 570 580 590 600

GATCCGCCACAACATCGAGGACGGCAGCGTGCAGCTCGCCGACCACTACCAGCAGAACACCCCCATCGGCGACGGCCCCGTGCTGCTGCCCGACAACCAC

CTAGGCGGTGTTGTAGCTCCTGCCGTCGCACGTCGAGCGGCTGGTGATGGTCGTCTTGTGGGGGTAGCCGCTGCCGGGGCACGACGACGGGCTGTTGGTG

I R H N I E D G S V Q L A D H Y Q Q N T P I G D G P V L L P D N H>

__________________________TRANSLATION OF GFP2- CRAF FULL-LENGTH S257L [A]___________________________>

610 620 630 640 650 660 670 680 690 700

TACCTGAGCACCCAGTCCGCCCTGAGCAAAGACCCCAACGAGAAGCGCGATCACATGGTCCTGCTGGAGTTCGTGACCGCCGCCGGGATCACTCTCAGCA

ATGGACTCGTGGGTCAGGCGGGACTCGTTTCTGGGGTTGCTCTTCGCGCTAGTGTACCAGGACGACCTCAAGCACTGGCGGCGGCCCTAGTGAGAGTCGT

Y L S T Q S A L S K D P N E K R D H M V L L E F V T A A G I T L S>

__________________________TRANSLATION OF GFP2- CRAF FULL-LENGTH S257L [A]___________________________>

710 720 730 740 750 760 770 780 790 800

TGGACGAGCTGTACAAGCTCGAGGGCGGCGGAGGATCTGGGGGCGGAGGAAGTGGGGGAGGGGGCTCTGCGGCCGCCATGGAGCACATACAGGGAGCTTG

ACCTGCTCGACATGTTCGAGCTCCCGCCGCCTCCTAGACCCCCGCCTCCTTCACCCCCTCCCCCGAGACGCCGGCGGTACCTCGTGTATGTCCCTCGAAC

M D E L Y K L E G G G G S G G G G S G G G G S A A A M E H I Q G A W>

__________________________TRANSLATION OF GFP2- CRAF FULL-LENGTH S257L [A]___________________________>

810 820 830 840 850 860 870 880 890 900

GAAGACGATCAGCAATGGTTTTGGATTCAAAGATGCCGTGTTTGATGGCTCCAGCTGCATCTCTCCTACAATAGTTCAGCAGTTTGGCTATCAGCGCCGG

CTTCTGCTAGTCGTTACCAAAACCTAAGTTTCTACGGCACAAACTACCGAGGTCGACGTAGAGAGGATGTTATCAAGTCGTCAAACCGATAGTCGCGGCC

K T I S N G F G F K D A V F D G S S C I S P T I V Q Q F G Y Q R R>

__________________________TRANSLATION OF GFP2- CRAF FULL-LENGTH S257L [A]___________________________>

910 920 930 940 950 960 970 980 990 1000

GCATCAGATGATGGCAAACTCACAGATCCTTCTAAGACAAGCAACACTATCCGTGTTTTCTTGCCGAACAAGCAAAGAACAGTGGTCAATGTGCGAAATG

CGTAGTCTACTACCGTTTGAGTGTCTAGGAAGATTCTGTTCGTTGTGATAGGCACAAAAGAACGGCTTGTTCGTTTCTTGTCACCAGTTACACGCTTTAC

A S D D G K L T D P S K T S N T I R V F L P N K Q R T V V N V R N>

__________________________TRANSLATION OF GFP2- CRAF FULL-LENGTH S257L [A]___________________________>

1010 1020 1030 1040 1050 1060 1070 1080 1090 1100

GAATGAGCTTGCATGACTGCCTTATGAAAGCACTCAAGGTGAGGGGCCTGCAACCAGAGTGCTGTGCAGTGTTCAGACTTCTCCACGAACACAAAGGTAA

CTTACTCGAACGTACTGACGGAATACTTTCGTGAGTTCCACTCCCCGGACGTTGGTCTCACGACACGTCACAAGTCTGAAGAGGTGCTTGTGTTTCCATT

G M S L H D C L M K A L K V R G L Q P E C C A V F R L L H E H K G K>

__________________________TRANSLATION OF GFP2- CRAF FULL-LENGTH S257L [A]___________________________>

1110 1120 1130 1140 1150 1160 1170 1180 1190 1200

AAAAGCACGCTTAGATTGGAATACTGATGCTGCGTCTTTGATTGGAGAAGAACTTCAAGTAGATTTCCTGGATCATGTTCCCCTCACAACACACAACTTT

TTTTCGTGCGAATCTAACCTTATGACTACGACGCAGAAACTAACCTCTTCTTGAAGTTCATCTAAAGGACCTAGTACAAGGGGAGTGTTGTGTGTTGAAA

K A R L D W N T D A A S L I G E E L Q V D F L D H V P L T T H N F>

__________________________TRANSLATION OF GFP2- CRAF FULL-LENGTH S257L [A]___________________________>

1210 1220 1230 1240 1250 1260 1270 1280 1290 1300

GCTCGGAAGACGTTCCTGAAGCTTGCCTTCTGTGACATCTGTCAGAAATTCCTGCTCAATGGATTTCGATGTCAGACTTGTGGCTACAAATTTCATGAGC

CGAGCCTTCTGCAAGGACTTCGAACGGAAGACACTGTAGACAGTCTTTAAGGACGAGTTACCTAAAGCTACAGTCTGAACACCGATGTTTAAAGTACTCG

A R K T F L K L A F C D I C Q K F L L N G F R C Q T C G Y K F H E>

__________________________TRANSLATION OF GFP2- CRAF FULL-LENGTH S257L [A]___________________________>

1310 1320 1330 1340 1350 1360 1370 1380 1390 1400

ACTGTAGCACCAAAGTACCTACTATGTGTGTGGACTGGAGTAACATCAGACAACTCTTATTGTTTCCAAATTCCACTATTGGTGATAGTGGAGTCCCAGC

TGACATCGTGGTTTCATGGATGATACACACACCTGACCTCATTGTAGTCTGTTGAGAATAACAAAGGTTTAAGGTGATAACCACTATCACCTCAGGGTCG

H C S T K V P T M C V D W S N I R Q L L L F P N S T I G D S G V P A>

__________________________TRANSLATION OF GFP2- CRAF FULL-LENGTH S257L [A]___________________________>

1410 1420 1430 1440 1450 1460 1470 1480 1490 1500

ACTACCTTCTTTGACTATGCGTCGTATGCGAGAGTCTGTTTCCAGGATGCCTGTTAGTTCTCAGCACAGATATTCTACACCTCACGCCTTCACCTTTAAC

TGATGGAAGAAACTGATACGCAGCATACGCTCTCAGACAAAGGTCCTACGGACAATCAAGAGTCGTGTCTATAAGATGTGGAGTGCGGAAGTGGAAATTG

L P S L T M R R M R E S V S R M P V S S Q H R Y S T P H A F T F N>

__________________________TRANSLATION OF GFP2- CRAF FULL-LENGTH S257L [A]___________________________>

1510 1520 1530 1540 1550 1560 1570 1580 1590 1600

ACCTCCAGTCCCTCATCTGAAGGTTCCCTCTCCCAGAGGCAGAGGTTGACATCCACACCTAATGTCCACATGGTCAGCACCACCCTGCCTGTGGACAGCA

TGGAGGTCAGGGAGTAGACTTCCAAGGGAGAGGGTCTCCGTCTCCAACTGTAGGTGTGGATTACAGGTGTACCAGTCGTGGTGGGACGGACACCTGTCGT

T S S P S S E G S L S Q R Q R L T S T P N V H M V S T T L P V D S>

__________________________TRANSLATION OF GFP2- CRAF FULL-LENGTH S257L [A]___________________________>

1610 1620 1630 1640 1650 1660 1670 1680 1690 1700

GGATGATTGAGGATGCAATTCGAAGTCACAGCGAATCAGCCTCACCTTCAGCCCTGTCCAGTAGCCCCAACAATCTGAGCCCAACAGGCTGGTCACAGCC

CCTACTAACTCCTACGTTAAGCTTCAGTGTCGCTTAGTCGGAGTGGAAGTCGGGACAGGTCATCGGGGTTGTTAGACTCGGGTTGTCCGACCAGTGTCGG

R M I E D A I R S H S E S A S P S A L S S S P N N L S P T G W S Q P>

__________________________TRANSLATION OF GFP2- CRAF FULL-LENGTH S257L [A]___________________________>

1710 1720 1730 1740 1750 1760 1770 1780 1790 1800

GAAAACCCCCGTGCCAGCACAAAGAGAGCGGGCACCAGTATCTGGGACCCAGGAGAAAAACAAAATTAGGCCTCGTGGACAGAGAGATTCAAGCTATTAT

CTTTTGGGGGCACGGTCGTGTTTCTCTCGCCCGTGGTCATAGACCCTGGGTCCTCTTTTTGTTTTAATCCGGAGCACCTGTCTCTCTAAGTTCGATAATA

K T P V P A Q R E R A P V S G T Q E K N K I R P R G Q R D S S Y Y>

__________________________TRANSLATION OF GFP2- CRAF FULL-LENGTH S257L [A]___________________________>

1810 1820 1830 1840 1850 1860 1870 1880 1890 1900

TGGGAAATAGAAGCCAGTGAAGTGATGCTGTCCACTCGGATTGGGTCAGGCTCTTTTGGAACTGTTTATAAGGGTAAATGGCACGGAGATGTTGCAGTAA

ACCCTTTATCTTCGGTCACTTCACTACGACAGGTGAGCCTAACCCAGTCCGAGAAAACCTTGACAAATATTCCCATTTACCGTGCCTCTACAACGTCATT

W E I E A S E V M L S T R I G S G S F G T V Y K G K W H G D V A V>

__________________________TRANSLATION OF GFP2- CRAF FULL-LENGTH S257L [A]___________________________>

1910 1920 1930 1940 1950 1960 1970 1980 1990 2000

AGATCCTAAAGGTTGTCGACCCAACCCCAGAGCAATTCCAGGCCTTCAGGAATGAGGTGGCTGTTCTGCGCAAAACACGGCATGTGAACATTCTGCTTTT

TCTAGGATTTCCAACAGCTGGGTTGGGGTCTCGTTAAGGTCCGGAAGTCCTTACTCCACCGACAAGACGCGTTTTGTGCCGTACACTTGTAAGACGAAAA

K I L K V V D P T P E Q F Q A F R N E V A V L R K T R H V N I L L F>

__________________________TRANSLATION OF GFP2- CRAF FULL-LENGTH S257L [A]___________________________>

2010 2020 2030 2040 2050 2060 2070 2080 2090 2100

CATGGGGTACATGACAAAGGACAACCTGGCAATTGTGACCCAGTGGTGCGAGGGCAGCAGCCTCTACAAACACCTGCATGTCCAGGAGACCAAGTTTCAG

GTACCCCATGTACTGTTTCCTGTTGGACCGTTAACACTGGGTCACCACGCTCCCGTCGTCGGAGATGTTTGTGGACGTACAGGTCCTCTGGTTCAAAGTC

M G Y M T K D N L A I V T Q W C E G S S L Y K H L H V Q E T K F Q>

__________________________TRANSLATION OF GFP2- CRAF FULL-LENGTH S257L [A]___________________________>

2110 2120 2130 2140 2150 2160 2170 2180 2190 2200

ATGTTCCAGCTAATTGACATTGCCCGGCAGACGGCTCAGGGAATGGACTATTTGCATGCAAAGAACATCATCCATAGAGACATGAAATCCAACAATATAT

TACAAGGTCGATTAACTGTAACGGGCCGTCTGCCGAGTCCCTTACCTGATAAACGTACGTTTCTTGTAGTAGGTATCTCTGTACTTTAGGTTGTTATATA

M F Q L I D I A R Q T A Q G M D Y L H A K N I I H R D M K S N N I>

__________________________TRANSLATION OF GFP2- CRAF FULL-LENGTH S257L [A]___________________________>

2210 2220 2230 2240 2250 2260 2270 2280 2290 2300

TTCTCCATGAAGGCTTAACAGTGAAAATTGGAGATTTTGGTTTGGCAACAGTAAAGTCACGCTGGAGTGGTTCTCAGCAGGTTGAACAACCTACTGGCTC

AAGAGGTACTTCCGAATTGTCACTTTTAACCTCTAAAACCAAACCGTTGTCATTTCAGTGCGACCTCACCAAGAGTCGTCCAACTTGTTGGATGACCGAG

F L H E G L T V K I G D F G L A T V K S R W S G S Q Q V E Q P T G S>

__________________________TRANSLATION OF GFP2- CRAF FULL-LENGTH S257L [A]___________________________>

2310 2320 2330 2340 2350 2360 2370 2380 2390 2400

TGTCCTCTGGATGGCCCCAGAGGTGATCCGAATGCAGGATAACAACCCATTCAGTTTCCAGTCGGATGTCTACTCCTATGGCATCGTATTGTATGAACTG

ACAGGAGACCTACCGGGGTCTCCACTAGGCTTACGTCCTATTGTTGGGTAAGTCAAAGGTCAGCCTACAGATGAGGATACCGTAGCATAACATACTTGAC

V L W M A P E V I R M Q D N N P F S F Q S D V Y S Y G I V L Y E L>

__________________________TRANSLATION OF GFP2- CRAF FULL-LENGTH S257L [A]___________________________>

2410 2420 2430 2440 2450 2460 2470 2480 2490 2500

ATGACGGGGGAGCTTCCTTATTCTCACATCAACAACCGAGATCAGATCATCTTCATGGTGGGCCGAGGATATGCCTCCCCAGATCTTAGTAAGCTATATA

TACTGCCCCCTCGAAGGAATAAGAGTGTAGTTGTTGGCTCTAGTCTAGTAGAAGTACCACCCGGCTCCTATACGGAGGGGTCTAGAATCATTCGATATAT

M T G E L P Y S H I N N R D Q I I F M V G R G Y A S P D L S K L Y>

__________________________TRANSLATION OF GFP2- CRAF FULL-LENGTH S257L [A]___________________________>

2510 2520 2530 2540 2550 2560 2570 2580 2590 2600

AGAACTGCCCCAAAGCAATGAAGAGGCTGGTAGCTGACTGTGTGAAGAAAGTAAAGGAAGAGAGGCCTCTTTTTCCCCAGATCCTGTCTTCCATTGAGCT

TCTTGACGGGGTTTCGTTACTTCTCCGACCATCGACTGACACACTTCTTTCATTTCCTTCTCTCCGGAGAAAAAGGGGTCTAGGACAGAAGGTAACTCGA

K N C P K A M K R L V A D C V K K V K E E R P L F P Q I L S S I E L>

__________________________TRANSLATION OF GFP2- CRAF FULL-LENGTH S257L [A]___________________________>

2610 2620 2630 2640 2650 2660 2670 2680 2690 2700

GCTCCAACACTCTCTACCGAAGATCAACCGGAGCGCTTCCGAGCCATCCTTGCATCGGGCAGCCCACACTGAGGATATCAATGCTTGCACGCTGACCACG

CGAGGTTGTGAGAGATGGCTTCTAGTTGGCCTCGCGAAGGCTCGGTAGGAACGTAGCCCGTCGGGTGTGACTCCTATAGTTACGAACGTGCGACTGGTGC

L Q H S L P K I N R S A S E P S L H R A A H T E D I N A C T L T T>

__________________________TRANSLATION OF GFP2- CRAF FULL-LENGTH S257L [A]___________________________>

2710 2720

TCCCCGAGGCTGCCTGTCTTCTAG

AGGGGCTCCGACGGACAGAAGATC

S P R L P V F *>

___TRANSLATION OF GF____>

Sequence: RALGDS RA-GFP2 Range: 1 to 1083

10 20 30 40 50 60 70 80 90 100

ATGGCGCTGCCGCTCTACAACCAGCAGGTGGGCGACTGCTGCATCATCAGGGTCAGCCTGGATGTGGACAACGGCAACATGTACAAGAGCATCCTGGTGA

TACCGCGACGGCGAGATGTTGGTCGTCCACCCGCTGACGACGTAGTAGTCCCAGTCGGACCTACACCTGTTGCCGTTGTACATGTTCTCGTAGGACCACT

M A L P L Y N Q Q V G D C C I I R V S L D V D N G N M Y K S I L V>

_________________________________TRANSLATION OF RALGDS RA-GFP2 [A]__________________________________>

110 120 130 140 150 160 170 180 190 200

CCAGCCAGGATAAGGCTCCGACTGTCATCCGCAAGGCTATGGACAAACACAACCTAGATGAGGACGAGCCGGAGGATTATGAGCTGGTGCAGATCATCTC

GGTCGGTCCTATTCCGAGGCTGACAGTAGGCGTTCCGATACCTGTTTGTGTTGGATCTACTCCTGCTCGGCCTCCTAATACTCGACCACGTCTAGTAGAG

T S Q D K A P T V I R K A M D K H N L D E D E P E D Y E L V Q I I S>

_________________________________TRANSLATION OF RALGDS RA-GFP2 [A]__________________________________>

210 220 230 240 250 260 270 280 290 300

AGAGGATCACAAGCTGAAGATTCCAGAAAACGCCAATGTGTTCTATGCCATGAACTCTACCGCCAACTATGACTTTGTCCTCAAGAAGCGGACCCTCGAG

TCTCCTAGTGTTCGACTTCTAAGGTCTTTTGCGGTTACACAAGATACGGTACTTGAGATGGCGGTTGATACTGAAACAGGAGTTCTTCGCCTGGGAGCTC

E D H K L K I P E N A N V F Y A M N S T A N Y D F V L K K R T L E>

_________________________________TRANSLATION OF RALGDS RA-GFP2 [A]__________________________________>

310 320 330 340 350 360 370 380 390 400

GGCGGCGGAGGATCTGGGGGCGGAGGAAGTGGGGGAGGGGGCTCTGCGGCCGCAGGGAGTGGTATGGTGAGCAAGGGCGAGGAGCTGTTCACCGGGGTGG

CCGCCGCCTCCTAGACCCCCGCCTCCTTCACCCCCTCCCCCGAGACGCCGGCGTCCCTCACCATACCACTCGTTCCCGCTCCTCGACAAGTGGCCCCACC

G G G G S G G G G S G G G G S A A A G S G M V S K G E E L F T G V>

_________________________________TRANSLATION OF RALGDS RA-GFP2 [A]__________________________________>

410 420 430 440 450 460 470 480 490 500

TGCCCATCCTGGTCGAGCTGGACGGCGACGTAAACGGCCACAAGTTCAGCGTGTCCGGCGAGGGCGAGGGCGATGCCACCTACGGCAAGCTGACCCTGAA

ACGGGTAGGACCAGCTCGACCTGCCGCTGCATTTGCCGGTGTTCAAGTCGCACAGGCCGCTCCCGCTCCCGCTACGGTGGATGCCGTTCGACTGGGACTT

V P I L V E L D G D V N G H K F S V S G E G E G D A T Y G K L T L K>

_________________________________TRANSLATION OF RALGDS RA-GFP2 [A]__________________________________>

510 520 530 540 550 560 570 580 590 600

GTTCATCTGCACCACCGGCAAGCTGCCCGTGCCCTGGCCCACCCTCGTGACCACCCTGAGCTACGGCGTGCAGTGCTTCAGCCGCTACCCCGACCACATG

CAAGTAGACGTGGTGGCCGTTCGACGGGCACGGGACCGGGTGGGAGCACTGGTGGGACTCGATGCCGCACGTCACGAAGTCGGCGATGGGGCTGGTGTAC

F I C T T G K L P V P W P T L V T T L S Y G V Q C F S R Y P D H M>

_________________________________TRANSLATION OF RALGDS RA-GFP2 [A]__________________________________>

610 620 630 640 650 660 670 680 690 700

AAGCAGCACGACTTCTTCAAGTCCGCCATGCCCGAAGGCTACGTCCAGGAGCGCACCATCTTCTTCAAGGACGACGGCAACTACAAGACCCGCGCCGAGG

TTCGTCGTGCTGAAGAAGTTCAGGCGGTACGGGCTTCCGATGCAGGTCCTCGCGTGGTAGAAGAAGTTCCTGCTGCCGTTGATGTTCTGGGCGCGGCTCC

K Q H D F F K S A M P E G Y V Q E R T I F F K D D G N Y K T R A E>

_________________________________TRANSLATION OF RALGDS RA-GFP2 [A]__________________________________>

710 720 730 740 750 760 770 780 790 800

TGAAGTTCGAGGGCGACACCCTGGTGAACCGCATCGAGCTGAAGGGCATCGACTTCAAGGAGGACGGCAACATCCTGGGGCACAAGCTGGAGTACAACTA

ACTTCAAGCTCCCGCTGTGGGACCACTTGGCGTAGCTCGACTTCCCGTAGCTGAAGTTCCTCCTGCCGTTGTAGGACCCCGTGTTCGACCTCATGTTGAT

V K F E G D T L V N R I E L K G I D F K E D G N I L G H K L E Y N Y>

_________________________________TRANSLATION OF RALGDS RA-GFP2 [A]__________________________________>

810 820 830 840 850 860 870 880 890 900

CAACAGCCACAACGTCTATATCATGGCCGACAAGCAGAAGAACGGCATCAAGGTGAACTTCAAGATCCGCCACAACATCGAGGACGGCAGCGTGCAGCTC

GTTGTCGGTGTTGCAGATATAGTACCGGCTGTTCGTCTTCTTGCCGTAGTTCCACTTGAAGTTCTAGGCGGTGTTGTAGCTCCTGCCGTCGCACGTCGAG

N S H N V Y I M A D K Q K N G I K V N F K I R H N I E D G S V Q L>

_________________________________TRANSLATION OF RALGDS RA-GFP2 [A]__________________________________>

910 920 930 940 950 960 970 980 990 1000

GCCGACCACTACCAGCAGAACACCCCCATCGGCGACGGCCCCGTGCTGCTGCCCGACAACCACTACCTGAGCACCCAGTCCGCCCTGAGCAAAGACCCCA

CGGCTGGTGATGGTCGTCTTGTGGGGGTAGCCGCTGCCGGGGCACGACGACGGGCTGTTGGTGATGGACTCGTGGGTCAGGCGGGACTCGTTTCTGGGGT

A D H Y Q Q N T P I G D G P V L L P D N H Y L S T Q S A L S K D P>

_________________________________TRANSLATION OF RALGDS RA-GFP2 [A]__________________________________>

1010 1020 1030 1040 1050 1060 1070 1080

ACGAGAAGCGCGATCACATGGTCCTGCTGGAGTTCGTGACCGCCGCCGGGATCACTCTCGGCATGGACGAGCTGTACAAGTAA

TGCTCTTCGCGCTAGTGTACCAGGACGACCTCAAGCACTGGCGGCGGCCCTAGTGAGAGCCGTACCTGCTCGACATGTTCATT

N E K R D H M V L L E F V T A A G I T L G M D E L Y K *>

________________________TRANSLATION OF RALGDS RA-GFP2 [A]__________________________>

Sequence: RLuc8-KRASG12A full-length Range: 1 to 1560

10 20 30 40 50 60 70 80 90 100

ATGACCAGCAAGGTGTACGACCCCGAGCAGAGGAAGAGGATGATCACCGGCCCCCAGTGGTGGGCCAGGTGCAAGCAGATGAACGTGCTGGACAGCTTCA

TACTGGTCGTTCCACATGCTGGGGCTCGTCTCCTTCTCCTACTAGTGGCCGGGGGTCACCACCCGGTCCACGTTCGTCTACTTGCACGACCTGTCGAAGT

M T S K V Y D P E Q R K R M I T G P Q W W A R C K Q M N V L D S F>

___________________________TRANSLATION OF RLUC8-KRASG12A FULL-LENGTH [A]____________________________>

110 120 130 140 150 160 170 180 190 200

TCAACTACTACGACAGCGAGAAGCACGCCGAGAACGCCGTGATCTTCCTGCACGGCAACGCCACTAGCAGCTACCTGTGGAGGCACGTGGTGCCCCACAT

AGTTGATGATGCTGTCGCTCTTCGTGCGGCTCTTGCGGCACTAGAAGGACGTGCCGTTGCGGTGATCGTCGATGGACACCTCCGTGCACCACGGGGTGTA

I N Y Y D S E K H A E N A V I F L H G N A T S S Y L W R H V V P H I>

___________________________TRANSLATION OF RLUC8-KRASG12A FULL-LENGTH [A]____________________________>

210 220 230 240 250 260 270 280 290 300

CGAGCCCGTGGCCAGGTGCATCATCCCCGATCTGATCGGCATGGGCAAGAGCGGCAAGAGCGGCAACGGCAGCTACAGGCTGCTGGACCACTACAAGTAC

GCTCGGGCACCGGTCCACGTAGTAGGGGCTAGACTAGCCGTACCCGTTCTCGCCGTTCTCGCCGTTGCCGTCGATGTCCGACGACCTGGTGATGTTCATG

E P V A R C I I P D L I G M G K S G K S G N G S Y R L L D H Y K Y>

___________________________TRANSLATION OF RLUC8-KRASG12A FULL-LENGTH [A]____________________________>

310 320 330 340 350 360 370 380 390 400

CTGACCGCCTGGTTCGAGCTCCTGAACCTGCCCAAGAAGATCATCTTCGTGGGCCACGACTGGGGCGCCGCCCTGGCCTTCCACTACGCCTACGAGCACC

GACTGGCGGACCAAGCTCGAGGACTTGGACGGGTTCTTCTAGTAGAAGCACCCGGTGCTGACCCCGCGGCGGGACCGGAAGGTGATGCGGATGCTCGTGG

L T A W F E L L N L P K K I I F V G H D W G A A L A F H Y A Y E H>

___________________________TRANSLATION OF RLUC8-KRASG12A FULL-LENGTH [A]____________________________>

410 420 430 440 450 460 470 480 490 500

AGGACAGGATCAAGGCCATCGTGCACATGGAGAGCGTGGTGGACGTGATCGAGAGCTGGGACGAGTGGCCAGACATCGAGGAGGACATCGCCCTGATCAA

TCCTGTCCTAGTTCCGGTAGCACGTGTACCTCTCGCACCACCTGCACTAGCTCTCGACCCTGCTCACCGGTCTGTAGCTCCTCCTGTAGCGGGACTAGTT

Q D R I K A I V H M E S V V D V I E S W D E W P D I E E D I A L I K>

___________________________TRANSLATION OF RLUC8-KRASG12A FULL-LENGTH [A]____________________________>

510 520 530 540 550 560 570 580 590 600

GAGCGAGGAGGGCGAGAAGATGGTGCTGGAGAACAACTTCTTCGTGGAGACCGTGCTGCCCAGCAAGATCATGAGAAAGCTGGAGCCCGAGGAGTTCGCC

CTCGCTCCTCCCGCTCTTCTACCACGACCTCTTGTTGAAGAAGCACCTCTGGCACGACGGGTCGTTCTAGTACTCTTTCGACCTCGGGCTCCTCAAGCGG

S E E G E K M V L E N N F F V E T V L P S K I M R K L E P E E F A>

___________________________TRANSLATION OF RLUC8-KRASG12A FULL-LENGTH [A]____________________________>

610 620 630 640 650 660 670 680 690 700

GCCTACCTGGAGCCCTTCAAGGAGAAGGGCGAGGTGAGAAGACCCACCCTGAGCTGGCCCAGAGAGATCCCCCTGGTGAAGGGCGGCAAGCCCGACGTGG

CGGATGGACCTCGGGAAGTTCCTCTTCCCGCTCCACTCTTCTGGGTGGGACTCGACCGGGTCTCTCTAGGGGGACCACTTCCCGCCGTTCGGGCTGCACC

A Y L E P F K E K G E V R R P T L S W P R E I P L V K G G K P D V>

___________________________TRANSLATION OF RLUC8-KRASG12A FULL-LENGTH [A]____________________________>

710 720 730 740 750 760 770 780 790 800

TGCAGATCGTGAGAAACTACAACGCCTACCTGAGAGCCAGCGACGACCTGCCCAAGCTGTTCATCGAGAGCGACCCCGGCTTCTTCAGCAACGCCATCGT

ACGTCTAGCACTCTTTGATGTTGCGGATGGACTCTCGGTCGCTGCTGGACGGGTTCGACAAGTAGCTCTCGCTGGGGCCGAAGAAGTCGTTGCGGTAGCA

V Q I V R N Y N A Y L R A S D D L P K L F I E S D P G F F S N A I V>

___________________________TRANSLATION OF RLUC8-KRASG12A FULL-LENGTH [A]____________________________>

810 820 830 840 850 860 870 880 890 900

GGAGGGCGCCAAGAAGTTCCCCAACACCGAGTTCGTGAAGGTGAAGGGCCTGCACTTCCTCCAGGAGGACGCCCCCGACGAGATGGGCAAGTACATCAAG

CCTCCCGCGGTTCTTCAAGGGGTTGTGGCTCAAGCACTTCCACTTCCCGGACGTGAAGGAGGTCCTCCTGCGGGGGCTGCTCTACCCGTTCATGTAGTTC

E G A K K F P N T E F V K V K G L H F L Q E D A P D E M G K Y I K>

___________________________TRANSLATION OF RLUC8-KRASG12A FULL-LENGTH [A]____________________________>

910 920 930 940 950 960 970 980 990 1000

AGCTTCGTGGAGAGAGTGCTGAAGAACGAGCAGCTCGAGGGCGGCGGAGGATCTGGGGGCGGAGGAAGTGGGGGAGGGGGCTCTGCGGCCGCTATGACCG

TCGAAGCACCTCTCTCACGACTTCTTGCTCGTCGAGCTCCCGCCGCCTCCTAGACCCCCGCCTCCTTCACCCCCTCCCCCGAGACGCCGGCGATACTGGC

S F V E R V L K N E Q L E G G G G S G G G G S G G G G S A A A M T>

___________________________TRANSLATION OF RLUC8-KRASG12A FULL-LENGTH [A]____________________________>

1010 1020 1030 1040 1050 1060 1070 1080 1090 1100

AATATAAACTTGTGGTAGTTGGAGCTGCTGGCGTAGGCAAGAGTGCCTTGACGATACAGCTAATTCAGAATCATTTTGTGGACGAATATGATCCAACAAT

TTATATTTGAACACCATCAACCTCGACGACCGCATCCGTTCTCACGGAACTGCTATGTCGATTAAGTCTTAGTAAAACACCTGCTTATACTAGGTTGTTA

E Y K L V V V G A A G V G K S A L T I Q L I Q N H F V D E Y D P T I>

___________________________TRANSLATION OF RLUC8-KRASG12A FULL-LENGTH [A]____________________________>

1110 1120 1130 1140 1150 1160 1170 1180 1190 1200

AGAGGATTCCTACAGGAAGCAAGTAGTAATTGATGGAGAAACCTGTCTCTTGGATATTCTCGACACAGCAGGTCAAGAGGAGTACAGTGCAATGAGGGAC

TCTCCTAAGGATGTCCTTCGTTCATCATTAACTACCTCTTTGGACAGAGAACCTATAAGAGCTGTGTCGTCCAGTTCTCCTCATGTCACGTTACTCCCTG

E D S Y R K Q V V I D G E T C L L D I L D T A G Q E E Y S A M R D>

___________________________TRANSLATION OF RLUC8-KRASG12A FULL-LENGTH [A]____________________________>

1210 1220 1230 1240 1250 1260 1270 1280 1290 1300

CAGTACATGAGGACTGGGGAGGGCTTTCTTTGTGTATTTGCCATAAATAATACTAAATCATTTGAAGATATTCACCATTATAGAGAACAAATTAAAAGAG

GTCATGTACTCCTGACCCCTCCCGAAAGAAACACATAAACGGTATTTATTATGATTTAGTAAACTTCTATAAGTGGTAATATCTCTTGTTTAATTTTCTC

Q Y M R T G E G F L C V F A I N N T K S F E D I H H Y R E Q I K R>

___________________________TRANSLATION OF RLUC8-KRASG12A FULL-LENGTH [A]____________________________>

1310 1320 1330 1340 1350 1360 1370 1380 1390 1400

TTAAGGACTCTGAAGATGTACCTATGGTCCTAGTAGGAAATAAATGTGATTTGCCTTCCAGAACAGTAGACACAAAACAGGCTCAGGACTTAGCAAGAAG

AATTCCTGAGACTTCTACATGGATACCAGGATCATCCTTTATTTACACTAAACGGAAGGTCTTGTCATCTGTGTTTTGTCCGAGTCCTGAATCGTTCTTC

V K D S E D V P M V L V G N K C D L P S R T V D T K Q A Q D L A R S>

___________________________TRANSLATION OF RLUC8-KRASG12A FULL-LENGTH [A]____________________________>

1410 1420 1430 1440 1450 1460 1470 1480 1490 1500

TTATGGAATTCCTTTTATTGAAACATCAGCAAAGACAAGACAGGGTGTTGATGATGCCTTCTATACATTAGTTCGAGAAATTCGAAAACATAAAGAAAAG

AATACCTTAAGGAAAATAACTTTGTAGTCGTTTCTGTTCTGTCCCACAACTACTACGGAAGATATGTAATCAAGCTCTTTAAGCTTTTGTATTTCTTTTC

Y G I P F I E T S A K T R Q G V D D A F Y T L V R E I R K H K E K>

___________________________TRANSLATION OF RLUC8-KRASG12A FULL-LENGTH [A]____________________________>

1510 1520 1530 1540 1550 1560

ATGAGCAAAGATGGTAAAAAGAAGAAAAAGAAGTCAAAGACAAAGTGTGTAATTATGTAA

TACTCGTTTCTACCATTTTTCTTCTTTTTCTTCAGTTTCTGTTTCACACATTAATACATT

M S K D G K K K K K K S K T K C V I M *>

_______TRANSLATION OF RLUC8-KRASG12A FULL-LENGTH [A]________>

Sequence: RLuc8-KRASG12C full-length Range: 1 to 1560

10 20 30 40 50 60 70 80 90 100

ATGACCAGCAAGGTGTACGACCCCGAGCAGAGGAAGAGGATGATCACCGGCCCCCAGTGGTGGGCCAGGTGCAAGCAGATGAACGTGCTGGACAGCTTCA

TACTGGTCGTTCCACATGCTGGGGCTCGTCTCCTTCTCCTACTAGTGGCCGGGGGTCACCACCCGGTCCACGTTCGTCTACTTGCACGACCTGTCGAAGT

M T S K V Y D P E Q R K R M I T G P Q W W A R C K Q M N V L D S F>

___________________________TRANSLATION OF RLUC8-KRASG12C FULL-LENGTH [A]____________________________>

110 120 130 140 150 160 170 180 190 200

TCAACTACTACGACAGCGAGAAGCACGCCGAGAACGCCGTGATCTTCCTGCACGGCAACGCCACTAGCAGCTACCTGTGGAGGCACGTGGTGCCCCACAT

AGTTGATGATGCTGTCGCTCTTCGTGCGGCTCTTGCGGCACTAGAAGGACGTGCCGTTGCGGTGATCGTCGATGGACACCTCCGTGCACCACGGGGTGTA

I N Y Y D S E K H A E N A V I F L H G N A T S S Y L W R H V V P H I>

___________________________TRANSLATION OF RLUC8-KRASG12C FULL-LENGTH [A]____________________________>

210 220 230 240 250 260 270 280 290 300

CGAGCCCGTGGCCAGGTGCATCATCCCCGATCTGATCGGCATGGGCAAGAGCGGCAAGAGCGGCAACGGCAGCTACAGGCTGCTGGACCACTACAAGTAC

GCTCGGGCACCGGTCCACGTAGTAGGGGCTAGACTAGCCGTACCCGTTCTCGCCGTTCTCGCCGTTGCCGTCGATGTCCGACGACCTGGTGATGTTCATG

E P V A R C I I P D L I G M G K S G K S G N G S Y R L L D H Y K Y>

___________________________TRANSLATION OF RLUC8-KRASG12C FULL-LENGTH [A]____________________________>

310 320 330 340 350 360 370 380 390 400

CTGACCGCCTGGTTCGAGCTCCTGAACCTGCCCAAGAAGATCATCTTCGTGGGCCACGACTGGGGCGCCGCCCTGGCCTTCCACTACGCCTACGAGCACC

GACTGGCGGACCAAGCTCGAGGACTTGGACGGGTTCTTCTAGTAGAAGCACCCGGTGCTGACCCCGCGGCGGGACCGGAAGGTGATGCGGATGCTCGTGG

L T A W F E L L N L P K K I I F V G H D W G A A L A F H Y A Y E H>

___________________________TRANSLATION OF RLUC8-KRASG12C FULL-LENGTH [A]____________________________>

410 420 430 440 450 460 470 480 490 500

AGGACAGGATCAAGGCCATCGTGCACATGGAGAGCGTGGTGGACGTGATCGAGAGCTGGGACGAGTGGCCAGACATCGAGGAGGACATCGCCCTGATCAA

TCCTGTCCTAGTTCCGGTAGCACGTGTACCTCTCGCACCACCTGCACTAGCTCTCGACCCTGCTCACCGGTCTGTAGCTCCTCCTGTAGCGGGACTAGTT

Q D R I K A I V H M E S V V D V I E S W D E W P D I E E D I A L I K>

___________________________TRANSLATION OF RLUC8-KRASG12C FULL-LENGTH [A]____________________________>

510 520 530 540 550 560 570 580 590 600

GAGCGAGGAGGGCGAGAAGATGGTGCTGGAGAACAACTTCTTCGTGGAGACCGTGCTGCCCAGCAAGATCATGAGAAAGCTGGAGCCCGAGGAGTTCGCC

CTCGCTCCTCCCGCTCTTCTACCACGACCTCTTGTTGAAGAAGCACCTCTGGCACGACGGGTCGTTCTAGTACTCTTTCGACCTCGGGCTCCTCAAGCGG

S E E G E K M V L E N N F F V E T V L P S K I M R K L E P E E F A>

___________________________TRANSLATION OF RLUC8-KRASG12C FULL-LENGTH [A]____________________________>

610 620 630 640 650 660 670 680 690 700

GCCTACCTGGAGCCCTTCAAGGAGAAGGGCGAGGTGAGAAGACCCACCCTGAGCTGGCCCAGAGAGATCCCCCTGGTGAAGGGCGGCAAGCCCGACGTGG

CGGATGGACCTCGGGAAGTTCCTCTTCCCGCTCCACTCTTCTGGGTGGGACTCGACCGGGTCTCTCTAGGGGGACCACTTCCCGCCGTTCGGGCTGCACC

A Y L E P F K E K G E V R R P T L S W P R E I P L V K G G K P D V>

___________________________TRANSLATION OF RLUC8-KRASG12C FULL-LENGTH [A]____________________________>

710 720 730 740 750 760 770 780 790 800

TGCAGATCGTGAGAAACTACAACGCCTACCTGAGAGCCAGCGACGACCTGCCCAAGCTGTTCATCGAGAGCGACCCCGGCTTCTTCAGCAACGCCATCGT

ACGTCTAGCACTCTTTGATGTTGCGGATGGACTCTCGGTCGCTGCTGGACGGGTTCGACAAGTAGCTCTCGCTGGGGCCGAAGAAGTCGTTGCGGTAGCA

V Q I V R N Y N A Y L R A S D D L P K L F I E S D P G F F S N A I V>

___________________________TRANSLATION OF RLUC8-KRASG12C FULL-LENGTH [A]____________________________>

810 820 830 840 850 860 870 880 890 900

GGAGGGCGCCAAGAAGTTCCCCAACACCGAGTTCGTGAAGGTGAAGGGCCTGCACTTCCTCCAGGAGGACGCCCCCGACGAGATGGGCAAGTACATCAAG

CCTCCCGCGGTTCTTCAAGGGGTTGTGGCTCAAGCACTTCCACTTCCCGGACGTGAAGGAGGTCCTCCTGCGGGGGCTGCTCTACCCGTTCATGTAGTTC

E G A K K F P N T E F V K V K G L H F L Q E D A P D E M G K Y I K>

___________________________TRANSLATION OF RLUC8-KRASG12C FULL-LENGTH [A]____________________________>

910 920 930 940 950 960 970 980 990 1000

AGCTTCGTGGAGAGAGTGCTGAAGAACGAGCAGCTCGAGGGCGGCGGAGGATCTGGGGGCGGAGGAAGTGGGGGAGGGGGCTCTGCGGCCGCTATGACCG

TCGAAGCACCTCTCTCACGACTTCTTGCTCGTCGAGCTCCCGCCGCCTCCTAGACCCCCGCCTCCTTCACCCCCTCCCCCGAGACGCCGGCGATACTGGC

S F V E R V L K N E Q L E G G G G S G G G G S G G G G S A A A M T>

___________________________TRANSLATION OF RLUC8-KRASG12C FULL-LENGTH [A]____________________________>

1010 1020 1030 1040 1050 1060 1070 1080 1090 1100

AATATAAACTTGTGGTAGTTGGAGCTTGTGGCGTAGGCAAGAGTGCCTTGACGATACAGCTAATTCAGAATCATTTTGTGGACGAATATGATCCAACAAT

TTATATTTGAACACCATCAACCTCGAACACCGCATCCGTTCTCACGGAACTGCTATGTCGATTAAGTCTTAGTAAAACACCTGCTTATACTAGGTTGTTA

E Y K L V V V G A C G V G K S A L T I Q L I Q N H F V D E Y D P T I>

___________________________TRANSLATION OF RLUC8-KRASG12C FULL-LENGTH [A]____________________________>

1110 1120 1130 1140 1150 1160 1170 1180 1190 1200

AGAGGATTCCTACAGGAAGCAAGTAGTAATTGATGGAGAAACCTGTCTCTTGGATATTCTCGACACAGCAGGTCAAGAGGAGTACAGTGCAATGAGGGAC

TCTCCTAAGGATGTCCTTCGTTCATCATTAACTACCTCTTTGGACAGAGAACCTATAAGAGCTGTGTCGTCCAGTTCTCCTCATGTCACGTTACTCCCTG

E D S Y R K Q V V I D G E T C L L D I L D T A G Q E E Y S A M R D>

___________________________TRANSLATION OF RLUC8-KRASG12C FULL-LENGTH [A]____________________________>

1210 1220 1230 1240 1250 1260 1270 1280 1290 1300

CAGTACATGAGGACTGGGGAGGGCTTTCTTTGTGTATTTGCCATAAATAATACTAAATCATTTGAAGATATTCACCATTATAGAGAACAAATTAAAAGAG

GTCATGTACTCCTGACCCCTCCCGAAAGAAACACATAAACGGTATTTATTATGATTTAGTAAACTTCTATAAGTGGTAATATCTCTTGTTTAATTTTCTC

Q Y M R T G E G F L C V F A I N N T K S F E D I H H Y R E Q I K R>

___________________________TRANSLATION OF RLUC8-KRASG12C FULL-LENGTH [A]____________________________>

1310 1320 1330 1340 1350 1360 1370 1380 1390 1400

TTAAGGACTCTGAAGATGTACCTATGGTCCTAGTAGGAAATAAATGTGATTTGCCTTCCAGAACAGTAGACACAAAACAGGCTCAGGACTTAGCAAGAAG

AATTCCTGAGACTTCTACATGGATACCAGGATCATCCTTTATTTACACTAAACGGAAGGTCTTGTCATCTGTGTTTTGTCCGAGTCCTGAATCGTTCTTC

V K D S E D V P M V L V G N K C D L P S R T V D T K Q A Q D L A R S>

___________________________TRANSLATION OF RLUC8-KRASG12C FULL-LENGTH [A]____________________________>

1410 1420 1430 1440 1450 1460 1470 1480 1490 1500

TTATGGAATTCCTTTTATTGAAACATCAGCAAAGACAAGACAGGGTGTTGATGATGCCTTCTATACATTAGTTCGAGAAATTCGAAAACATAAAGAAAAG

AATACCTTAAGGAAAATAACTTTGTAGTCGTTTCTGTTCTGTCCCACAACTACTACGGAAGATATGTAATCAAGCTCTTTAAGCTTTTGTATTTCTTTTC

Y G I P F I E T S A K T R Q G V D D A F Y T L V R E I R K H K E K>

___________________________TRANSLATION OF RLUC8-KRASG12C FULL-LENGTH [A]____________________________>

1510 1520 1530 1540 1550 1560

ATGAGCAAAGATGGTAAAAAGAAGAAAAAGAAGTCAAAGACAAAGTGTGTAATTATGTAA

TACTCGTTTCTACCATTTTTCTTCTTTTTCTTCAGTTTCTGTTTCACACATTAATACATT

M S K D G K K K K K K S K T K C V I M *>

_______TRANSLATION OF RLUC8-KRASG12C FULL-LENGTH [A]________>

Sequence: RLuc8-KRASG12D full-length Range: 1 to 1560

10 20 30 40 50 60 70 80 90 100

ATGACCAGCAAGGTGTACGACCCCGAGCAGAGGAAGAGGATGATCACCGGCCCCCAGTGGTGGGCCAGGTGCAAGCAGATGAACGTGCTGGACAGCTTCA

TACTGGTCGTTCCACATGCTGGGGCTCGTCTCCTTCTCCTACTAGTGGCCGGGGGTCACCACCCGGTCCACGTTCGTCTACTTGCACGACCTGTCGAAGT

M T S K V Y D P E Q R K R M I T G P Q W W A R C K Q M N V L D S F>

___________________________TRANSLATION OF RLUC8-KRASG12D FULL-LENGTH [A]____________________________>

110 120 130 140 150 160 170 180 190 200

TCAACTACTACGACAGCGAGAAGCACGCCGAGAACGCCGTGATCTTCCTGCACGGCAACGCCACTAGCAGCTACCTGTGGAGGCACGTGGTGCCCCACAT

AGTTGATGATGCTGTCGCTCTTCGTGCGGCTCTTGCGGCACTAGAAGGACGTGCCGTTGCGGTGATCGTCGATGGACACCTCCGTGCACCACGGGGTGTA

I N Y Y D S E K H A E N A V I F L H G N A T S S Y L W R H V V P H I>

___________________________TRANSLATION OF RLUC8-KRASG12D FULL-LENGTH [A]____________________________>

210 220 230 240 250 260 270 280 290 300

CGAGCCCGTGGCCAGGTGCATCATCCCCGATCTGATCGGCATGGGCAAGAGCGGCAAGAGCGGCAACGGCAGCTACAGGCTGCTGGACCACTACAAGTAC

GCTCGGGCACCGGTCCACGTAGTAGGGGCTAGACTAGCCGTACCCGTTCTCGCCGTTCTCGCCGTTGCCGTCGATGTCCGACGACCTGGTGATGTTCATG

E P V A R C I I P D L I G M G K S G K S G N G S Y R L L D H Y K Y>

___________________________TRANSLATION OF RLUC8-KRASG12D FULL-LENGTH [A]____________________________>

310 320 330 340 350 360 370 380 390 400

CTGACCGCCTGGTTCGAGCTCCTGAACCTGCCCAAGAAGATCATCTTCGTGGGCCACGACTGGGGCGCCGCCCTGGCCTTCCACTACGCCTACGAGCACC

GACTGGCGGACCAAGCTCGAGGACTTGGACGGGTTCTTCTAGTAGAAGCACCCGGTGCTGACCCCGCGGCGGGACCGGAAGGTGATGCGGATGCTCGTGG

L T A W F E L L N L P K K I I F V G H D W G A A L A F H Y A Y E H>

___________________________TRANSLATION OF RLUC8-KRASG12D FULL-LENGTH [A]____________________________>

410 420 430 440 450 460 470 480 490 500

AGGACAGGATCAAGGCCATCGTGCACATGGAGAGCGTGGTGGACGTGATCGAGAGCTGGGACGAGTGGCCAGACATCGAGGAGGACATCGCCCTGATCAA

TCCTGTCCTAGTTCCGGTAGCACGTGTACCTCTCGCACCACCTGCACTAGCTCTCGACCCTGCTCACCGGTCTGTAGCTCCTCCTGTAGCGGGACTAGTT

Q D R I K A I V H M E S V V D V I E S W D E W P D I E E D I A L I K>

___________________________TRANSLATION OF RLUC8-KRASG12D FULL-LENGTH [A]____________________________>

510 520 530 540 550 560 570 580 590 600

GAGCGAGGAGGGCGAGAAGATGGTGCTGGAGAACAACTTCTTCGTGGAGACCGTGCTGCCCAGCAAGATCATGAGAAAGCTGGAGCCCGAGGAGTTCGCC

CTCGCTCCTCCCGCTCTTCTACCACGACCTCTTGTTGAAGAAGCACCTCTGGCACGACGGGTCGTTCTAGTACTCTTTCGACCTCGGGCTCCTCAAGCGG

S E E G E K M V L E N N F F V E T V L P S K I M R K L E P E E F A>

___________________________TRANSLATION OF RLUC8-KRASG12D FULL-LENGTH [A]____________________________>

610 620 630 640 650 660 670 680 690 700

GCCTACCTGGAGCCCTTCAAGGAGAAGGGCGAGGTGAGAAGACCCACCCTGAGCTGGCCCAGAGAGATCCCCCTGGTGAAGGGCGGCAAGCCCGACGTGG

CGGATGGACCTCGGGAAGTTCCTCTTCCCGCTCCACTCTTCTGGGTGGGACTCGACCGGGTCTCTCTAGGGGGACCACTTCCCGCCGTTCGGGCTGCACC

A Y L E P F K E K G E V R R P T L S W P R E I P L V K G G K P D V>

___________________________TRANSLATION OF RLUC8-KRASG12D FULL-LENGTH [A]____________________________>

710 720 730 740 750 760 770 780 790 800

TGCAGATCGTGAGAAACTACAACGCCTACCTGAGAGCCAGCGACGACCTGCCCAAGCTGTTCATCGAGAGCGACCCCGGCTTCTTCAGCAACGCCATCGT

ACGTCTAGCACTCTTTGATGTTGCGGATGGACTCTCGGTCGCTGCTGGACGGGTTCGACAAGTAGCTCTCGCTGGGGCCGAAGAAGTCGTTGCGGTAGCA

V Q I V R N Y N A Y L R A S D D L P K L F I E S D P G F F S N A I V>

___________________________TRANSLATION OF RLUC8-KRASG12D FULL-LENGTH [A]____________________________>

810 820 830 840 850 860 870 880 890 900

GGAGGGCGCCAAGAAGTTCCCCAACACCGAGTTCGTGAAGGTGAAGGGCCTGCACTTCCTCCAGGAGGACGCCCCCGACGAGATGGGCAAGTACATCAAG

CCTCCCGCGGTTCTTCAAGGGGTTGTGGCTCAAGCACTTCCACTTCCCGGACGTGAAGGAGGTCCTCCTGCGGGGGCTGCTCTACCCGTTCATGTAGTTC

E G A K K F P N T E F V K V K G L H F L Q E D A P D E M G K Y I K>

___________________________TRANSLATION OF RLUC8-KRASG12D FULL-LENGTH [A]____________________________>

910 920 930 940 950 960 970 980 990 1000

AGCTTCGTGGAGAGAGTGCTGAAGAACGAGCAGCTCGAGGGCGGCGGAGGATCTGGGGGCGGAGGAAGTGGGGGAGGGGGCTCTGCGGCCGCTATGACCG

TCGAAGCACCTCTCTCACGACTTCTTGCTCGTCGAGCTCCCGCCGCCTCCTAGACCCCCGCCTCCTTCACCCCCTCCCCCGAGACGCCGGCGATACTGGC

S F V E R V L K N E Q L E G G G G S G G G G S G G G G S A A A M T>

___________________________TRANSLATION OF RLUC8-KRASG12D FULL-LENGTH [A]____________________________>

1010 1020 1030 1040 1050 1060 1070 1080 1090 1100

AATATAAACTTGTGGTAGTTGGAGCTGACGGCGTAGGCAAGAGTGCCTTGACGATACAGCTAATTCAGAATCATTTTGTGGACGAATATGATCCAACAAT

TTATATTTGAACACCATCAACCTCGACTGCCGCATCCGTTCTCACGGAACTGCTATGTCGATTAAGTCTTAGTAAAACACCTGCTTATACTAGGTTGTTA

E Y K L V V V G A D G V G K S A L T I Q L I Q N H F V D E Y D P T I>

___________________________TRANSLATION OF RLUC8-KRASG12D FULL-LENGTH [A]____________________________>

1110 1120 1130 1140 1150 1160 1170 1180 1190 1200

AGAGGATTCCTACAGGAAGCAAGTAGTAATTGATGGAGAAACCTGTCTCTTGGATATTCTCGACACAGCAGGTCAAGAGGAGTACAGTGCAATGAGGGAC

TCTCCTAAGGATGTCCTTCGTTCATCATTAACTACCTCTTTGGACAGAGAACCTATAAGAGCTGTGTCGTCCAGTTCTCCTCATGTCACGTTACTCCCTG

E D S Y R K Q V V I D G E T C L L D I L D T A G Q E E Y S A M R D>

___________________________TRANSLATION OF RLUC8-KRASG12D FULL-LENGTH [A]____________________________>

1210 1220 1230 1240 1250 1260 1270 1280 1290 1300

CAGTACATGAGGACTGGGGAGGGCTTTCTTTGTGTATTTGCCATAAATAATACTAAATCATTTGAAGATATTCACCATTATAGAGAACAAATTAAAAGAG

GTCATGTACTCCTGACCCCTCCCGAAAGAAACACATAAACGGTATTTATTATGATTTAGTAAACTTCTATAAGTGGTAATATCTCTTGTTTAATTTTCTC

Q Y M R T G E G F L C V F A I N N T K S F E D I H H Y R E Q I K R>

___________________________TRANSLATION OF RLUC8-KRASG12D FULL-LENGTH [A]____________________________>

1310 1320 1330 1340 1350 1360 1370 1380 1390 1400

TTAAGGACTCTGAAGATGTACCTATGGTCCTAGTAGGAAATAAATGTGATTTGCCTTCCAGAACAGTAGACACAAAACAGGCTCAGGACTTAGCAAGAAG

AATTCCTGAGACTTCTACATGGATACCAGGATCATCCTTTATTTACACTAAACGGAAGGTCTTGTCATCTGTGTTTTGTCCGAGTCCTGAATCGTTCTTC

V K D S E D V P M V L V G N K C D L P S R T V D T K Q A Q D L A R S>

___________________________TRANSLATION OF RLUC8-KRASG12D FULL-LENGTH [A]____________________________>

1410 1420 1430 1440 1450 1460 1470 1480 1490 1500

TTATGGAATTCCTTTTATTGAAACATCAGCAAAGACAAGACAGGGTGTTGATGATGCCTTCTATACATTAGTTCGAGAAATTCGAAAACATAAAGAAAAG

AATACCTTAAGGAAAATAACTTTGTAGTCGTTTCTGTTCTGTCCCACAACTACTACGGAAGATATGTAATCAAGCTCTTTAAGCTTTTGTATTTCTTTTC

Y G I P F I E T S A K T R Q G V D D A F Y T L V R E I R K H K E K>

___________________________TRANSLATION OF RLUC8-KRASG12D FULL-LENGTH [A]____________________________>

1510 1520 1530 1540 1550 1560

ATGAGCAAAGATGGTAAAAAGAAGAAAAAGAAGTCAAAGACAAAGTGTGTAATTATGTAA

TACTCGTTTCTACCATTTTTCTTCTTTTTCTTCAGTTTCTGTTTCACACATTAATACATT

M S K D G K K K K K K S K T K C V I M *>

_______TRANSLATION OF RLUC8-KRASG12D FULL-LENGTH [A]________>

Sequence: RLuc8-KRASG12R full-length Range: 1 to 1560

10 20 30 40 50 60 70 80 90 100

ATGACCAGCAAGGTGTACGACCCCGAGCAGAGGAAGAGGATGATCACCGGCCCCCAGTGGTGGGCCAGGTGCAAGCAGATGAACGTGCTGGACAGCTTCA

TACTGGTCGTTCCACATGCTGGGGCTCGTCTCCTTCTCCTACTAGTGGCCGGGGGTCACCACCCGGTCCACGTTCGTCTACTTGCACGACCTGTCGAAGT

M T S K V Y D P E Q R K R M I T G P Q W W A R C K Q M N V L D S F>

___________________________TRANSLATION OF RLUC8-KRASG12R FULL-LENGTH [A]____________________________>

110 120 130 140 150 160 170 180 190 200

TCAACTACTACGACAGCGAGAAGCACGCCGAGAACGCCGTGATCTTCCTGCACGGCAACGCCACTAGCAGCTACCTGTGGAGGCACGTGGTGCCCCACAT

AGTTGATGATGCTGTCGCTCTTCGTGCGGCTCTTGCGGCACTAGAAGGACGTGCCGTTGCGGTGATCGTCGATGGACACCTCCGTGCACCACGGGGTGTA

I N Y Y D S E K H A E N A V I F L H G N A T S S Y L W R H V V P H I>

___________________________TRANSLATION OF RLUC8-KRASG12R FULL-LENGTH [A]____________________________>

210 220 230 240 250 260 270 280 290 300

CGAGCCCGTGGCCAGGTGCATCATCCCCGATCTGATCGGCATGGGCAAGAGCGGCAAGAGCGGCAACGGCAGCTACAGGCTGCTGGACCACTACAAGTAC

GCTCGGGCACCGGTCCACGTAGTAGGGGCTAGACTAGCCGTACCCGTTCTCGCCGTTCTCGCCGTTGCCGTCGATGTCCGACGACCTGGTGATGTTCATG

E P V A R C I I P D L I G M G K S G K S G N G S Y R L L D H Y K Y>

___________________________TRANSLATION OF RLUC8-KRASG12R FULL-LENGTH [A]____________________________>

310 320 330 340 350 360 370 380 390 400

CTGACCGCCTGGTTCGAGCTCCTGAACCTGCCCAAGAAGATCATCTTCGTGGGCCACGACTGGGGCGCCGCCCTGGCCTTCCACTACGCCTACGAGCACC

GACTGGCGGACCAAGCTCGAGGACTTGGACGGGTTCTTCTAGTAGAAGCACCCGGTGCTGACCCCGCGGCGGGACCGGAAGGTGATGCGGATGCTCGTGG

L T A W F E L L N L P K K I I F V G H D W G A A L A F H Y A Y E H>

___________________________TRANSLATION OF RLUC8-KRASG12R FULL-LENGTH [A]____________________________>

410 420 430 440 450 460 470 480 490 500

AGGACAGGATCAAGGCCATCGTGCACATGGAGAGCGTGGTGGACGTGATCGAGAGCTGGGACGAGTGGCCAGACATCGAGGAGGACATCGCCCTGATCAA

TCCTGTCCTAGTTCCGGTAGCACGTGTACCTCTCGCACCACCTGCACTAGCTCTCGACCCTGCTCACCGGTCTGTAGCTCCTCCTGTAGCGGGACTAGTT

Q D R I K A I V H M E S V V D V I E S W D E W P D I E E D I A L I K>

___________________________TRANSLATION OF RLUC8-KRASG12R FULL-LENGTH [A]____________________________>

510 520 530 540 550 560 570 580 590 600

GAGCGAGGAGGGCGAGAAGATGGTGCTGGAGAACAACTTCTTCGTGGAGACCGTGCTGCCCAGCAAGATCATGAGAAAGCTGGAGCCCGAGGAGTTCGCC

CTCGCTCCTCCCGCTCTTCTACCACGACCTCTTGTTGAAGAAGCACCTCTGGCACGACGGGTCGTTCTAGTACTCTTTCGACCTCGGGCTCCTCAAGCGG

S E E G E K M V L E N N F F V E T V L P S K I M R K L E P E E F A>

___________________________TRANSLATION OF RLUC8-KRASG12R FULL-LENGTH [A]____________________________>

610 620 630 640 650 660 670 680 690 700

GCCTACCTGGAGCCCTTCAAGGAGAAGGGCGAGGTGAGAAGACCCACCCTGAGCTGGCCCAGAGAGATCCCCCTGGTGAAGGGCGGCAAGCCCGACGTGG

CGGATGGACCTCGGGAAGTTCCTCTTCCCGCTCCACTCTTCTGGGTGGGACTCGACCGGGTCTCTCTAGGGGGACCACTTCCCGCCGTTCGGGCTGCACC

A Y L E P F K E K G E V R R P T L S W P R E I P L V K G G K P D V>

___________________________TRANSLATION OF RLUC8-KRASG12R FULL-LENGTH [A]____________________________>

710 720 730 740 750 760 770 780 790 800

TGCAGATCGTGAGAAACTACAACGCCTACCTGAGAGCCAGCGACGACCTGCCCAAGCTGTTCATCGAGAGCGACCCCGGCTTCTTCAGCAACGCCATCGT

ACGTCTAGCACTCTTTGATGTTGCGGATGGACTCTCGGTCGCTGCTGGACGGGTTCGACAAGTAGCTCTCGCTGGGGCCGAAGAAGTCGTTGCGGTAGCA

V Q I V R N Y N A Y L R A S D D L P K L F I E S D P G F F S N A I V>

___________________________TRANSLATION OF RLUC8-KRASG12R FULL-LENGTH [A]____________________________>

810 820 830 840 850 860 870 880 890 900

GGAGGGCGCCAAGAAGTTCCCCAACACCGAGTTCGTGAAGGTGAAGGGCCTGCACTTCCTCCAGGAGGACGCCCCCGACGAGATGGGCAAGTACATCAAG

CCTCCCGCGGTTCTTCAAGGGGTTGTGGCTCAAGCACTTCCACTTCCCGGACGTGAAGGAGGTCCTCCTGCGGGGGCTGCTCTACCCGTTCATGTAGTTC

E G A K K F P N T E F V K V K G L H F L Q E D A P D E M G K Y I K>

___________________________TRANSLATION OF RLUC8-KRASG12R FULL-LENGTH [A]____________________________>

910 920 930 940 950 960 970 980 990 1000

AGCTTCGTGGAGAGAGTGCTGAAGAACGAGCAGCTCGAGGGCGGCGGAGGATCTGGGGGCGGAGGAAGTGGGGGAGGGGGCTCTGCGGCCGCTATGACCG

TCGAAGCACCTCTCTCACGACTTCTTGCTCGTCGAGCTCCCGCCGCCTCCTAGACCCCCGCCTCCTTCACCCCCTCCCCCGAGACGCCGGCGATACTGGC

S F V E R V L K N E Q L E G G G G S G G G G S G G G G S A A A M T>

___________________________TRANSLATION OF RLUC8-KRASG12R FULL-LENGTH [A]____________________________>

1010 1020 1030 1040 1050 1060 1070 1080 1090 1100

AATATAAACTTGTGGTAGTTGGAGCTCGTGGCGTAGGCAAGAGTGCCTTGACGATACAGCTAATTCAGAATCATTTTGTGGACGAATATGATCCAACAAT

TTATATTTGAACACCATCAACCTCGAGCACCGCATCCGTTCTCACGGAACTGCTATGTCGATTAAGTCTTAGTAAAACACCTGCTTATACTAGGTTGTTA

E Y K L V V V G A R G V G K S A L T I Q L I Q N H F V D E Y D P T I>

___________________________TRANSLATION OF RLUC8-KRASG12R FULL-LENGTH [A]____________________________>

1110 1120 1130 1140 1150 1160 1170 1180 1190 1200

AGAGGATTCCTACAGGAAGCAAGTAGTAATTGATGGAGAAACCTGTCTCTTGGATATTCTCGACACAGCAGGTCAAGAGGAGTACAGTGCAATGAGGGAC

TCTCCTAAGGATGTCCTTCGTTCATCATTAACTACCTCTTTGGACAGAGAACCTATAAGAGCTGTGTCGTCCAGTTCTCCTCATGTCACGTTACTCCCTG

E D S Y R K Q V V I D G E T C L L D I L D T A G Q E E Y S A M R D>

___________________________TRANSLATION OF RLUC8-KRASG12R FULL-LENGTH [A]____________________________>

1210 1220 1230 1240 1250 1260 1270 1280 1290 1300

CAGTACATGAGGACTGGGGAGGGCTTTCTTTGTGTATTTGCCATAAATAATACTAAATCATTTGAAGATATTCACCATTATAGAGAACAAATTAAAAGAG

GTCATGTACTCCTGACCCCTCCCGAAAGAAACACATAAACGGTATTTATTATGATTTAGTAAACTTCTATAAGTGGTAATATCTCTTGTTTAATTTTCTC

Q Y M R T G E G F L C V F A I N N T K S F E D I H H Y R E Q I K R>

___________________________TRANSLATION OF RLUC8-KRASG12R FULL-LENGTH [A]____________________________>

1310 1320 1330 1340 1350 1360 1370 1380 1390 1400

TTAAGGACTCTGAAGATGTACCTATGGTCCTAGTAGGAAATAAATGTGATTTGCCTTCCAGAACAGTAGACACAAAACAGGCTCAGGACTTAGCAAGAAG

AATTCCTGAGACTTCTACATGGATACCAGGATCATCCTTTATTTACACTAAACGGAAGGTCTTGTCATCTGTGTTTTGTCCGAGTCCTGAATCGTTCTTC

V K D S E D V P M V L V G N K C D L P S R T V D T K Q A Q D L A R S>

___________________________TRANSLATION OF RLUC8-KRASG12R FULL-LENGTH [A]____________________________>

1410 1420 1430 1440 1450 1460 1470 1480 1490 1500

TTATGGAATTCCTTTTATTGAAACATCAGCAAAGACAAGACAGGGTGTTGATGATGCCTTCTATACATTAGTTCGAGAAATTCGAAAACATAAAGAAAAG

AATACCTTAAGGAAAATAACTTTGTAGTCGTTTCTGTTCTGTCCCACAACTACTACGGAAGATATGTAATCAAGCTCTTTAAGCTTTTGTATTTCTTTTC

Y G I P F I E T S A K T R Q G V D D A F Y T L V R E I R K H K E K>

___________________________TRANSLATION OF RLUC8-KRASG12R FULL-LENGTH [A]____________________________>

1510 1520 1530 1540 1550 1560

ATGAGCAAAGATGGTAAAAAGAAGAAAAAGAAGTCAAAGACAAAGTGTGTAATTATGTAA

TACTCGTTTCTACCATTTTTCTTCTTTTTCTTCAGTTTCTGTTTCACACATTAATACATT

M S K D G K K K K K K S K T K C V I M *>

_______TRANSLATION OF RLUC8-KRASG12R FULL-LENGTH [A]________>

Sequence: RLuc8-KRASG12V full-length Range: 1 to 1560

10 20 30 40 50 60 70 80 90 100

ATGACCAGCAAGGTGTACGACCCCGAGCAGAGGAAGAGGATGATCACCGGCCCCCAGTGGTGGGCCAGGTGCAAGCAGATGAACGTGCTGGACAGCTTCA

TACTGGTCGTTCCACATGCTGGGGCTCGTCTCCTTCTCCTACTAGTGGCCGGGGGTCACCACCCGGTCCACGTTCGTCTACTTGCACGACCTGTCGAAGT

M T S K V Y D P E Q R K R M I T G P Q W W A R C K Q M N V L D S F>

___________________________TRANSLATION OF RLUC8-KRASG12V FULL-LENGTH [A]____________________________>

110 120 130 140 150 160 170 180 190 200

TCAACTACTACGACAGCGAGAAGCACGCCGAGAACGCCGTGATCTTCCTGCACGGCAACGCCACTAGCAGCTACCTGTGGAGGCACGTGGTGCCCCACAT

AGTTGATGATGCTGTCGCTCTTCGTGCGGCTCTTGCGGCACTAGAAGGACGTGCCGTTGCGGTGATCGTCGATGGACACCTCCGTGCACCACGGGGTGTA

I N Y Y D S E K H A E N A V I F L H G N A T S S Y L W R H V V P H I>

___________________________TRANSLATION OF RLUC8-KRASG12V FULL-LENGTH [A]____________________________>

210 220 230 240 250 260 270 280 290 300

CGAGCCCGTGGCCAGGTGCATCATCCCCGATCTGATCGGCATGGGCAAGAGCGGCAAGAGCGGCAACGGCAGCTACAGGCTGCTGGACCACTACAAGTAC

GCTCGGGCACCGGTCCACGTAGTAGGGGCTAGACTAGCCGTACCCGTTCTCGCCGTTCTCGCCGTTGCCGTCGATGTCCGACGACCTGGTGATGTTCATG

E P V A R C I I P D L I G M G K S G K S G N G S Y R L L D H Y K Y>

___________________________TRANSLATION OF RLUC8-KRASG12V FULL-LENGTH [A]____________________________>

310 320 330 340 350 360 370 380 390 400

CTGACCGCCTGGTTCGAGCTCCTGAACCTGCCCAAGAAGATCATCTTCGTGGGCCACGACTGGGGCGCCGCCCTGGCCTTCCACTACGCCTACGAGCACC

GACTGGCGGACCAAGCTCGAGGACTTGGACGGGTTCTTCTAGTAGAAGCACCCGGTGCTGACCCCGCGGCGGGACCGGAAGGTGATGCGGATGCTCGTGG

L T A W F E L L N L P K K I I F V G H D W G A A L A F H Y A Y E H>

___________________________TRANSLATION OF RLUC8-KRASG12V FULL-LENGTH [A]____________________________>

410 420 430 440 450 460 470 480 490 500

AGGACAGGATCAAGGCCATCGTGCACATGGAGAGCGTGGTGGACGTGATCGAGAGCTGGGACGAGTGGCCAGACATCGAGGAGGACATCGCCCTGATCAA

TCCTGTCCTAGTTCCGGTAGCACGTGTACCTCTCGCACCACCTGCACTAGCTCTCGACCCTGCTCACCGGTCTGTAGCTCCTCCTGTAGCGGGACTAGTT

Q D R I K A I V H M E S V V D V I E S W D E W P D I E E D I A L I K>

___________________________TRANSLATION OF RLUC8-KRASG12V FULL-LENGTH [A]____________________________>

510 520 530 540 550 560 570 580 590 600

GAGCGAGGAGGGCGAGAAGATGGTGCTGGAGAACAACTTCTTCGTGGAGACCGTGCTGCCCAGCAAGATCATGAGAAAGCTGGAGCCCGAGGAGTTCGCC

CTCGCTCCTCCCGCTCTTCTACCACGACCTCTTGTTGAAGAAGCACCTCTGGCACGACGGGTCGTTCTAGTACTCTTTCGACCTCGGGCTCCTCAAGCGG

S E E G E K M V L E N N F F V E T V L P S K I M R K L E P E E F A>

___________________________TRANSLATION OF RLUC8-KRASG12V FULL-LENGTH [A]____________________________>

610 620 630 640 650 660 670 680 690 700

GCCTACCTGGAGCCCTTCAAGGAGAAGGGCGAGGTGAGAAGACCCACCCTGAGCTGGCCCAGAGAGATCCCCCTGGTGAAGGGCGGCAAGCCCGACGTGG

CGGATGGACCTCGGGAAGTTCCTCTTCCCGCTCCACTCTTCTGGGTGGGACTCGACCGGGTCTCTCTAGGGGGACCACTTCCCGCCGTTCGGGCTGCACC

A Y L E P F K E K G E V R R P T L S W P R E I P L V K G G K P D V>

___________________________TRANSLATION OF RLUC8-KRASG12V FULL-LENGTH [A]____________________________>

710 720 730 740 750 760 770 780 790 800

TGCAGATCGTGAGAAACTACAACGCCTACCTGAGAGCCAGCGACGACCTGCCCAAGCTGTTCATCGAGAGCGACCCCGGCTTCTTCAGCAACGCCATCGT

ACGTCTAGCACTCTTTGATGTTGCGGATGGACTCTCGGTCGCTGCTGGACGGGTTCGACAAGTAGCTCTCGCTGGGGCCGAAGAAGTCGTTGCGGTAGCA

V Q I V R N Y N A Y L R A S D D L P K L F I E S D P G F F S N A I V>

___________________________TRANSLATION OF RLUC8-KRASG12V FULL-LENGTH [A]____________________________>

810 820 830 840 850 860 870 880 890 900

GGAGGGCGCCAAGAAGTTCCCCAACACCGAGTTCGTGAAGGTGAAGGGCCTGCACTTCCTCCAGGAGGACGCCCCCGACGAGATGGGCAAGTACATCAAG

CCTCCCGCGGTTCTTCAAGGGGTTGTGGCTCAAGCACTTCCACTTCCCGGACGTGAAGGAGGTCCTCCTGCGGGGGCTGCTCTACCCGTTCATGTAGTTC

E G A K K F P N T E F V K V K G L H F L Q E D A P D E M G K Y I K>

___________________________TRANSLATION OF RLUC8-KRASG12V FULL-LENGTH [A]____________________________>

910 920 930 940 950 960 970 980 990 1000

AGCTTCGTGGAGAGAGTGCTGAAGAACGAGCAGCTCGAGGGCGGCGGAGGATCTGGGGGCGGAGGAAGTGGGGGAGGGGGCTCTGCGGCCGCTATGACCG

TCGAAGCACCTCTCTCACGACTTCTTGCTCGTCGAGCTCCCGCCGCCTCCTAGACCCCCGCCTCCTTCACCCCCTCCCCCGAGACGCCGGCGATACTGGC

S F V E R V L K N E Q L E G G G G S G G G G S G G G G S A A A M T>

___________________________TRANSLATION OF RLUC8-KRASG12V FULL-LENGTH [A]____________________________>

1010 1020 1030 1040 1050 1060 1070 1080 1090 1100

AATATAAACTTGTGGTAGTTGGAGCTGTTGGCGTAGGCAAGAGTGCCTTGACGATACAGCTAATTCAGAATCATTTTGTGGACGAATATGATCCAACAAT

TTATATTTGAACACCATCAACCTCGACAACCGCATCCGTTCTCACGGAACTGCTATGTCGATTAAGTCTTAGTAAAACACCTGCTTATACTAGGTTGTTA

E Y K L V V V G A V G V G K S A L T I Q L I Q N H F V D E Y D P T I>

___________________________TRANSLATION OF RLUC8-KRASG12V FULL-LENGTH [A]____________________________>

1110 1120 1130 1140 1150 1160 1170 1180 1190 1200

AGAGGATTCCTACAGGAAGCAAGTAGTAATTGATGGAGAAACCTGTCTCTTGGATATTCTCGACACAGCAGGTCAAGAGGAGTACAGTGCAATGAGGGAC

TCTCCTAAGGATGTCCTTCGTTCATCATTAACTACCTCTTTGGACAGAGAACCTATAAGAGCTGTGTCGTCCAGTTCTCCTCATGTCACGTTACTCCCTG

E D S Y R K Q V V I D G E T C L L D I L D T A G Q E E Y S A M R D>

___________________________TRANSLATION OF RLUC8-KRASG12V FULL-LENGTH [A]____________________________>

1210 1220 1230 1240 1250 1260 1270 1280 1290 1300

CAGTACATGAGGACTGGGGAGGGCTTTCTTTGTGTATTTGCCATAAATAATACTAAATCATTTGAAGATATTCATCATTATAGAGAACAAATTAAAAGAG

GTCATGTACTCCTGACCCCTCCCGAAAGAAACACATAAACGGTATTTATTATGATTTAGTAAACTTCTATAAGTAGTAATATCTCTTGTTTAATTTTCTC

Q Y M R T G E G F L C V F A I N N T K S F E D I H H Y R E Q I K R>

___________________________TRANSLATION OF RLUC8-KRASG12V FULL-LENGTH [A]____________________________>

1310 1320 1330 1340 1350 1360 1370 1380 1390 1400

TTAAGGACTCTGAAGATGTACCTATGGTCCTAGTAGGAAATAAATGTGATTTGCCTTCCAGAACAGTAGACACAAAACAGGCTCAGGACTTAGCAAGAAG

AATTCCTGAGACTTCTACATGGATACCAGGATCATCCTTTATTTACACTAAACGGAAGGTCTTGTCATCTGTGTTTTGTCCGAGTCCTGAATCGTTCTTC

V K D S E D V P M V L V G N K C D L P S R T V D T K Q A Q D L A R S>

___________________________TRANSLATION OF RLUC8-KRASG12V FULL-LENGTH [A]____________________________>

1410 1420 1430 1440 1450 1460 1470 1480 1490 1500

TTATGGAATTCCTTTTATTGAAACATCAGCAAAGACAAGACAGGGTGTTGATGATGCCTTCTATACATTAGTTCGAGAAATTCGAAAACATAAAGAAAAG

AATACCTTAAGGAAAATAACTTTGTAGTCGTTTCTGTTCTGTCCCACAACTACTACGGAAGATATGTAATCAAGCTCTTTAAGCTTTTGTATTTCTTTTC

Y G I P F I E T S A K T R Q G V D D A F Y T L V R E I R K H K E K>

___________________________TRANSLATION OF RLUC8-KRASG12V FULL-LENGTH [A]____________________________>

1510 1520 1530 1540 1550 1560

ATGAGCAAAGATGGTAAAAAGAAGAAAAAGAAGTCAAAGACAAAGTGTGTAATTATGTAA

TACTCGTTTCTACCATTTTTCTTCTTTTTCTTCAGTTTCTGTTTCACACATTAATACATT

M S K D G K K K K K K S K T K C V I M *>

_______TRANSLATION OF RLUC8-KRASG12V FULL-LENGTH [A]________>

Sequence: RLuc8-KRASS17N full-length Range: 1 to 1560

10 20 30 40 50 60 70 80 90 100

ATGACCAGCAAGGTGTACGACCCCGAGCAGAGGAAGAGGATGATCACCGGCCCCCAGTGGTGGGCCAGGTGCAAGCAGATGAACGTGCTGGACAGCTTCA

TACTGGTCGTTCCACATGCTGGGGCTCGTCTCCTTCTCCTACTAGTGGCCGGGGGTCACCACCCGGTCCACGTTCGTCTACTTGCACGACCTGTCGAAGT

M T S K V Y D P E Q R K R M I T G P Q W W A R C K Q M N V L D S F>

___________________________TRANSLATION OF RLUC8-KRASS17N FULL-LENGTH [A]____________________________>

110 120 130 140 150 160 170 180 190 200

TCAACTACTACGACAGCGAGAAGCACGCCGAGAACGCCGTGATCTTCCTGCACGGCAACGCCACTAGCAGCTACCTGTGGAGGCACGTGGTGCCCCACAT

AGTTGATGATGCTGTCGCTCTTCGTGCGGCTCTTGCGGCACTAGAAGGACGTGCCGTTGCGGTGATCGTCGATGGACACCTCCGTGCACCACGGGGTGTA

I N Y Y D S E K H A E N A V I F L H G N A T S S Y L W R H V V P H I>

___________________________TRANSLATION OF RLUC8-KRASS17N FULL-LENGTH [A]____________________________>

210 220 230 240 250 260 270 280 290 300

CGAGCCCGTGGCCAGGTGCATCATCCCCGATCTGATCGGCATGGGCAAGAGCGGCAAGAGCGGCAACGGCAGCTACAGGCTGCTGGACCACTACAAGTAC

GCTCGGGCACCGGTCCACGTAGTAGGGGCTAGACTAGCCGTACCCGTTCTCGCCGTTCTCGCCGTTGCCGTCGATGTCCGACGACCTGGTGATGTTCATG

E P V A R C I I P D L I G M G K S G K S G N G S Y R L L D H Y K Y>

___________________________TRANSLATION OF RLUC8-KRASS17N FULL-LENGTH [A]____________________________>

310 320 330 340 350 360 370 380 390 400

CTGACCGCCTGGTTCGAGCTCCTGAACCTGCCCAAGAAGATCATCTTCGTGGGCCACGACTGGGGCGCCGCCCTGGCCTTCCACTACGCCTACGAGCACC

GACTGGCGGACCAAGCTCGAGGACTTGGACGGGTTCTTCTAGTAGAAGCACCCGGTGCTGACCCCGCGGCGGGACCGGAAGGTGATGCGGATGCTCGTGG

L T A W F E L L N L P K K I I F V G H D W G A A L A F H Y A Y E H>

___________________________TRANSLATION OF RLUC8-KRASS17N FULL-LENGTH [A]____________________________>

410 420 430 440 450 460 470 480 490 500

AGGACAGGATCAAGGCCATCGTGCACATGGAGAGCGTGGTGGACGTGATCGAGAGCTGGGACGAGTGGCCAGACATCGAGGAGGACATCGCCCTGATCAA

TCCTGTCCTAGTTCCGGTAGCACGTGTACCTCTCGCACCACCTGCACTAGCTCTCGACCCTGCTCACCGGTCTGTAGCTCCTCCTGTAGCGGGACTAGTT

Q D R I K A I V H M E S V V D V I E S W D E W P D I E E D I A L I K>

___________________________TRANSLATION OF RLUC8-KRASS17N FULL-LENGTH [A]____________________________>

510 520 530 540 550 560 570 580 590 600

GAGCGAGGAGGGCGAGAAGATGGTGCTGGAGAACAACTTCTTCGTGGAGACCGTGCTGCCCAGCAAGATCATGAGAAAGCTGGAGCCCGAGGAGTTCGCC

CTCGCTCCTCCCGCTCTTCTACCACGACCTCTTGTTGAAGAAGCACCTCTGGCACGACGGGTCGTTCTAGTACTCTTTCGACCTCGGGCTCCTCAAGCGG

S E E G E K M V L E N N F F V E T V L P S K I M R K L E P E E F A>

___________________________TRANSLATION OF RLUC8-KRASS17N FULL-LENGTH [A]____________________________>

610 620 630 640 650 660 670 680 690 700

GCCTACCTGGAGCCCTTCAAGGAGAAGGGCGAGGTGAGAAGACCCACCCTGAGCTGGCCCAGAGAGATCCCCCTGGTGAAGGGCGGCAAGCCCGACGTGG

CGGATGGACCTCGGGAAGTTCCTCTTCCCGCTCCACTCTTCTGGGTGGGACTCGACCGGGTCTCTCTAGGGGGACCACTTCCCGCCGTTCGGGCTGCACC

A Y L E P F K E K G E V R R P T L S W P R E I P L V K G G K P D V>

___________________________TRANSLATION OF RLUC8-KRASS17N FULL-LENGTH [A]____________________________>

710 720 730 740 750 760 770 780 790 800

TGCAGATCGTGAGAAACTACAACGCCTACCTGAGAGCCAGCGACGACCTGCCCAAGCTGTTCATCGAGAGCGACCCCGGCTTCTTCAGCAACGCCATCGT

ACGTCTAGCACTCTTTGATGTTGCGGATGGACTCTCGGTCGCTGCTGGACGGGTTCGACAAGTAGCTCTCGCTGGGGCCGAAGAAGTCGTTGCGGTAGCA

V Q I V R N Y N A Y L R A S D D L P K L F I E S D P G F F S N A I V>

___________________________TRANSLATION OF RLUC8-KRASS17N FULL-LENGTH [A]____________________________>

810 820 830 840 850 860 870 880 890 900

GGAGGGCGCCAAGAAGTTCCCCAACACCGAGTTCGTGAAGGTGAAGGGCCTGCACTTCCTCCAGGAGGACGCCCCCGACGAGATGGGCAAGTACATCAAG

CCTCCCGCGGTTCTTCAAGGGGTTGTGGCTCAAGCACTTCCACTTCCCGGACGTGAAGGAGGTCCTCCTGCGGGGGCTGCTCTACCCGTTCATGTAGTTC

E G A K K F P N T E F V K V K G L H F L Q E D A P D E M G K Y I K>

___________________________TRANSLATION OF RLUC8-KRASS17N FULL-LENGTH [A]____________________________>

910 920 930 940 950 960 970 980 990 1000

AGCTTCGTGGAGAGAGTGCTGAAGAACGAGCAGCTCGAGGGCGGCGGAGGATCTGGGGGCGGAGGAAGTGGGGGAGGGGGCTCTGCGGCCGCTATGACCG

TCGAAGCACCTCTCTCACGACTTCTTGCTCGTCGAGCTCCCGCCGCCTCCTAGACCCCCGCCTCCTTCACCCCCTCCCCCGAGACGCCGGCGATACTGGC

S F V E R V L K N E Q L E G G G G S G G G G S G G G G S A A A M T>

___________________________TRANSLATION OF RLUC8-KRASS17N FULL-LENGTH [A]____________________________>

1010 1020 1030 1040 1050 1060 1070 1080 1090 1100

AATATAAACTTGTGGTAGTTGGAGCTGGTGGCGTAGGCAAGAACGCCTTGACGATACAGCTAATTCAGAATCATTTTGTGGACGAATATGATCCAACAAT

TTATATTTGAACACCATCAACCTCGACCACCGCATCCGTTCTTGCGGAACTGCTATGTCGATTAAGTCTTAGTAAAACACCTGCTTATACTAGGTTGTTA

E Y K L V V V G A G G V G K N A L T I Q L I Q N H F V D E Y D P T I>

___________________________TRANSLATION OF RLUC8-KRASS17N FULL-LENGTH [A]____________________________>

1110 1120 1130 1140 1150 1160 1170 1180 1190 1200

AGAGGATTCCTACAGGAAGCAAGTAGTAATTGATGGAGAAACCTGTCTCTTGGATATTCTCGACACAGCAGGTCAAGAGGAGTACAGTGCAATGAGGGAC

TCTCCTAAGGATGTCCTTCGTTCATCATTAACTACCTCTTTGGACAGAGAACCTATAAGAGCTGTGTCGTCCAGTTCTCCTCATGTCACGTTACTCCCTG

E D S Y R K Q V V I D G E T C L L D I L D T A G Q E E Y S A M R D>

___________________________TRANSLATION OF RLUC8-KRASS17N FULL-LENGTH [A]____________________________>

1210 1220 1230 1240 1250 1260 1270 1280 1290 1300

CAGTACATGAGGACTGGGGAGGGCTTTCTTTGTGTATTTGCCATAAATAATACTAAATCATTTGAAGATATTCACCATTATAGAGAACAAATTAAAAGAG

GTCATGTACTCCTGACCCCTCCCGAAAGAAACACATAAACGGTATTTATTATGATTTAGTAAACTTCTATAAGTGGTAATATCTCTTGTTTAATTTTCTC

Q Y M R T G E G F L C V F A I N N T K S F E D I H H Y R E Q I K R>

___________________________TRANSLATION OF RLUC8-KRASS17N FULL-LENGTH [A]____________________________>

1310 1320 1330 1340 1350 1360 1370 1380 1390 1400

TTAAGGACTCTGAAGATGTACCTATGGTCCTAGTAGGAAATAAATGTGATTTGCCTTCCAGAACAGTAGACACAAAACAGGCTCAGGACTTAGCAAGAAG

AATTCCTGAGACTTCTACATGGATACCAGGATCATCCTTTATTTACACTAAACGGAAGGTCTTGTCATCTGTGTTTTGTCCGAGTCCTGAATCGTTCTTC

V K D S E D V P M V L V G N K C D L P S R T V D T K Q A Q D L A R S>

___________________________TRANSLATION OF RLUC8-KRASS17N FULL-LENGTH [A]____________________________>

1410 1420 1430 1440 1450 1460 1470 1480 1490 1500

TTATGGAATTCCTTTTATTGAAACATCAGCAAAGACAAGACAGGGTGTTGATGATGCCTTCTATACATTAGTTCGAGAAATTCGAAAACATAAAGAAAAG

AATACCTTAAGGAAAATAACTTTGTAGTCGTTTCTGTTCTGTCCCACAACTACTACGGAAGATATGTAATCAAGCTCTTTAAGCTTTTGTATTTCTTTTC

Y G I P F I E T S A K T R Q G V D D A F Y T L V R E I R K H K E K>

___________________________TRANSLATION OF RLUC8-KRASS17N FULL-LENGTH [A]____________________________>

1510 1520 1530 1540 1550 1560

ATGAGCAAAGATGGTAAAAAGAAGAAAAAGAAGTCAAAGACAAAGTGTGTAATTATGTAA

TACTCGTTTCTACCATTTTTCTTCTTTTTCTTCAGTTTCTGTTTCACACATTAATACATT

M S K D G K K K K K K S K T K C V I M *>

_______TRANSLATION OF RLUC8-KRASS17N FULL-LENGTH [A]________>

Sequence: RLuc8-KRASWT full-length Range: 1 to 1560

10 20 30 40 50 60 70 80 90 100

ATGACCAGCAAGGTGTACGACCCCGAGCAGAGGAAGAGGATGATCACCGGCCCCCAGTGGTGGGCCAGGTGCAAGCAGATGAACGTGCTGGACAGCTTCA

TACTGGTCGTTCCACATGCTGGGGCTCGTCTCCTTCTCCTACTAGTGGCCGGGGGTCACCACCCGGTCCACGTTCGTCTACTTGCACGACCTGTCGAAGT

M T S K V Y D P E Q R K R M I T G P Q W W A R C K Q M N V L D S F>

____________________________TRANSLATION OF RLUC8-KRASWT FULL-LENGTH [A]_____________________________>

110 120 130 140 150 160 170 180 190 200

TCAACTACTACGACAGCGAGAAGCACGCCGAGAACGCCGTGATCTTCCTGCACGGCAACGCCACTAGCAGCTACCTGTGGAGGCACGTGGTGCCCCACAT

AGTTGATGATGCTGTCGCTCTTCGTGCGGCTCTTGCGGCACTAGAAGGACGTGCCGTTGCGGTGATCGTCGATGGACACCTCCGTGCACCACGGGGTGTA

I N Y Y D S E K H A E N A V I F L H G N A T S S Y L W R H V V P H I>

____________________________TRANSLATION OF RLUC8-KRASWT FULL-LENGTH [A]_____________________________>

210 220 230 240 250 260 270 280 290 300

CGAGCCCGTGGCCAGGTGCATCATCCCCGATCTGATCGGCATGGGCAAGAGCGGCAAGAGCGGCAACGGCAGCTACAGGCTGCTGGACCACTACAAGTAC

GCTCGGGCACCGGTCCACGTAGTAGGGGCTAGACTAGCCGTACCCGTTCTCGCCGTTCTCGCCGTTGCCGTCGATGTCCGACGACCTGGTGATGTTCATG

E P V A R C I I P D L I G M G K S G K S G N G S Y R L L D H Y K Y>

____________________________TRANSLATION OF RLUC8-KRASWT FULL-LENGTH [A]_____________________________>

310 320 330 340 350 360 370 380 390 400

CTGACCGCCTGGTTCGAGCTCCTGAACCTGCCCAAGAAGATCATCTTCGTGGGCCACGACTGGGGCGCCGCCCTGGCCTTCCACTACGCCTACGAGCACC

GACTGGCGGACCAAGCTCGAGGACTTGGACGGGTTCTTCTAGTAGAAGCACCCGGTGCTGACCCCGCGGCGGGACCGGAAGGTGATGCGGATGCTCGTGG

L T A W F E L L N L P K K I I F V G H D W G A A L A F H Y A Y E H>

____________________________TRANSLATION OF RLUC8-KRASWT FULL-LENGTH [A]_____________________________>

410 420 430 440 450 460 470 480 490 500

AGGACAGGATCAAGGCCATCGTGCACATGGAGAGCGTGGTGGACGTGATCGAGAGCTGGGACGAGTGGCCAGACATCGAGGAGGACATCGCCCTGATCAA

TCCTGTCCTAGTTCCGGTAGCACGTGTACCTCTCGCACCACCTGCACTAGCTCTCGACCCTGCTCACCGGTCTGTAGCTCCTCCTGTAGCGGGACTAGTT

Q D R I K A I V H M E S V V D V I E S W D E W P D I E E D I A L I K>

____________________________TRANSLATION OF RLUC8-KRASWT FULL-LENGTH [A]_____________________________>

510 520 530 540 550 560 570 580 590 600

GAGCGAGGAGGGCGAGAAGATGGTGCTGGAGAACAACTTCTTCGTGGAGACCGTGCTGCCCAGCAAGATCATGAGAAAGCTGGAGCCCGAGGAGTTCGCC

CTCGCTCCTCCCGCTCTTCTACCACGACCTCTTGTTGAAGAAGCACCTCTGGCACGACGGGTCGTTCTAGTACTCTTTCGACCTCGGGCTCCTCAAGCGG

S E E G E K M V L E N N F F V E T V L P S K I M R K L E P E E F A>

____________________________TRANSLATION OF RLUC8-KRASWT FULL-LENGTH [A]_____________________________>

610 620 630 640 650 660 670 680 690 700

GCCTACCTGGAGCCCTTCAAGGAGAAGGGCGAGGTGAGAAGACCCACCCTGAGCTGGCCCAGAGAGATCCCCCTGGTGAAGGGCGGCAAGCCCGACGTGG

CGGATGGACCTCGGGAAGTTCCTCTTCCCGCTCCACTCTTCTGGGTGGGACTCGACCGGGTCTCTCTAGGGGGACCACTTCCCGCCGTTCGGGCTGCACC

A Y L E P F K E K G E V R R P T L S W P R E I P L V K G G K P D V>

____________________________TRANSLATION OF RLUC8-KRASWT FULL-LENGTH [A]_____________________________>

710 720 730 740 750 760 770 780 790 800

TGCAGATCGTGAGAAACTACAACGCCTACCTGAGAGCCAGCGACGACCTGCCCAAGCTGTTCATCGAGAGCGACCCCGGCTTCTTCAGCAACGCCATCGT

ACGTCTAGCACTCTTTGATGTTGCGGATGGACTCTCGGTCGCTGCTGGACGGGTTCGACAAGTAGCTCTCGCTGGGGCCGAAGAAGTCGTTGCGGTAGCA

V Q I V R N Y N A Y L R A S D D L P K L F I E S D P G F F S N A I V>

____________________________TRANSLATION OF RLUC8-KRASWT FULL-LENGTH [A]_____________________________>

810 820 830 840 850 860 870 880 890 900

GGAGGGCGCCAAGAAGTTCCCCAACACCGAGTTCGTGAAGGTGAAGGGCCTGCACTTCCTCCAGGAGGACGCCCCCGACGAGATGGGCAAGTACATCAAG

CCTCCCGCGGTTCTTCAAGGGGTTGTGGCTCAAGCACTTCCACTTCCCGGACGTGAAGGAGGTCCTCCTGCGGGGGCTGCTCTACCCGTTCATGTAGTTC

E G A K K F P N T E F V K V K G L H F L Q E D A P D E M G K Y I K>

____________________________TRANSLATION OF RLUC8-KRASWT FULL-LENGTH [A]_____________________________>

910 920 930 940 950 960 970 980 990 1000

AGCTTCGTGGAGAGAGTGCTGAAGAACGAGCAGCTCGAGGGCGGCGGAGGATCTGGGGGCGGAGGAAGTGGGGGAGGGGGCTCTGCGGCCGCTATGACCG

TCGAAGCACCTCTCTCACGACTTCTTGCTCGTCGAGCTCCCGCCGCCTCCTAGACCCCCGCCTCCTTCACCCCCTCCCCCGAGACGCCGGCGATACTGGC

S F V E R V L K N E Q L E G G G G S G G G G S G G G G S A A A M T>

____________________________TRANSLATION OF RLUC8-KRASWT FULL-LENGTH [A]_____________________________>

1010 1020 1030 1040 1050 1060 1070 1080 1090 1100

AATATAAACTTGTGGTAGTTGGAGCTGGTGGCGTAGGCAAGAGTGCCTTGACGATACAGCTAATTCAGAATCATTTTGTGGACGAATATGATCCAACAAT

TTATATTTGAACACCATCAACCTCGACCACCGCATCCGTTCTCACGGAACTGCTATGTCGATTAAGTCTTAGTAAAACACCTGCTTATACTAGGTTGTTA

E Y K L V V V G A G G V G K S A L T I Q L I Q N H F V D E Y D P T I>

____________________________TRANSLATION OF RLUC8-KRASWT FULL-LENGTH [A]_____________________________>

1110 1120 1130 1140 1150 1160 1170 1180 1190 1200

AGAGGATTCCTACAGGAAGCAAGTAGTAATTGATGGAGAAACCTGTCTCTTGGATATTCTCGACACAGCAGGTCAAGAGGAGTACAGTGCAATGAGGGAC

TCTCCTAAGGATGTCCTTCGTTCATCATTAACTACCTCTTTGGACAGAGAACCTATAAGAGCTGTGTCGTCCAGTTCTCCTCATGTCACGTTACTCCCTG

E D S Y R K Q V V I D G E T C L L D I L D T A G Q E E Y S A M R D>

____________________________TRANSLATION OF RLUC8-KRASWT FULL-LENGTH [A]_____________________________>

1210 1220 1230 1240 1250 1260 1270 1280 1290 1300

CAGTACATGAGGACTGGGGAGGGCTTTCTTTGTGTATTTGCCATAAATAATACTAAATCATTTGAAGATATTCACCATTATAGAGAACAAATTAAAAGAG

GTCATGTACTCCTGACCCCTCCCGAAAGAAACACATAAACGGTATTTATTATGATTTAGTAAACTTCTATAAGTGGTAATATCTCTTGTTTAATTTTCTC

Q Y M R T G E G F L C V F A I N N T K S F E D I H H Y R E Q I K R>

____________________________TRANSLATION OF RLUC8-KRASWT FULL-LENGTH [A]_____________________________>

1310 1320 1330 1340 1350 1360 1370 1380 1390 1400

TTAAGGACTCTGAAGATGTACCTATGGTCCTAGTAGGAAATAAATGTGATTTGCCTTCCAGAACAGTAGACACAAAACAGGCTCAGGACTTAGCAAGAAG

AATTCCTGAGACTTCTACATGGATACCAGGATCATCCTTTATTTACACTAAACGGAAGGTCTTGTCATCTGTGTTTTGTCCGAGTCCTGAATCGTTCTTC

V K D S E D V P M V L V G N K C D L P S R T V D T K Q A Q D L A R S>

____________________________TRANSLATION OF RLUC8-KRASWT FULL-LENGTH [A]_____________________________>

1410 1420 1430 1440 1450 1460 1470 1480 1490 1500

TTATGGAATTCCTTTTATTGAAACATCAGCAAAGACAAGACAGGGTGTTGATGATGCCTTCTATACATTAGTTCGAGAAATTCGAAAACATAAAGAAAAG

AATACCTTAAGGAAAATAACTTTGTAGTCGTTTCTGTTCTGTCCCACAACTACTACGGAAGATATGTAATCAAGCTCTTTAAGCTTTTGTATTTCTTTTC

Y G I P F I E T S A K T R Q G V D D A F Y T L V R E I R K H K E K>

____________________________TRANSLATION OF RLUC8-KRASWT FULL-LENGTH [A]_____________________________>

1510 1520 1530 1540 1550 1560

ATGAGCAAAGATGGTAAAAAGAAGAAAAAGAAGTCAAAGACAAAGTGTGTAATTATGTAA

TACTCGTTTCTACCATTTTTCTTCTTTTTCTTCAGTTTCTGTTTCACACATTAATACATT

M S K D G K K K K K K S K T K C V I M *>

________TRANSLATION OF RLUC8-KRASWT FULL-LENGTH [A]_________>

Sequence: RLuc8-HRASG12V full-length Range: 1 to 1563

10 20 30 40 50 60 70 80 90 100

ATGACCAGCAAGGTGTACGACCCCGAGCAGAGGAAGAGGATGATCACCGGCCCCCAGTGGTGGGCCAGGTGCAAGCAGATGAACGTGCTGGACAGCTTCA

TACTGGTCGTTCCACATGCTGGGGCTCGTCTCCTTCTCCTACTAGTGGCCGGGGGTCACCACCCGGTCCACGTTCGTCTACTTGCACGACCTGTCGAAGT

M T S K V Y D P E Q R K R M I T G P Q W W A R C K Q M N V L D S F>

___________________________TRANSLATION OF RLUC8-HRASG12V FULL-LENGTH [A]____________________________>

110 120 130 140 150 160 170 180 190 200

TCAACTACTACGACAGCGAGAAGCACGCCGAGAACGCCGTGATCTTCCTGCACGGCAACGCCACTAGCAGCTACCTGTGGAGGCACGTGGTGCCCCACAT

AGTTGATGATGCTGTCGCTCTTCGTGCGGCTCTTGCGGCACTAGAAGGACGTGCCGTTGCGGTGATCGTCGATGGACACCTCCGTGCACCACGGGGTGTA

I N Y Y D S E K H A E N A V I F L H G N A T S S Y L W R H V V P H I>

___________________________TRANSLATION OF RLUC8-HRASG12V FULL-LENGTH [A]____________________________>

210 220 230 240 250 260 270 280 290 300

CGAGCCCGTGGCCAGGTGCATCATCCCCGATCTGATCGGCATGGGCAAGAGCGGCAAGAGCGGCAACGGCAGCTACAGGCTGCTGGACCACTACAAGTAC

GCTCGGGCACCGGTCCACGTAGTAGGGGCTAGACTAGCCGTACCCGTTCTCGCCGTTCTCGCCGTTGCCGTCGATGTCCGACGACCTGGTGATGTTCATG

E P V A R C I I P D L I G M G K S G K S G N G S Y R L L D H Y K Y>

___________________________TRANSLATION OF RLUC8-HRASG12V FULL-LENGTH [A]____________________________>

310 320 330 340 350 360 370 380 390 400

CTGACCGCCTGGTTCGAGCTCCTGAACCTGCCCAAGAAGATCATCTTCGTGGGCCACGACTGGGGCGCCGCCCTGGCCTTCCACTACGCCTACGAGCACC

GACTGGCGGACCAAGCTCGAGGACTTGGACGGGTTCTTCTAGTAGAAGCACCCGGTGCTGACCCCGCGGCGGGACCGGAAGGTGATGCGGATGCTCGTGG

L T A W F E L L N L P K K I I F V G H D W G A A L A F H Y A Y E H>

___________________________TRANSLATION OF RLUC8-HRASG12V FULL-LENGTH [A]____________________________>

410 420 430 440 450 460 470 480 490 500

AGGACAGGATCAAGGCCATCGTGCACATGGAGAGCGTGGTGGACGTGATCGAGAGCTGGGACGAGTGGCCAGACATCGAGGAGGACATCGCCCTGATCAA

TCCTGTCCTAGTTCCGGTAGCACGTGTACCTCTCGCACCACCTGCACTAGCTCTCGACCCTGCTCACCGGTCTGTAGCTCCTCCTGTAGCGGGACTAGTT

Q D R I K A I V H M E S V V D V I E S W D E W P D I E E D I A L I K>

___________________________TRANSLATION OF RLUC8-HRASG12V FULL-LENGTH [A]____________________________>

510 520 530 540 550 560 570 580 590 600

GAGCGAGGAGGGCGAGAAGATGGTGCTGGAGAACAACTTCTTCGTGGAGACCGTGCTGCCCAGCAAGATCATGAGAAAGCTGGAGCCCGAGGAGTTCGCC

CTCGCTCCTCCCGCTCTTCTACCACGACCTCTTGTTGAAGAAGCACCTCTGGCACGACGGGTCGTTCTAGTACTCTTTCGACCTCGGGCTCCTCAAGCGG

S E E G E K M V L E N N F F V E T V L P S K I M R K L E P E E F A>

___________________________TRANSLATION OF RLUC8-HRASG12V FULL-LENGTH [A]____________________________>

610 620 630 640 650 660 670 680 690 700

GCCTACCTGGAGCCCTTCAAGGAGAAGGGCGAGGTGAGAAGACCCACCCTGAGCTGGCCCAGAGAGATCCCCCTGGTGAAGGGCGGCAAGCCCGACGTGG

CGGATGGACCTCGGGAAGTTCCTCTTCCCGCTCCACTCTTCTGGGTGGGACTCGACCGGGTCTCTCTAGGGGGACCACTTCCCGCCGTTCGGGCTGCACC

A Y L E P F K E K G E V R R P T L S W P R E I P L V K G G K P D V>

___________________________TRANSLATION OF RLUC8-HRASG12V FULL-LENGTH [A]____________________________>

710 720 730 740 750 760 770 780 790 800

TGCAGATCGTGAGAAACTACAACGCCTACCTGAGAGCCAGCGACGACCTGCCCAAGCTGTTCATCGAGAGCGACCCCGGCTTCTTCAGCAACGCCATCGT

ACGTCTAGCACTCTTTGATGTTGCGGATGGACTCTCGGTCGCTGCTGGACGGGTTCGACAAGTAGCTCTCGCTGGGGCCGAAGAAGTCGTTGCGGTAGCA

V Q I V R N Y N A Y L R A S D D L P K L F I E S D P G F F S N A I V>

___________________________TRANSLATION OF RLUC8-HRASG12V FULL-LENGTH [A]____________________________>

810 820 830 840 850 860 870 880 890 900

GGAGGGCGCCAAGAAGTTCCCCAACACCGAGTTCGTGAAGGTGAAGGGCCTGCACTTCCTCCAGGAGGACGCCCCCGACGAGATGGGCAAGTACATCAAG

CCTCCCGCGGTTCTTCAAGGGGTTGTGGCTCAAGCACTTCCACTTCCCGGACGTGAAGGAGGTCCTCCTGCGGGGGCTGCTCTACCCGTTCATGTAGTTC

E G A K K F P N T E F V K V K G L H F L Q E D A P D E M G K Y I K>

___________________________TRANSLATION OF RLUC8-HRASG12V FULL-LENGTH [A]____________________________>

910 920 930 940 950 960 970 980 990 1000

AGCTTCGTGGAGAGAGTGCTGAAGAACGAGCAGCTCGAGGGCGGCGGAGGATCTGGGGGCGGAGGAAGTGGGGGAGGGGGCTCTGCGGCCGCTATGACCG

TCGAAGCACCTCTCTCACGACTTCTTGCTCGTCGAGCTCCCGCCGCCTCCTAGACCCCCGCCTCCTTCACCCCCTCCCCCGAGACGCCGGCGATACTGGC

S F V E R V L K N E Q L E G G G G S G G G G S G G G G S A A A M T>

___________________________TRANSLATION OF RLUC8-HRASG12V FULL-LENGTH [A]____________________________>

1010 1020 1030 1040 1050 1060 1070 1080 1090 1100

AATACAAGCTTGTTGTTGTTGGCGCCGTCGGTGTGGGCAAGAGTGCGCTGACCATCCAGCTGATCCAGAACCATTTTGTGGACGAATACGACCCCACTAT

TTATGTTCGAACAACAACAACCGCGGCAGCCACACCCGTTCTCACGCGACTGGTAGGTCGACTAGGTCTTGGTAAAACACCTGCTTATGCTGGGGTGATA

E Y K L V V V G A V G V G K S A L T I Q L I Q N H F V D E Y D P T I>

___________________________TRANSLATION OF RLUC8-HRASG12V FULL-LENGTH [A]____________________________>

1110 1120 1130 1140 1150 1160 1170 1180 1190 1200

AGAGGATTCCTACCGGAAGCAGGTGGTCATTGATGGGGAGACGTGCCTGTTGGACATCCTGGATACCGCCGGCCAGGAGGAGTACAGCGCCATGCGGGAC

TCTCCTAAGGATGGCCTTCGTCCACCAGTAACTACCCCTCTGCACGGACAACCTGTAGGACCTATGGCGGCCGGTCCTCCTCATGTCGCGGTACGCCCTG

E D S Y R K Q V V I D G E T C L L D I L D T A G Q E E Y S A M R D>

___________________________TRANSLATION OF RLUC8-HRASG12V FULL-LENGTH [A]____________________________>

1210 1220 1230 1240 1250 1260 1270 1280 1290 1300

CAGTACATGCGCACCGGGGAGGGCTTCCTGTGTGTGTTTGCCATCAACAACACCAAGTCTTTTGAGGACATCCACCAGTACAGGGAGCAGATCAAACGGG

GTCATGTACGCGTGGCCCCTCCCGAAGGACACACACAAACGGTAGTTGTTGTGGTTCAGAAAACTCCTGTAGGTGGTCATGTCCCTCGTCTAGTTTGCCC

Q Y M R T G E G F L C V F A I N N T K S F E D I H Q Y R E Q I K R>

___________________________TRANSLATION OF RLUC8-HRASG12V FULL-LENGTH [A]____________________________>

1310 1320 1330 1340 1350 1360 1370 1380 1390 1400

TGAAGGACTCGGATGACGTGCCCATGGTGCTGGTGGGGAACAAGTGTGACCTGGCTGCACGCACTGTGGAATCTCGGCAGGCTCAGGACCTCGCCCGAAG

ACTTCCTGAGCCTACTGCACGGGTACCACGACCACCCCTTGTTCACACTGGACCGACGTGCGTGACACCTTAGAGCCGTCCGAGTCCTGGAGCGGGCTTC

V K D S D D V P M V L V G N K C D L A A R T V E S R Q A Q D L A R S>

___________________________TRANSLATION OF RLUC8-HRASG12V FULL-LENGTH [A]____________________________>

1410 1420 1430 1440 1450 1460 1470 1480 1490 1500

CTACGGCATCCCCTACATCGAGACCTCGGCCAAGACCCGGCAGGGAGTGGAGGATGCCTTCTACACGTTGGTGCGTGAGATCCGGCAGCACAAGCTGCGG

GATGCCGTAGGGGATGTAGCTCTGGAGCCGGTTCTGGGCCGTCCCTCACCTCCTACGGAAGATGTGCAACCACGCACTCTAGGCCGTCGTGTTCGACGCC

Y G I P Y I E T S A K T R Q G V E D A F Y T L V R E I R Q H K L R>

___________________________TRANSLATION OF RLUC8-HRASG12V FULL-LENGTH [A]____________________________>

1510 1520 1530 1540 1550 1560

AAGCTGAACCCTCCTGATGAGAGTGGCCCCGGCTGCATGAGCTGCAAGTGTGTGCTCTCCTGA

TTCGACTTGGGAGGACTACTCTCACCGGGGCCGACGTACTCGACGTTCACACACGAGAGGACT

K L N P P D E S G P G C M S C K C V L S *>

________TRANSLATION OF RLUC8-HRASG12V FULL-LENGTH [A]__________>

Sequence: RLuc8-NRASQ61H full-length Range: 1 to 1563

10 20 30 40 50 60 70 80 90 100

ATGACCAGCAAGGTGTACGACCCCGAGCAGAGGAAGAGGATGATCACCGGCCCCCAGTGGTGGGCCAGGTGCAAGCAGATGAACGTGCTGGACAGCTTCA

TACTGGTCGTTCCACATGCTGGGGCTCGTCTCCTTCTCCTACTAGTGGCCGGGGGTCACCACCCGGTCCACGTTCGTCTACTTGCACGACCTGTCGAAGT

M T S K V Y D P E Q R K R M I T G P Q W W A R C K Q M N V L D S F>

___________________________TRANSLATION OF RLUC8-NRASQ61H FULL-LENGTH [A]____________________________>

110 120 130 140 150 160 170 180 190 200

TCAACTACTACGACAGCGAGAAGCACGCCGAGAACGCCGTGATCTTCCTGCACGGCAACGCCACTAGCAGCTACCTGTGGAGGCACGTGGTGCCCCACAT

AGTTGATGATGCTGTCGCTCTTCGTGCGGCTCTTGCGGCACTAGAAGGACGTGCCGTTGCGGTGATCGTCGATGGACACCTCCGTGCACCACGGGGTGTA

I N Y Y D S E K H A E N A V I F L H G N A T S S Y L W R H V V P H I>

___________________________TRANSLATION OF RLUC8-NRASQ61H FULL-LENGTH [A]____________________________>

210 220 230 240 250 260 270 280 290 300

CGAGCCCGTGGCCAGGTGCATCATCCCCGATCTGATCGGCATGGGCAAGAGCGGCAAGAGCGGCAACGGCAGCTACAGGCTGCTGGACCACTACAAGTAC

GCTCGGGCACCGGTCCACGTAGTAGGGGCTAGACTAGCCGTACCCGTTCTCGCCGTTCTCGCCGTTGCCGTCGATGTCCGACGACCTGGTGATGTTCATG

E P V A R C I I P D L I G M G K S G K S G N G S Y R L L D H Y K Y>

___________________________TRANSLATION OF RLUC8-NRASQ61H FULL-LENGTH [A]____________________________>

310 320 330 340 350 360 370 380 390 400

CTGACCGCCTGGTTCGAGCTCCTGAACCTGCCCAAGAAGATCATCTTCGTGGGCCACGACTGGGGCGCCGCCCTGGCCTTCCACTACGCCTACGAGCACC

GACTGGCGGACCAAGCTCGAGGACTTGGACGGGTTCTTCTAGTAGAAGCACCCGGTGCTGACCCCGCGGCGGGACCGGAAGGTGATGCGGATGCTCGTGG

L T A W F E L L N L P K K I I F V G H D W G A A L A F H Y A Y E H>

___________________________TRANSLATION OF RLUC8-NRASQ61H FULL-LENGTH [A]____________________________>

410 420 430 440 450 460 470 480 490 500

AGGACAGGATCAAGGCCATCGTGCACATGGAGAGCGTGGTGGACGTGATCGAGAGCTGGGACGAGTGGCCAGACATCGAGGAGGACATCGCCCTGATCAA

TCCTGTCCTAGTTCCGGTAGCACGTGTACCTCTCGCACCACCTGCACTAGCTCTCGACCCTGCTCACCGGTCTGTAGCTCCTCCTGTAGCGGGACTAGTT

Q D R I K A I V H M E S V V D V I E S W D E W P D I E E D I A L I K>

___________________________TRANSLATION OF RLUC8-NRASQ61H FULL-LENGTH [A]____________________________>

510 520 530 540 550 560 570 580 590 600

GAGCGAGGAGGGCGAGAAGATGGTGCTGGAGAACAACTTCTTCGTGGAGACCGTGCTGCCCAGCAAGATCATGAGAAAGCTGGAGCCCGAGGAGTTCGCC

CTCGCTCCTCCCGCTCTTCTACCACGACCTCTTGTTGAAGAAGCACCTCTGGCACGACGGGTCGTTCTAGTACTCTTTCGACCTCGGGCTCCTCAAGCGG

S E E G E K M V L E N N F F V E T V L P S K I M R K L E P E E F A>

___________________________TRANSLATION OF RLUC8-NRASQ61H FULL-LENGTH [A]____________________________>

610 620 630 640 650 660 670 680 690 700

GCCTACCTGGAGCCCTTCAAGGAGAAGGGCGAGGTGAGAAGACCCACCCTGAGCTGGCCCAGAGAGATCCCCCTGGTGAAGGGCGGCAAGCCCGACGTGG

CGGATGGACCTCGGGAAGTTCCTCTTCCCGCTCCACTCTTCTGGGTGGGACTCGACCGGGTCTCTCTAGGGGGACCACTTCCCGCCGTTCGGGCTGCACC

A Y L E P F K E K G E V R R P T L S W P R E I P L V K G G K P D V>

___________________________TRANSLATION OF RLUC8-NRASQ61H FULL-LENGTH [A]____________________________>

710 720 730 740 750 760 770 780 790 800

TGCAGATCGTGAGAAACTACAACGCCTACCTGAGAGCCAGCGACGACCTGCCCAAGCTGTTCATCGAGAGCGACCCCGGCTTCTTCAGCAACGCCATCGT

ACGTCTAGCACTCTTTGATGTTGCGGATGGACTCTCGGTCGCTGCTGGACGGGTTCGACAAGTAGCTCTCGCTGGGGCCGAAGAAGTCGTTGCGGTAGCA

V Q I V R N Y N A Y L R A S D D L P K L F I E S D P G F F S N A I V>

___________________________TRANSLATION OF RLUC8-NRASQ61H FULL-LENGTH [A]____________________________>

810 820 830 840 850 860 870 880 890 900

GGAGGGCGCCAAGAAGTTCCCCAACACCGAGTTCGTGAAGGTGAAGGGCCTGCACTTCCTCCAGGAGGACGCCCCCGACGAGATGGGCAAGTACATCAAG

CCTCCCGCGGTTCTTCAAGGGGTTGTGGCTCAAGCACTTCCACTTCCCGGACGTGAAGGAGGTCCTCCTGCGGGGGCTGCTCTACCCGTTCATGTAGTTC

E G A K K F P N T E F V K V K G L H F L Q E D A P D E M G K Y I K>

___________________________TRANSLATION OF RLUC8-NRASQ61H FULL-LENGTH [A]____________________________>

910 920 930 940 950 960 970 980 990 1000

AGCTTCGTGGAGAGAGTGCTGAAGAACGAGCAGCTCGAGGGCGGCGGAGGATCTGGGGGCGGAGGAAGTGGGGGAGGGGGCTCTGCGGCCGCTATGACTG

TCGAAGCACCTCTCTCACGACTTCTTGCTCGTCGAGCTCCCGCCGCCTCCTAGACCCCCGCCTCCTTCACCCCCTCCCCCGAGACGCCGGCGATACTGAC

S F V E R V L K N E Q L E G G G G S G G G G S G G G G S A A A M T>

___________________________TRANSLATION OF RLUC8-NRASQ61H FULL-LENGTH [A]____________________________>

1010 1020 1030 1040 1050 1060 1070 1080 1090 1100

AGTACAAACTGGTGGTGGTTGGAGCAGGTGGTGTTGGGAAAAGCGCACTGACAATCCAGCTGATCCAGAACCACTTTGTAGATGAATATGATCCCACCAT

TCATGTTTGACCACCACCAACCTCGTCCACCACAACCCTTTTCGCGTGACTGTTAGGTCGACTAGGTCTTGGTGAAACATCTACTTATACTAGGGTGGTA

E Y K L V V V G A G G V G K S A L T I Q L I Q N H F V D E Y D P T I>

___________________________TRANSLATION OF RLUC8-NRASQ61H FULL-LENGTH [A]____________________________>

1110 1120 1130 1140 1150 1160 1170 1180 1190 1200

AGAGGATTCTTACAGAAAACAAGTGGTTATAGATGGTGAAACCTGTTTGTTGGACATACTGGATACAGCTGGACATGAAGAGTACAGTGCCATGAGAGAC

TCTCCTAAGAATGTCTTTTGTTCACCAATATCTACCACTTTGGACAAACAACCTGTATGACCTATGTCGACCTGTACTTCTCATGTCACGGTACTCTCTG

E D S Y R K Q V V I D G E T C L L D I L D T A G H E E Y S A M R D>

___________________________TRANSLATION OF RLUC8-NRASQ61H FULL-LENGTH [A]____________________________>

1210 1220 1230 1240 1250 1260 1270 1280 1290 1300

CAATACATGAGGACAGGCGAAGGCTTCCTCTGTGTATTTGCCATCAATAATAGCAAGTCATTTGCGGATATTAACCTCTACAGGGAGCAGATTAAGCGAG

GTTATGTACTCCTGTCCGCTTCCGAAGGAGACACATAAACGGTAGTTATTATCGTTCAGTAAACGCCTATAATTGGAGATGTCCCTCGTCTAATTCGCTC

Q Y M R T G E G F L C V F A I N N S K S F A D I N L Y R E Q I K R>

___________________________TRANSLATION OF RLUC8-NRASQ61H FULL-LENGTH [A]____________________________>

1310 1320 1330 1340 1350 1360 1370 1380 1390 1400

TAAAAGACTCGGATGATGTACCTATGGTGCTAGTGGGAAACAAGTGTGATTTGCCAACAAGGACAGTTGATACAAAACAAGCCCACGAACTGGCCAAGAG

ATTTTCTGAGCCTACTACATGGATACCACGATCACCCTTTGTTCACACTAAACGGTTGTTCCTGTCAACTATGTTTTGTTCGGGTGCTTGACCGGTTCTC

V K D S D D V P M V L V G N K C D L P T R T V D T K Q A H E L A K S>

___________________________TRANSLATION OF RLUC8-NRASQ61H FULL-LENGTH [A]____________________________>

1410 1420 1430 1440 1450 1460 1470 1480 1490 1500

TTACGGGATTCCATTCATTGAAACCTCAGCCAAGACCAGACAGGGTGTTGAAGATGCTTTTTACACACTGGTAAGAGAAATACGCCAGTACCGAATGAAA

AATGCCCTAAGGTAAGTAACTTTGGAGTCGGTTCTGGTCTGTCCCACAACTTCTACGAAAAATGTGTGACCATTCTCTTTATGCGGTCATGGCTTACTTT

Y G I P F I E T S A K T R Q G V E D A F Y T L V R E I R Q Y R M K>

___________________________TRANSLATION OF RLUC8-NRASQ61H FULL-LENGTH [A]____________________________>

1510 1520 1530 1540 1550 1560

AAACTCAACAGCAGTGATGATGGGACTCAGGGTTGTATGGGATTGCCATGTGTGGTGATGTAA

TTTGAGTTGTCGTCACTACTACCCTGAGTCCCAACATACCCTAACGGTACACACCACTACATT

K L N S S D D G T Q G C M G L P C V V M *>

________TRANSLATION OF RLUC8-NRASQ61H FULL-LENGTH [A]__________>

Sequence: LMO2-RLuc8 Range: 1 to 1464

10 20 30 40 50 60 70 80 90 100

ATGAGTTCGGCCATCGAAAGGAAGAGCCTGGACCCGTCTGAGGAACCCGTGGATGAGGTGCTGCAGATACCCCCATCCCTGCTGACATGTGGTGGCTGCC

TACTCAAGCCGGTAGCTTTCCTTCTCGGACCTGGGCAGACTCCTTGGGCACCTACTCCACGACGTCTATGGGGGTAGGGACGACTGTACACCACCGACGG

M S S A I E R K S L D P S E E P V D E V L Q I P P S L L T C G G C>

___________________________________TRANSLATION OF LMO2-RLUC8 [A]____________________________________>

110 120 130 140 150 160 170 180 190 200

AGCAGAACATAGGGGACCGCTACTTCCTGAAAGCCATCGACCAGTACTGGCATGAGGATTGCCTCAGCTGTGACCTCTGTGGGTGTCGGCTGGGAGAGGT

TCGTCTTGTATCCCCTGGCGATGAAGGACTTTCGGTAGCTGGTCATGACCGTACTCCTAACGGAGTCGACACTGGAGACACCCACAGCCGACCCTCTCCA

Q Q N I G D R Y F L K A I D Q Y W H E D C L S C D L C G C R L G E V>

___________________________________TRANSLATION OF LMO2-RLUC8 [A]____________________________________>

210 220 230 240 250 260 270 280 290 300

GGGGAGGCGCCTCTACTACAAGCTGGGACGGAAATTGTGCAGGAGAGACTATCTCAGGCTTTTTGGTCAGGATGGTCTCTGTGCATCCTGTGACAAGCGG

CCCCTCCGCGGAGATGATGTTCGACCCTGCCTTTAACACGTCCTCTCTGATAGAGTCCGAAAAACCAGTCCTACCAGAGACACGTAGGACACTGTTCGCC

G R R L Y Y K L G R K L C R R D Y L R L F G Q D G L C A S C D K R>

___________________________________TRANSLATION OF LMO2-RLUC8 [A]____________________________________>

310 320 330 340 350 360 370 380 390 400

ATCCGTGCCTATGAGATGACGATGCGGGTGAAAGACAAAGTGTATCACCTGGAGTGTTTCAAATGCGCCGCCTGTCAGAAGCATTTCTGTGTAGGTGACA

TAGGCACGGATACTCTACTGCTACGCCCACTTTCTGTTTCACATAGTGGACCTCACAAAGTTTACGCGGCGGACAGTCTTCGTAAAGACACATCCACTGT

I R A Y E M T M R V K D K V Y H L E C F K C A A C Q K H F C V G D>

___________________________________TRANSLATION OF LMO2-RLUC8 [A]____________________________________>

410 420 430 440 450 460 470 480 490 500

GATACCTTCTCATCAACTCCGACATAGTGTGTGAACAAGACATCTACGAGTGGACTAAGATCAATGGGATGATACTCGAGGGCGGTGGCGGATCGGGCGG

CTATGGAAGAGTAGTTGAGGCTGTATCACACACTTGTTCTGTAGATGCTCACCTGATTCTAGTTACCCTACTATGAGCTCCCGCCACCGCCTAGCCCGCC

R Y L L I N S D I V C E Q D I Y E W T K I N G M I L E G G G G S G G>

___________________________________TRANSLATION OF LMO2-RLUC8 [A]____________________________________>

510 520 530 540 550 560 570 580 590 600

AGGTGGCAGTGCGGCCGCAGGGAGTGGTATGACCAGCAAGGTGTACGACCCCGAGCAGAGGAAGAGGATGATCACCGGCCCCCAGTGGTGGGCCAGGTGC

TCCACCGTCACGCCGGCGTCCCTCACCATACTGGTCGTTCCACATGCTGGGGCTCGTCTCCTTCTCCTACTAGTGGCCGGGGGTCACCACCCGGTCCACG

G G S A A A G S G M T S K V Y D P E Q R K R M I T G P Q W W A R C>

___________________________________TRANSLATION OF LMO2-RLUC8 [A]____________________________________>

610 620 630 640 650 660 670 680 690 700

AAGCAGATGAACGTGCTGGACAGCTTCATCAACTACTACGACAGCGAGAAGCACGCCGAGAACGCCGTGATCTTCCTGCACGGCAACGCCACTAGCAGCT

TTCGTCTACTTGCACGACCTGTCGAAGTAGTTGATGATGCTGTCGCTCTTCGTGCGGCTCTTGCGGCACTAGAAGGACGTGCCGTTGCGGTGATCGTCGA

K Q M N V L D S F I N Y Y D S E K H A E N A V I F L H G N A T S S>

___________________________________TRANSLATION OF LMO2-RLUC8 [A]____________________________________>

710 720 730 740 750 760 770 780 790 800

ACCTGTGGAGGCACGTGGTGCCCCACATCGAGCCCGTGGCCAGGTGCATCATCCCCGATCTGATCGGCATGGGCAAGAGCGGCAAGAGCGGCAACGGCAG

TGGACACCTCCGTGCACCACGGGGTGTAGCTCGGGCACCGGTCCACGTAGTAGGGGCTAGACTAGCCGTACCCGTTCTCGCCGTTCTCGCCGTTGCCGTC

Y L W R H V V P H I E P V A R C I I P D L I G M G K S G K S G N G S>

___________________________________TRANSLATION OF LMO2-RLUC8 [A]____________________________________>

810 820 830 840 850 860 870 880 890 900

CTACAGGCTGCTGGACCACTACAAGTACCTGACCGCCTGGTTCGAGCTCCTGAACCTGCCCAAGAAGATCATCTTCGTGGGCCACGACTGGGGCGCCGCC

GATGTCCGACGACCTGGTGATGTTCATGGACTGGCGGACCAAGCTCGAGGACTTGGACGGGTTCTTCTAGTAGAAGCACCCGGTGCTGACCCCGCGGCGG

Y R L L D H Y K Y L T A W F E L L N L P K K I I F V G H D W G A A>

___________________________________TRANSLATION OF LMO2-RLUC8 [A]____________________________________>

910 920 930 940 950 960 970 980 990 1000

CTGGCCTTCCACTACGCCTACGAGCACCAGGACAGGATCAAGGCCATCGTGCACATGGAGAGCGTGGTGGACGTGATCGAGAGCTGGGACGAGTGGCCAG

GACCGGAAGGTGATGCGGATGCTCGTGGTCCTGTCCTAGTTCCGGTAGCACGTGTACCTCTCGCACCACCTGCACTAGCTCTCGACCCTGCTCACCGGTC

L A F H Y A Y E H Q D R I K A I V H M E S V V D V I E S W D E W P>

___________________________________TRANSLATION OF LMO2-RLUC8 [A]____________________________________>

1010 1020 1030 1040 1050 1060 1070 1080 1090 1100

ACATCGAGGAGGACATCGCCCTGATCAAGAGCGAGGAGGGCGAGAAGATGGTGCTGGAGAACAACTTCTTCGTGGAGACCGTGCTGCCCAGCAAGATCAT

TGTAGCTCCTCCTGTAGCGGGACTAGTTCTCGCTCCTCCCGCTCTTCTACCACGACCTCTTGTTGAAGAAGCACCTCTGGCACGACGGGTCGTTCTAGTA

D I E E D I A L I K S E E G E K M V L E N N F F V E T V L P S K I M>

___________________________________TRANSLATION OF LMO2-RLUC8 [A]____________________________________>

1110 1120 1130 1140 1150 1160 1170 1180 1190 1200

GAGAAAGCTGGAGCCCGAGGAGTTCGCCGCCTACCTGGAGCCCTTCAAGGAGAAGGGCGAGGTGAGAAGACCCACCCTGAGCTGGCCCAGAGAGATCCCC

CTCTTTCGACCTCGGGCTCCTCAAGCGGCGGATGGACCTCGGGAAGTTCCTCTTCCCGCTCCACTCTTCTGGGTGGGACTCGACCGGGTCTCTCTAGGGG

R K L E P E E F A A Y L E P F K E K G E V R R P T L S W P R E I P>

___________________________________TRANSLATION OF LMO2-RLUC8 [A]____________________________________>

1210 1220 1230 1240 1250 1260 1270 1280 1290 1300

CTGGTGAAGGGCGGCAAGCCCGACGTGGTGCAGATCGTGAGAAACTACAACGCCTACCTGAGAGCCAGCGACGACCTGCCCAAGCTGTTCATCGAGAGCG

GACCACTTCCCGCCGTTCGGGCTGCACCACGTCTAGCACTCTTTGATGTTGCGGATGGACTCTCGGTCGCTGCTGGACGGGTTCGACAAGTAGCTCTCGC

L V K G G K P D V V Q I V R N Y N A Y L R A S D D L P K L F I E S>

___________________________________TRANSLATION OF LMO2-RLUC8 [A]____________________________________>

1310 1320 1330 1340 1350 1360 1370 1380 1390 1400

ACCCCGGCTTCTTCAGCAACGCCATCGTGGAGGGCGCCAAGAAGTTCCCCAACACCGAGTTCGTGAAGGTGAAGGGCCTGCACTTCCTCCAGGAGGACGC

TGGGGCCGAAGAAGTCGTTGCGGTAGCACCTCCCGCGGTTCTTCAAGGGGTTGTGGCTCAAGCACTTCCACTTCCCGGACGTGAAGGAGGTCCTCCTGCG

D P G F F S N A I V E G A K K F P N T E F V K V K G L H F L Q E D A>

___________________________________TRANSLATION OF LMO2-RLUC8 [A]____________________________________>

1410 1420 1430 1440 1450 1460

CCCCGACGAGATGGGCAAGTACATCAAGAGCTTCGTGGAGAGAGTGCTGAAGAACGAGCAGTAA

GGGGCTGCTCTACCCGTTCATGTAGTTCTCGAAGCACCTCTCTCACGACTTCTTGCTCGTCATT

P D E M G K Y I K S F V E R V L K N E Q *>

_________________TRANSLATION OF LMO2-RLUC8 [A]__________________>
